# Augmented germinal center and antibody responses to HIV Env trimers delivered as transmembrane immunogens by self-replicating RNA

**DOI:** 10.1016/j.ymthe.2025.07.036

**Published:** 2025-07-29

**Authors:** Parisa Yousefpour, Amrit Raj Ghosh, Himanshi Chawla, Rachel Yeung, Justin Gregory, Kristen Si, Tanaka K. Remba, Kristen A. Rodrigues, Mariane B. Melo, Jonathan Dye, Jon M. Steichen, Yuebao Zhang, Yizhou Dong, Max Crispin, William R. Schief, Facundo D. Batista, Darrell J. Irvine

**Affiliations:** 1 Koch Institute for Integrative Cancer Research, Massachusetts Institute of Technology, Cambridge, MA, USA.; 2 Ragon Institute of Massachusetts General Hospital, Massachusetts Institute of Technology and Harvard University, Cambridge, MA, USA.; 3 Consortium for HIV/AIDS Vaccine Development, The Scripps Research Institute; La Jolla, CA 92037 USA.; 4 School of Biological Sciences, University of Southampton, Southampton SO17 1BJ, UK.; 5 Harvard-MIT Health Sciences and Technology Program, Institute for Medical Engineering and Science, Massachusetts Institute of Technology, Cambridge, MA 02139, USA.; 6 Department of Immunology and Microbiology, The Scripps Research Institute, La Jolla, CA 92037, USA.; 7 IAVI Neutralizing Antibody Center, The Scripps Research Institute, La Jolla, CA 92037, USA.; 8 Moderna, Inc. Cambridge, MA 02139, USA; 9 Division of Pharmaceutics & Pharmacology, College of Pharmacy, The Ohio State University, Columbus, OH 43210, USA.; 10 Icahn Genomics Institute, Precision Immunology Institute, Department of Immunology and Immunotherapy, Department of Oncological Sciences, Tisch Cancer Institute, Friedman Brain Institute, Biomedical Engineering and Imaging Institute, Icahn School of Medicine at Mount Sinai, New York, NY, 10029, USA.; 11 Department of Biology, Massachusetts Institute of Technology, Cambridge, MA 02139, USA.; 12 Department of Biological Engineering, Massachusetts Institute of Technology, Cambridge, MA, USA.; 13 Howard Hughes Medical Institute, Chevy Chase, MD, USA.

**Keywords:** replicon RNA, expression form, HIV trimer, RNA vaccines, HIV bnAbs, BG18

## Abstract

mRNA vaccines have emerged as an important platform for vaccine development. Unlike protein subunit vaccines, mRNA-expressed antigens can be expressed in either secreted or transmembrane (TM) forms mimicking a viral envelope (Env) protein. Here, we investigated the impact of antigen expression format on the antigenicity profile, glycosylation, and immunogenicity of stabilized HIV Env trimer immunogens expressed from self-replicating RNA (replicon) vaccines. Replicon-encoded trimers in both forms exhibited proper folding, and replicon-expressed secreted trimers exhibited glycosylation patterns largely consistent with recombinant trimer protein, though with enrichment of complex glycans over high mannose at some sites. Both formats were highly immunogenic in mice, eliciting comparable serum antibody and T cell responses. Interestingly, the TM format initiated smaller germinal center (GC) responses, but these GCs were enriched for trimer-binding B cells compared to secreted trimer vaccines. In a B cell receptor knock-in adoptive transfer model for assessing germline targeting, replicon-encoded TM trimer elicited a greater frequency of epitope-targeting antibodies and recruited broadly neutralizing antibody precursor B cells to the GC response more efficiently compared to replicon-encoded secreted trimer or protein trimer combined with adjuvant. These results indicate that the form of immunogen expression can impact key elements of immune responses to RNA vaccines.

## Graphical Abstract

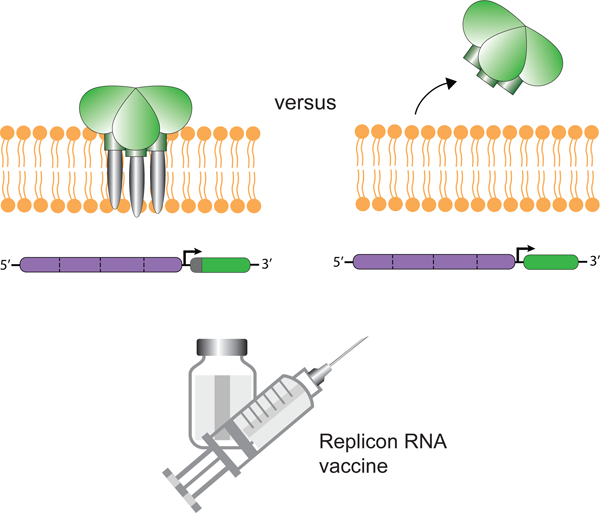

## INTRODUCTION

Vaccines comprised of *in vitro* transcribed and capped antigen-encoding RNA encapsulated in lipid nanoparticles have emerged as an important platform for current and future vaccine development. The rapid development of effective vaccines for the COVID-19 pandemic demonstrated the potential and versatility of RNA, which is a platform technology enabling the design of new vaccines using genetic sequence information without requiring alterations to the production process or facilities.^1,2^ Two types of RNA vaccines have established clinical efficacy against SARS-CoV-2, those based on mRNA employing modified bases to enhance RNA stability and reduce innate immune recognition,^3,4^ and vaccines based on alphavirus-derived self-replicating RNAs.^5–8^ Self-replicating (replicon) RNAs encode a set of non-structural proteins (nsPs) in addition to a payload gene of interest. The nsPs form an RNA-dependent RNA polymerase that can copy both the overall RNA sequence and an encoded subgenome containing the antigen payload.^6^ Replicons are of great interest for RNA vaccination, as they have the potential to enable much lower total doses of RNA to be used,^9–12^ and concomitantly, lower doses of lipid delivery vehicle, which can increase tolerability and enable dose sparing. A recent clinical trial has also indicated potential benefits of heterologous immunization with replicons followed by non-replicating mRNA SARS-CoV-2 vaccines.^13^

In addition to different modalities of RNA, nucleic acid vaccines also open up distinct antigen format possibilities in vaccine design. For example, viral envelope antigens can be expressed from RNA as secreted proteins or in a native transmembrane form— as in the approved Pfizer and Moderna COVID-19 vaccines. However, studies investigating the impact of the expression form of the immunogen (e.g., transmembrane versus secreted antigens) on immune responses to vaccination are currently limited. In a humanized mouse model of HIV vaccination, we previously showed that immunization with mRNA encoding transmembrane envelope (Env) trimer antigen was more effective at activating low-affinity B cell precursors than immunization with the same trimer in the form of a soluble recombinant protein antigen combined with adjuvant.^14^ Aldon et al. reported that a transmembrane Env trimer immunogen elicited more rapid IgG binding titers and a skewing toward IgG2a class switching compared to an equivalent soluble Env trimer when delivered as replicon RNA in mice.^15^ However, whether the immunogen format impacts other elements of the immune response primed by replicon vaccines such as germinal center (GC) responses and memory or plasma cell development, to our knowledge, has not been evaluated. In addition, we sought to investigate how antigen expression from rapidly self-replicating RNAs affects antigen folding and glycosylation of complex immunogens such as HIV Env trimers.

To address these questions, here we studied the impact of the expression form on different facets of the immune response to lipid nanoparticle-delivered replicon RNA encoding a stabilized HIV Env germline targeting immunogen. Using a Venezuelan Equine Encephalitis virus-based replicon backbone we previously developed,^16,17^ we designed replicons encoding secreted or transmembrane (TM) forms of Env trimers. For these studies, we selected the N332-GT2 trimer. This is a germline-targeting immunogen engineered to engage B cell precursors capable of targeting a glycan supersite centered on N332 in the base of the V3 loop of Env, which is the most common target for broadly neutralizing antibodies (bNAbs) in HIV-1 infection, and is well-conserved, occurring in 73% of 30,000 cross-clade Env isolates.^18,19^ In addition to mutations enabling precursor B cells to bind the N332 supersite, the trimer sequence includes mutations designed to highly stabilize the overall trimer structure.^20^ HIV trimers can be produced in a secreted form by expressing the envelope lacking the native transmembrane domain of Env gp160, or in a transmembrane form by including the native Env transmembrane domain truncated at the C-terminus to optimize cell surface expression.^18,20^ To compare the properties and immunogenicity of these two forms of trimer expressed by replicons, N332-GT2 secreted or TM sequences were cloned into a VEE-based replicon backbone we previously described.^16,17^

We found that both forms exhibited well-folded conformations, effectively displaying broadly neutralizing epitopes with limited exposure of non-neutralizing sites. Examining immune responses to these replicon-encoded immunogens, we found that both forms elicited strong and comparable T cell and antibody responses; however, the TM form triggered more selective germinal center (GC) responses, enriched with antigen-binding B cells, compared to the secreted form. Additionally, the TM format more efficiently recruited broadly neutralizing antibody (bnAb) precursor B cells early in the GC response, and elicited higher titers for IgG3 antibody subclass. Thus, these studies provide further support for the use of TM immunogen designs for priming of productive immune responses against HIV.

## RESULTS

### Stabilized HIV Env trimer immunogens expressed from replicons have immunogenicity and glycan profiles similar to recombinant protein

**Figure 1.**
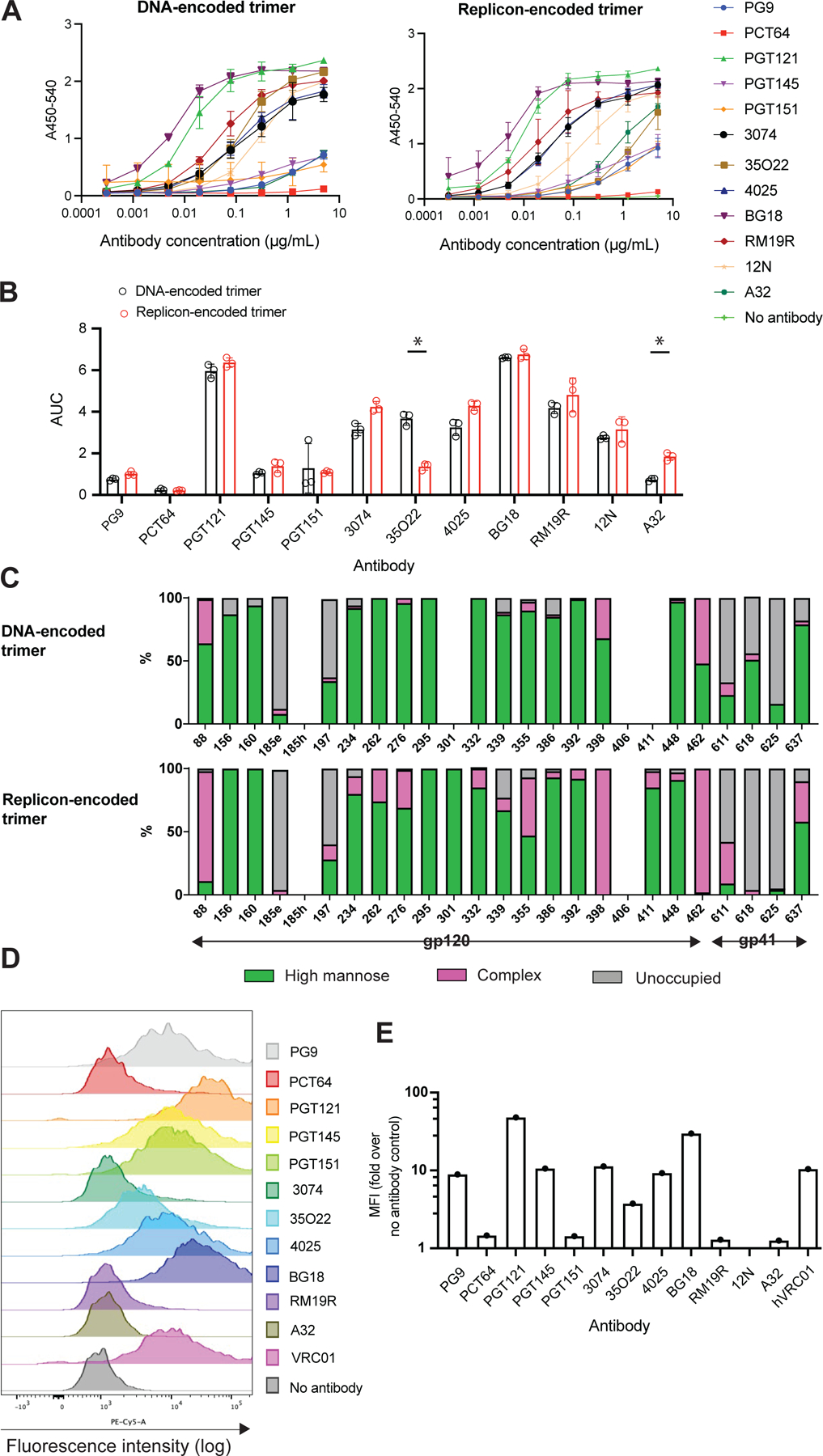
Characterization of replicon-encoded trimers. (A-B) Antigenicity profiling of secreted trimer. Secreted trimers were expressed from replicons (electroporated into BHK-21 cells) and from plasmid DNA (transfected into HEK Expi293 cells by lipofection). Trimers were collected from the culture supernatant, and their binding to various antibodies was assessed by ELISA. Results are presented as (A) absorbance at 450 nm with correction at 540 nm (A450–540) and (V) area under the curve (AUC). (C) Site-specific compositions of N-linked glycans located on the secreted trimer encoded from replicon or plasmid DNA in HEK Expi293 cells. Data could not be obtained for listed sites where no bar graph is displayed. (D-E) Antigenicity profiling of transmembrane trimers. Shown are (D) flow cytometry plots and (E) median florescence intensity (MFI) of C2C12 cells transfected with replicon by electroporation and expressing the transmembrane trimer, following staining with antibodies targeting different epitopes on the trimer. Data are shown as means ± standard deviation. Statistical significance was determined by student’s t-test.

We first compared the antigenicity profile of the secreted N332-GT2 trimer expressed by BHK-21 cells transfected with replicons via electroporation to that of trimers produced as traditional recombinant proteins from HEK Expi293 cells transfected with plasmid DNA encoding the same sequence via lipofection. Trimer in the supernatants collected from cells transfected with replicons or DNA was quantified by ELISA, yielding approximately 15 ng and 34 ng per 1,000 cells for replicon- and plasmid DNA-transfected cells, respectively. Equivalent amounts of trimer from each platform were then captured on ELISA plates coated with the CD4 binding site-specific antibody VRC01, and probed for binding to serial dilutions of a panel of neutralizing and non-neutralizing monoclonal antibodies recognizing epitopes covering many different sites on the trimer. This characterization revealed comparable antibody binding profiles for secreted trimers expressed from replicons versus DNA-expressed trimer for the V1/V2 loops, interface/fusion peptide, and V3 loop epitopes (Fig. 1A–B). The most notable differences were reduced binding of the gp120/gp41 interface bnAb 35O22, and slightly increased binding of the non-neutralizing antibody A32. Importantly, binding by the bnAb BG18, which N332-GT2 trimer is designed to bind to with high affinity, was high and equivalent for both recombinant protein and replicon-expressed trimer.

The glycan shield of Env trimers plays a major role in shaping the antibody response. Based on the promising evidence from antigenicity profiling that the replicon produces a well-folded secreted trimer, we next compared the glycan profile of secreted trimers produced by HEK cells transfected with DNA (traditional recombinant protein expression) vs. HEK cells transfected with replicon. Combined liquid chromatography mass spectrometry analysis of trimer recovered from the supernatant of transfected cells^21–23^ showed that the glycan profiles of replicon- and DNA-expressed N332-GT2 were overall very similar (Fig. 1C). We observed a high abundance of oligomannose-type glycans at intrinsic mannose patch sites focused around the N332 supersite.^24,25^ Furthermore, the trimer-associated mannose patch, focused around the N156 and N160 glycans, was conserved in replicon-expressed Env, which is an indicator of folding fidelity (Fig. 1C).^26,27^ These features represent native-like trimeric compositions. A few N-glycosylation sites were less occupied including N185e, N197 and across gp41 sites. Interestingly, replicon-expressed Env presented a higher proportion of complex-type glycans than plasmid DNA-expressed Env at a few sites including N88, N355, N398, and N462 (Fig. 1C). Altogether, the antigenic profile and glycan composition of replicon-expressed and recombinant DNA-expressed N332-GT2 were largely similar, suggesting that replicon expression did not dramatically alter trimer folding or glycan processing.

We also carried out antigenic profiling of transmembrane N332-GT2, by surface staining replicon-electroporated murine myoblast C2C12 cells (chosen to model intramuscular delivery, a preferred route for mRNA vaccines^1,28^) with the panel of neutralizing and non-neutralizing monoclonal antibodies for analysis by flow cytometry. Similar to secreted trimer, the membrane-bound trimer exhibited strong binding to antibodies targeting bnAb epitopes, including BG18, VRC01, and trimer-specific quarternary epitopes (PGT121 and PGT145, Fig. 1D–E). By contrast, the TM trimer exhibited low binding to the non-neutralizing gp120 antibody A32 and the trimer base-specific antibody RM19R (Fig. 1D–E). These data suggest the replicon-expressed TM trimer is also folded properly and presenting expected neutralizing sites.

### TM trimer replicons elicit a greater proportion of antigen-specific GC B cells but a smaller overall GC than secreted trimer immunization

**Figure 2.**
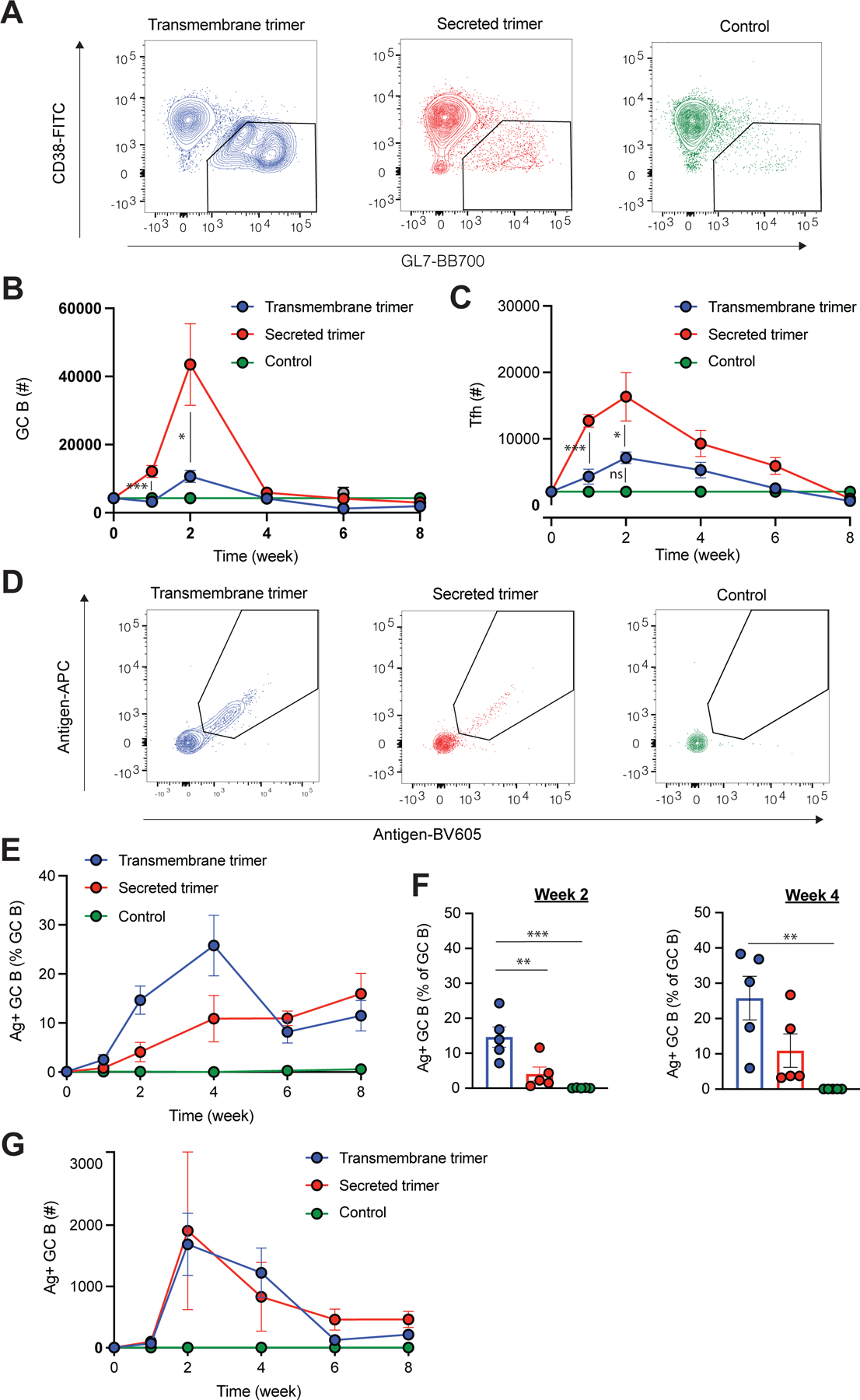
Secreted trimer replicons elicit larger total GC responses, but TM trimer replicons promote a greater frequency of trimer-binding GC B cells. Total GC B cells, antigen-specific GC B cell and Tfh populations were assessed in draining lymph nodes at various time points after a single immunization of BALB/c mice with LNP-replicons encoding the transmembrane or secreted trimer. **(A)** Representative flow cytometry contour plots showing GC B cell staining gated on B cells from mice with indicated treatments at week 2 post immunization. **(B-C)** Graphs show the numbers of GL7^+^CD38^+^GC B cells (B) and CXCR5^+^PD-1^+^ Tfh cells (C) over time in mice treated with LNP-replicons. **(D-G)** Kinetics of antigen-specific GC B cell responses in draining lymph nodes. Shown are (D) the representative flow cytometry contour plots showing antigen staining gated on GC B cells from mice with indicated treatments at week 2 post immunization, (E) percentage of antigen-specific GC B cells out of total B cells over time, (F) percentage of antigen-specific GC B cells at week 2 and 4 post immunization, and (G) number of antigen-specific GC B cells over time. Statistical significance was assessed by two-way analysis of variance (ANOVA) followed by Tukey’s post hoc test. Data are shown as mean ± SEM; *p < 0.05, **p < 0.01, and ***p < 0.001.

To assess immune responses triggered by secreted vs. TM trimer immunization, we encapsulated replicons encoding each construct form in lipid nanoparticles (LNPs), using a formulation we previously reported for effective replicon delivery in mice.^17^ After encapsulation and buffer exchange into PBS, the LNPs exhibited an average median diameter of ~70–100 nm across batches, based on the number distribution from dynamic light scattering and corroborated by cryo-TEM, with a mean zeta potential of approximately –7 mV, and RNA encapsulation efficiency of above 95% (Supplementary Fig. S1A–E). These characteristics were reproducibly maintained across independent batches of replicon/LNPs, with no significant differences observed either across batches or between LNPs encapsulating TM trimer versus secreted trimer replicon constructs. We first characterized the development of antigen-specific B cell responses and germinal center (GC) responses by flow cytometry (Supplementary Fig. S2A). BALB/c mice were immunized once intramuscularly (i.m.) with 2 μg replicons encoding secreted or TM trimer, and draining lymph nodes (dLNs) were analyzed over time. Notably, the use of replicons encoding the secreted form of the immunogen led to substantially higher numbers of Tfh and total GC B cells at 2 weeks post immunization (Fig. 2A–C, Supplementary Fig. S3A–E). GCs contracted substantially by 4 weeks in all groups. Although the TM trimer-encoding replicon elicited much smaller total GC responses, when we stained GC B cells with fluorescent antigen tetramers, we found that TM trimer replicons elicited a substantially greater frequency of antigen-binding GC B cells, which persisted through week 4 post immunization (Fig. 2D–F). The combination of these effects led the total antigen-binding GC B cell response over time to be equivalent for the two immunogen formats (Fig. 2G). Outside the germinal centers, neither the secreted nor the transmembrane (TM) trimer encoded by replicons led to a significant increase in the number of antigen-binding B cells compared to naïve control groups, indicating that replicon immunization elicited little or no extrafollicular B cell response (Supplementary Fig. S4). We hypothesize that more antigen arrives at the draining LN for replicon-generated secreted trimer, but that the TM trimer may be more stable and provide more intact antigen to drive the GC B cell response.

### Replicons encoding TM trimer elicit elevated inflammatory cytokine and chemokine production in draining lymph nodes compared to secreted trimer replicons

**Figure 3.**
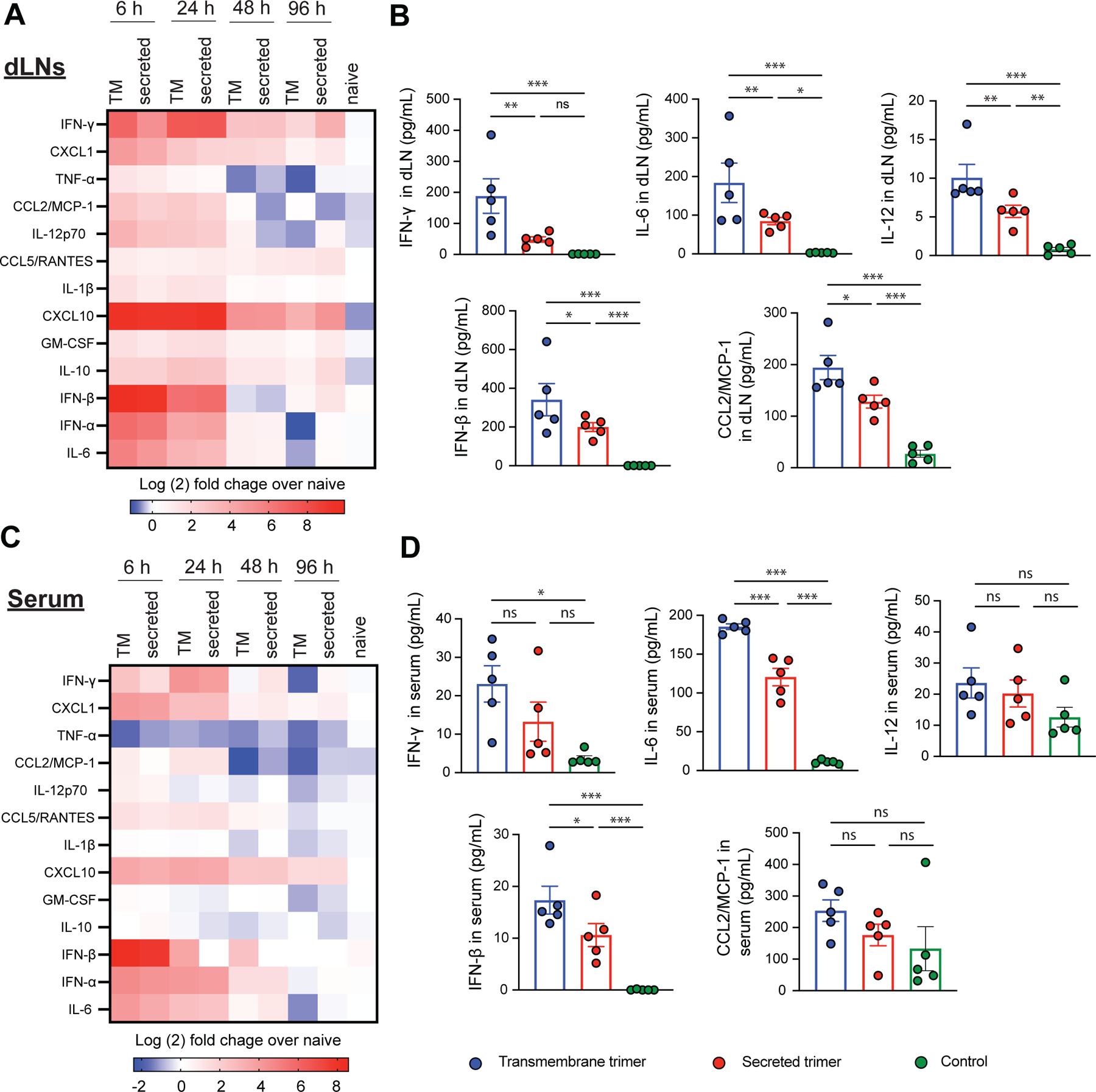
Replicons encoding TM trimer immunogen elicit amplified cytokines responses in draining lymph nodes and blood. BALB/c mice were immunized with LNP-replicon intramuscularly. Draining lymph nodes (dLNs) and serum samples were harvested at 6-, 24-, 48- and 96-hours post immunization, processed and their cytokine content was measured by LEGENDplex antiviral response assay. (A) Heatmap showing the cytokine expression levels, normalized to the corresponding levels in untreated animals in draining lymph nodes, in response to vaccination with LNP-replicons encoding the transmembrane and secreted form of the trimer. (B) Concentrations of selected cytokines in dLNs at 6 h post vaccination. (C) Heatmap showing cytokine expression levels, normalized to the corresponding levels in untreated animals in serum, in response to vaccination. (D) Concentration of selected cytokines in serum at 6 h post vaccination. Individual data points corresponding to each animal are plotted alongside mean values ± SEM (*n* = 5 animals/group). Statistical significance was assessed by two-way analysis of variance (ANOVA) followed by Tukey’s post hoc test. *p < 0.05, **p < 0.01, and ***p < 0.001.

To shed light on the differential GC responses observed for replicon immunizations with the two immunogen formats, we examined cytokine and chemokine (CK) expression in the dLNs and blood over time. This analysis revealed two waves of cytokine/CK responses in the dLN– short lived responses that were elevated at 6 and 24 hr but largely subsided by 48 hr (e.g., TNF-α, CCL2, IFN-β), and more sustained responses that continued to be elevated above baseline through 96 hr (e.g., IFN-γ, CXCL10, CXCL1, IL-10, Fig. 3A). Quantitatively, immunization with the TM trimer replicon elicited statistically significantly higher levels of several cytokines/CK, including IFN-γ, IL-12, IFN-β, IL-6, and CCL2 (Fig. 3B). We also analyzed cytokine/CK levels in the blood, and found that most inflammatory protein levels there tracked responses in the dLN, though the levels of most of the proteins measured were returning to baseline by 48 hr (Fig. 3C–D). Type I interferons were notably upregulated early after immunization (Fig. 3C–D). Strikingly, these data indicate that simply changing the format of the encoded immunogen can alter the inflammatory environment established in the dLN following replicon immunization.

### Both secreted and TM trimer immunogen replicon immunizations prime strong T cell and antibody responses in mice

**Figure 4.**
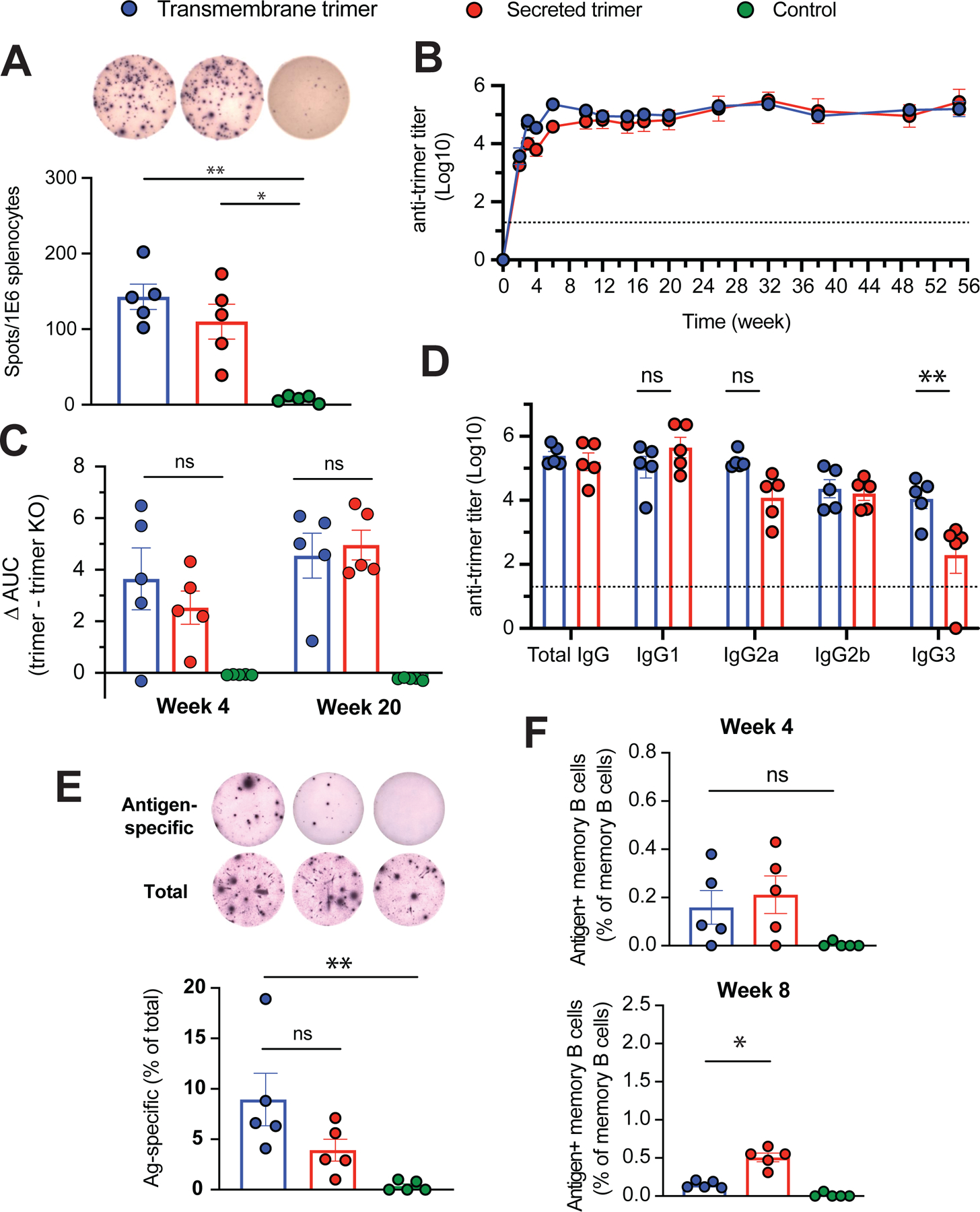
Cellular and humoral immune responses to vaccination. BALB/c mice were immunized with a single dose of LNP-replicon or proteins. (A) Antigen-specific T cell responses. Splenocytes were collected at week 8 posit immunization and T-cell responses to transmembrane and secreted N332-GT2 antigen encoded from replicons were evaluated by IFN-γ ELISPOT assay with overlapping peptides spanning the entire trimer. (B) anti-trimer antibody titer after vaccination. Serum samples were collected longitudinally and analyzed for trimer-binding antibodies by ELISA. The dashed line indicates the background response in naïve mice. (C) Epitope specificity of antibody responses, Serum samples at week 4 and 20 post immunization were assessed for epitope specificity using ELISA by calculating the differences in area under the curves (AUCs) for binding to the N332-GT2 trimer compared to the N332-GT2-KO (an epitope knockout) trimer. (D) Subclasses of IgG antibodies elicited. Data are from blood samples collected at Week 15 post immunization. The dashed line indicates the background response in naïve mice. (E) Induction of antibody secreting cells in bone marrow. Cells were collected from marrow of both femurs and tibias at week 10 post immunization, and the frequency of antibody secreting cells was determined by total as well as antigen-specific IgG ELISpot assays. (F) Quantification of the antigen-specific memory B cells was conducted at week 4 and 8 post immunization by harvesting and processing spleen, followed staining and gating on relevant markers by flow cytometry. Data in panel A, (B-C), D, and E are from independent experiments. Statistical significance was assessed by two-way analysis of variance (ANOVA) followed by Tukey’s post hoc test. Data are shown as mean ± SEM; *p < 0.05, and **p < 0.01.

We next examined several outputs of the humoral and cellular responses to vaccination over time. At 8 weeks following a single immunization with LNP-replicons containing 2 μg of replicon RNA, restimulation of T cells from the spleens of mice revealed comparable induction of trimer-specific memory T cell responses for both TM and secreted N332-GT2 trimer (Fig. 4A). LNP-replicon immunization also elicited high titers of serum IgG against the trimer in both immunogen formats that plateaued at approximately 8 weeks post immunization (Fig. 4B). At lower doses of the replicon, a lower titer was observed, but it increased over time (Supplementary Fig. S5A). These robust responses are consistent with the properties of the glycan-unmasked germline-targeting immunogens, which differ substantially from native-like Env trimers that are heavily glycosylated with a ‘glycan shield’ that limits antigen accessibility and immunogenicity.^18,29^ We also tested a non-secreted intracellular form of the trimer, and consistent with expectations, this replicon resulted in negligible serum antibody responses, confirming that antigen exposure via secretion or membrane display is required for immunogenicity (Supplementary Fig. S5B). To evaluate the specificity of the antibody response toward the BG18 epitope—a broadly neutralizing antibody site of interest—we performed ELISAs using an epitope knockout variant (N332-GT2KO) and calculated ΔAUC values by subtracting the binding AUC of the KO trimer from that of the wild-type N332-GT2 trimer. This analysis revealed no significant difference in BG18 epitope-directed antibody responses between the TM and secreted trimer replicons (Fig. 4C). Both TM and secreted trimer replicons induced class switching to all four subclasses of IgG, with TM trimer replicon eliciting a stronger IgG3 response and a trend toward stronger induction of IgG2a (Fig. 4D).

We next assessed the development of long-lived plasma cells in the bone marrow. ELISpot analysis showed that both secreted and TM trimer immunogen formats led to induction of significant trimer-specific antibody-secreting cells (ASCs) in the bone marrow at 10 weeks post immunization, with a trend toward higher responses for the TM trimer immunogen (Fig. 4E). By contrast, antigen tetramer staining revealed comparable antigen-specific memory B cell development at 4 weeks post immunization, but memory cells further expanded by week 8 for the secreted trimer format (Fig. 4F and Supplementary Fig. S2B). Thus, both TM and secreted trimer replicons primed strong T cell and antibody responses, with some differences in the long-term output of plasma cell vs. memory B cell responses.

### TM trimer immunogens trigger enhanced early recruitment of bnAb precursor B cells to germinal centers compared to secreted trimer replicons

**Figure 5.**
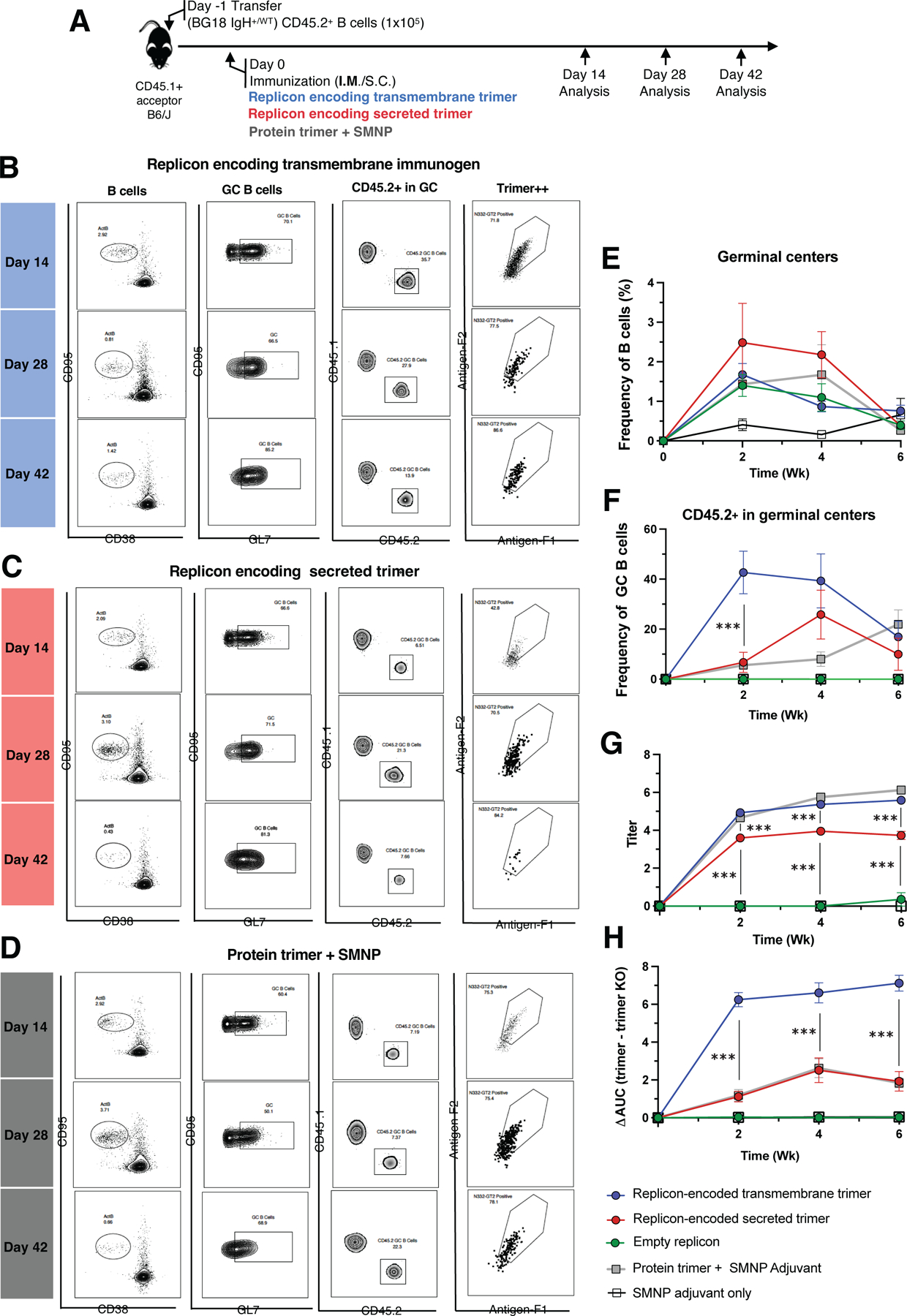
BG18 responses can be elicited by replicons in a precursor knockin mouse model. 1 x 10^5^ B cells from BG18 IgH^+/WT^ CD45.2^+^ mice were adoptively transferred into CD45.1^+^ recipient mice and were used in downstream immunization experiments. (A) Schematic of adoptive transfer (day -1) followed by immunization I.M. (day 0) and analysis (day 14, 28, and 42). (B–C) FACS plots indicating ActB (activated B cells) cells gated on Lymphocytes/ Single Cells/ Live cells (SSL^+^/Live/Dump^−^/B220^+^/CD38^−^/CD95+), GC B cells (CD95^+^/GL7^+^ ActB cells), CD45.2^+^ cells in GCs, and antigen specificity of CD45.2+ GC B cells for 14, 28 and 42 days post immunization by the replicon encoding secreted immunogen (B), replicon encoding membrane-bound immunogen (C), protein immunization with SMNP adjuvant. (E-F) Quantification of B cells in GCs (E) and CD45.2+ B cells in GCs (F); each condition had 6 mice per group. (G-H) Evaluation of antibody responses: Serum samples were longitudinally collected from CD45.1^+^ recipient mice. Antibody levels were measured by ELISA, and reported as titers (G), and epitope specificity of antibodies were assessed using ELISA by calculating the differences in area under the curves (AUCs) for binding to the N332-GT2 trimer compared to the N332-GT2-KO (an epitope knockout) trimer (H). Statistical significance was assessed by two-way analysis of variance (ANOVA) followed by Tukey’s post hoc test. Data are shown as mean ± SEM; ***p < 0.001.

The N332-GT2 trimer is designed to engage precursor B cells capable of evolving their antigen receptor to produce bnAbs targeting the N332 supersite of HIV Env similar to the bnAb BG18.^18^ To test how secreted vs. TM forms of this germline targeting immunogen compared for priming BG18 precursor B cells, we employed an adoptive transfer BCR knock-in model we recently developed for this system: BG18^gH^ mice have the inferred germline Ig heavy chain of BG18 knocked in to the BCR locus^23^, such that ~30–32% of B cells from these mice express the germline BG18 heavy chain variable regions paired with native murine light chains, producing functional B cell receptors (BCRs) capable of recapitulating BG18 bnAb maturation.^18^ To establish low, physiologically relevant BG18 precursor frequencies we adoptively transferred 1x10^5^ total CD45.2^+^ BG18 IgH^+/WT^ B cells into CD45.1^+^ WT acceptor mice, and then immunized animals with secreted N332-GT2 replicon, TM N332-GT2 replicon, or recombinant N332-GT2 trimer protein combined with a saponin adjuvant (Fig. 5A). We then recovered B cells from draining lymph nodes at 2, 4, or 6 weeks post immunization for analysis. Both immunogens recruited CD45.2^+^ B cells to GCs, and these cells remained over all examined time-points (Fig. 5B–F). The response to the replicon encoding secreted immunogen was not statistically distinguishable from the protein immunogen at any timepoint. However, mice immunized with the transmembrane immunogen showed a significantly higher fraction of CD45.2^+^ B cells in GCs at the early, 14 day post-injection time point, after which the response trended back toward those to the other immunogens (Fig. 5F). Thus, replicons encoding both soluble and transmembrane forms of N332-GT2 could stimulate B cells bearing partially humanized BCRs, and the transmembrane form enhanced early GC participation by BG18 precursors. In addition to analyzing the GC compartment, we evaluated plasma cell responses (Supplementary Fig. S6A) and found a significantly higher percentage of adoptively transferred BG18 CD45.2^+^ B cells within the plasma cell compartment at week 2 post-immunization in mice that received the TM N332-GT2 replicon, compared to those immunized with the secreted N332-GT2 replicon or the SMNP-adjuvanted protein trimer (Supplementary Fig. S6B). Among the CD45.2^+^ plasma cells, the proportion of epitope-specific (N332-GT2^++^) cells was also significantly higher in animals immunized with the TM replicon-encoded trimer compared to those receiving the soluble replicon-encoded trimer (Supplementary Fig. S6C).

For further investigation whether these immunization strategies can drive the affinity maturation of the adoptively transferred BG18 B cells differently, we sorted CD45.2^+^ N332-GT2^+^ B cells week 4 and week 6 post immunization and sequenced them. Analysis of paired BCR sequences showed a preference of two murine light chains (LCs) in responding BG18 B cells, as observed previously for this immunogen^14^ (Supplementary Fig. S7). The selection of these LCs remains stable over time in week 6 post immunization (Supplementary Fig. S8). Analysis of heavy chain (HC) mutations revealed that all 3 forms of immunization with N332-GT2 resulted in a similar pattern of mutations in the HC. At week 6 post immunization, complementary determining regions from the knock-in BCRs showed only a few qualitative differences. Together these findings indicate that N332-GT2 delivered in transmembrane and soluble forms have similar immunogenicity.

To gain further insights into the output immune response, we examined serum IgG responses elicited in the adoptive transfer model. In contrast to the responses elicited in wild type mice, animals adoptively transferred with BG18 precursor B cells and vaccinated with TM N332-GT2 replicon had substantially higher trimer-specific IgG titers in serum compared to animals vaccinated with the secreted N332-GT2 replicon at all time points; high trimer-specific IgG titers were also measured for animals receiving the trimer protein/saponin immunization (Fig. 5G, Supplementary Fig. S9A). To assess the proportion of this antibody response that reflected an “on target” response to the BG18 epitope at the trimer apex (vs. competitor endogenous B cell responses against other epitopes on the trimer), we also carried out ELISA analysis of responses to an epitope knockout form of the trimer (GT2-KO); this antigen bears mutations in the BG18 binding site that prevent recognition by BG18 IgH^+/WT^ B cells. This analysis showed that the recombinant protein immunization elicited a much greater response to non-BG18 epitopes compared to the TM trimer replicon (Supplementary Fig. S9B). To quantify the proportion of the antibody response directed “on target” to the BG18 epitope, we calculated a ΔAUC, subtracting the ELISA OD vs. serum dilution area-under-the-curve (AUC) for the N332-GT2KO response from the total N332-GT2 ELISA AUC.^14,18,30,31^ TM N332-GT2 replicon immunization resulted in much higher epitope-directed response (ΔAUC) compared with both secreted N332-GT2 replicon and adjuvanted N332-GT2 trimer protein. Notably, the low levels of on-target responses were comparable between the recombinant protein and the replicon-expressed secreted trimer (Fig. 5H, Supplementary Fig. S9C). Thus, the transmembrane encoding replicon elicited a much greater epitope-specific serum IgG response compared to both forms of soluble immunogen delivery in the BG18 adoptive transfer model.

## DISCUSSION

Unlike traditional protein subunit vaccines, mRNA vaccines enable the expression of antigens not only in a soluble/secreted form but also in a transmembrane form, resembling native envelope spikes expressed in a viral membrane. Transmembrane immunogens are of particular interest in the setting of HIV vaccine design, as the use of a TM form is thought to minimize or eliminate access to immunodominant non-protective epitopes at the base of the Env trimer.^14,20,32,33^ Here, we studied the impact of immunogen format on immune responses to self-replicating RNA, which allows for the amplification of antigen-encoding transcripts within cells and sustained antigen expression over time. Specifically, we used a germline-targeting HIV Env trimer (N332-GT2) engineered to engage precursors of broadly neutralizing antibodies (bnAbs) targeting the glycan supersite at the base of the V3 loop. Our earlier study^17^ showed that germline targeting immunogens encoded from replicons elicited strong humoral responses; however, that work did not include a comparison of secreted and TM immunogens and did not employ a defined model system for tracking precursor B cell recruitment. We found that both secreted and transmembrane (TM) forms of replicon-encoded trimers were highly immunogenic, generating comparable antibody and T cell responses. Interestingly, the TM form induced smaller germinal center (GC) responses but with a higher concentration of trimer-binding B cells. In a B cell receptor knock-in model, the TM form also recruited more epitope-specific antibodies and broadly neutralizing precursor B cells compared to secreted trimer or protein trimer with a saponin adjuvant, and elicited higher epitope-specific antibody responses in the blood.

Immunization with RNA encoding non-stabilized HIV Env trimers has been shown to elicit high levels of antibodies against irrelevant epitopes, which may reflect responses to poorly folded Env structures.^32^ We thus examined the antigenicity profile and glycosylation of replicon-expressed trimers, to determine whether the replication of replicon RNA and antigen-expressing subgenomes of the replicon impact antigen expression fidelity, folding, or glycan processing. Probing of both secreted and TM N332-GT2 trimer with panels of structure-sensitive antibodies suggested that both immunogen formats were expressed as well-folded trimers, with appropriate display of broadly neutralizing epitopes and limited exposure of non-neutralizing sites. Further, for the secreted trimer which could be isolated in sufficient amounts for glycan analysis, we found that replicon-expressed trimer was produced with glycosylation patterns largely mirroring recombinant protein produced from transfected DNA, with the exception that replicon expression led to increased prevalence of complex glycans at some sites on the Env.

Examining innate immune activation and the immunogenicity of these two immunogen formats in wild-type mice, we found that TM Env trimers expressed by replicons triggered higher early levels of inflammatory cytokines and chemokines in the draining lymph node. This early augmented cytokine response correlated with a higher frequency of antigen-binding B cells in GCs primed by the TM immunogen compared to the secreted Env trimer format, though further work will be needed to determine if these outcomes are causally linked. In other measures, TM and secreted Env replicons elicited comparably strong T cell and serum antibody responses. Interestingly, previous work by Komori et al. examined membrane-bound and secreted forms of the receptor-binding domain (RBD) of SARS-CoV-2 spike protein using replicon RNA.^34^ In their studies they observed higher antibody titers with the TM form compared to the secreted form. This discrepancy may reflect the inherently low immunogenicity of SARS-CoV-2 RBD, and its small size (less than 50 kDa), which will lead to inefficient trafficking of the secreted antigen to draining lymph nodes.^35^ N332-GT2 replicon immunization elicited all four antibody isotypes regardless of the antigen form, but titers of IgG3 antibody were greater in mice immunized with TM-trimer replicon. Different subclasses of antibodies have varying affinities for Fc receptors and therefore possess distinct functional attributes. In particular, IgG3 antibodies have the ability to activate complement, demonstrate a high affinity for FcγRI, FcγRII, FcγRIIa, and FcγRIII receptors. They also possess the longest and most flexible hinge region among the IgG subclasses.^36–38^ Previous studies have reported the role of IgG3 antibodies in the immune-mediated control of certain pathogens for example in induction of long-term protection against malaria^39^ and Chikungunya virus^40^. In the setting of HIV, HIV-1–specific IgG3 has been shown to be correlated with decreased risk of HIV-1 infection in human clinical trials,^41^ and IgG3 bnAbs have been found to exhibit improved binding to FcγRIIa which correlated with enhanced phagocytosis, and increased antibody-dependent cellular cytotoxicity.^42^

Our findings using the BG18 BCR knock-in adoptive transfer model highlight the potential advantages of using TM immunogens encoded by replicon RNA in the context of germline targeting strategies for HIV vaccines. We recently reported that mRNA vaccines encoding a TM stabilized Env trimer enabled neutralizing-precursor B cells with a lower affinity to be recruited to germinal centers, compared to immunization with a soluble trimer protein and adjuvant.^14^ In the BCR knock-in adoptive transfer model of germline targeting immunization, the TM trimer format recruited a higher frequency of bnAb precursor B cells in the early stages of the GC response, and greatly increased the serum antibody response recognizing the target epitope on Env. The serum response also coincided with generation of higher CD45.2^+^ BG18 plasma cells in response to TM Env immunogen. We hypothesize that these findings may be a result of the transmembrane form of the antigen lowering competitor B cell responses against irrelevant base epitopes of the trimer and/or greater stability of the TM immunogen leading to more effective presentation of the intact BG18 epitope *in vivo*. Another possible mechanism is that membrane anchoring enhances B cell activation via mechanosensitive calcium channels such as Piezo1, which are engaged during antigen extraction.^43^

However, given that similar patterns of mutations were observed in the heavy chain across immunization groups, additional boosting with immunogens bearing an increasing number of native-Env features will be necessary to evaluate whether the TM format better supports the maturation of bnAb precursors toward broad neutralization.^31^ Notably, serum antibody responses targeting the BG18 epitope did not differ between the TM and soluble trimer replicons in wild-type mice, underscoring the critical role of precursor frequency in shaping the early antibody response. Our study builds upon and extends prior work by Aldon et al., who demonstrated that TM replicon immunogens enhanced early IgG responses and IgG2a class switching compared to soluble trimers, but used a non-germline-targeting Env and did not assess precursor B cell recruitment. In contrast, our immunogen was specifically designed for germline targeting, and we directly tracked rare bnAb precursor B cells using the BG18 BCR knock-in adoptive transfer model.^15^

In summary, these studies have identified several elements of the immune response that are altered simply by changing the physical form of an encoded HIV Env immunogen, when delivered as a self-amplifying RNA. Notably, antigen-specific germinal center responses and lymph node cytokine/chemokine induction were altered simply by changing the form of the Env immunogen encoded by the replicon. Further optimization of replicon-encoded immunogen design may enable these differences to be exploited to further enhance priming of bnAb precursor B cells for HIV and could be useful for promoting protective immune responses against other viral pathogens.

## MATERIALS AND METHODS

### Replicon synthesis

Replicon RNA was produced by *in vitro* transcription as described previously with slight modifications.^44^ In brief, DNA plasmids containing the replicon, flanked by a T7 promoter on the 5′ end and encoding a poly(A) sequence followed immediately by MluI restriction sites on the 3’ end, was amplified in *E. coli* and purified using the Plasmid QIAprep spin midi kit (QIAGEN). The amplified DNA was linearized with MluI restriction enzyme (New England Biolabs) and was then transcribed using HiScribe T7 High Yield RNA Synthesis Kit (New England Biolabs) and purified using PureLink RNA Mini columns (Thermo Fisher). The transcript was then post transcriptionally capped with methylation (ScriptCap Cap 1 Capping System, CellScript). After capping, the replicon RNA was purified a final time using PureLink RNA Mini columns (Thermo Fisher). The quality of the resulting replicons was assessed using UV-Vis spectrophotometry and gel electrophoresis.

### Formulation and characterization of replicon-encapsulating LNPs

To encapsulate replicons in LNPs, lipids including N1,N3,N5-tris(3-(didodecylamino)propyl)benzene-1,3,5-tricarboxamide (TT3, synthesized as described previously^45^), 1,2-dioleoyl-sn-glycero-3-phosphoethanolamine (DOPE, Avanti Polar Lipids), cholesterol (Chol, Avanti Polar Lipids), and 1,2-dimyristoyl-sn-glycero-3-phosphoethanolamine-N-[methoxy(polyethylene glycol)-2000 (DMPE-PEG, Avanti Polar Lipids) were dissolved in ethanol at a 22:33:44:1 TT3/DOPE/Chol/DMPE-PEG molar ratio and at a total concentration of 5.8 μg/mL. RNA was dissolved in 100 mM citrate buffer pH 3 at 60 μg/mL, and then combined with the lipid solution at an N:P ratio of 2:1 (N, number of nitrogens on TT3 ionizable lipid; P, number of phosphate groups on RNA) using an Ignite microfluidic mixing system (Precision Nanosystems) at a flow rate of 12 ml/min and an ethanol:aqueous volume ratio of 1:2. The resulting replicon-loaded LNPs were then dialyzed against PBS using a 20K MWCO Slide-A-Lyzer mini dialysis device (Thermo Fisher). Particles were characterized for size and zeta potential using a Zetasizer Nano ZSP equipment (Malvern) and imaged by cryoelectron microscopy using a JEOL 2100F transmission electron microscope operating at an acceleration voltage of 200 kV. The replicon encapsulation efficiency was quantified using a RiboGreen RNA assay kit (Thermo Fisher) following the manufacturer’s instructions. Briefly, rep-LNPs were resuspended in Tris-EDTA without or with Triton X-100 (0.5% v/v) to disrupt the particles. RNA encapsulation efficiency was defined as the percentage of replicon encapsulated in LNPs relative to total replicon.

### Synthesis of N332-GT2 protein immunogen and saponin adjuvant

N332-GT2 trimer protein bearing a C-terminal 6x His-tag was produced in 293F cells using the Expi293 expression system (Thermo Fisher, catalog no. A14524) following the manufacturer’s instructions for culturing and transfection. Following expression, the His-tagged protein was purified from the supernatant of 293F cells by Ni-NTA affinity chromatography using a 5-ml HisTrap HP column on an ÄKTA pure 25-L FPLC system (Cytiva Life Sciences, catalog no. 29018224). The column was equilibrated in PBS running buffer and 293F supernatant was applied at 5 ml/min. After washing the column with PBS to remove unbound protein, the his-tagged trimer was eluted with 0.5M imidazole in PBS buffer (pH 7.4) and exchanged into PBS using Amicon Ultra-4 centrifugal filtration systems (Millipore). SMNP saponin adjuvant was produced as previously described.^46^

### Antigenicity characterization of and replicon encoded transmembrane and secreted trimer

Antigenicity profiles of replicon-encoded transmembrane and secreted trimers were evaluated *in vitro* by flow cytometry and ELISA, respectively. The following neutralizing and non-neutralizing human monoclonal antibodies were used: PG9, PGT121, 3074, 35O22, A32, PCT64, PGT145, PGT151, 4025, BG18, and RM19R. The RM19R, PCT64, PGT145, PGT151, 4025, and BG18 antibodies were produced in HEK293F cells (Thermo Fisher) and purified using Protein A affinity chromatography (Cytiva) following established protocols.^47^ All other antibodies were sourced from BEI Resources (NIH, NIAID).

For transmembrane trimer, C2C12 mouse skeletal muscle cells (ATCC) were transfected with replicons by electroporation using a Neon transfection system (1400V, 20 ms, 1 pulse) following the manufacturer’s instructions. At 24 h post transfection, cells were harvested by trypsinization, washed by PBS and incubated with Zombie Aqua fixable viability dye for 15 min at 25°C following which they were washed with flow cytometry buffer (2% FBS, 3 mM EDTA in PBS). Cells were then incubated with flow cytometry buffer containing 2 μg/mL of the primary monoclonal antibodies for 20 min at 25°C, washed, and stained with PE-Cy5 conjugated anti-human IgG secondary antibody (BD Biosciences) at a 1:5 dilution in flow cytometry buffer for 20 min at 25°C. Stained cells were analyzed on a Becton Dickinson Symphony flow cytometer and data were processed using FlowJo v10 software.

A sandwich ELISA assay was used for antigenicity characterization of the replicon-encoded secreted trimer. To obtain enough protein for the assay, BHK-21 cells were chosen for transfection as they are known to be defective in the IFN pathway allowing for high protein yield. BHK-21 cells were electroporated with secreted trimer-encoding replicon and the cell supernatant was collected 24 hours later. Concentration of trimer in the supernatants was measured by ELISA using human and mouse VRC01 antibodies as coating and detection antibodies, respectively. High-binding half-area 96-well Costar plates (Corning) were coated with supernatant diluted in PBS to 2 μg/ml trimer concentration, and then plates were blocked with diluent buffer (2% BSA in PBS). After a wash step, serial dilutions of the indicated monoclonal antibodies were added to the plate and incubated for 2 h at 25°C. Plates were then washed and incubated with a goat HRP (horseradish peroxidase)-conjugated anti-human IgG (Jackson ImmunoResearch) in diluent buffer for 1 h at 25°C. After a final wash, plates were developed with 3,3′,5,5′-tetramethylbenzidine (TMB) substrate (1-step Ultra TMB-ELISA Substrate Solution Thermo Fisher) for 10 min following which the reaction was stopped with 2N H_2_SO_4_ and absorbance readings at 450 nm and a reference wavelength of 540 nm were read using a Tecan Infinite 200PRO plate reader.

### Animals and immunizations

Female BALB/c mice (strain: 000651, 6–8 weeks old) or male (8–10 weeks old) CD45.1^+/+^ mice (B6.SJL-Ptprc^a^ Pepc^b^/BoyJ) were purchased from the Jackson Laboratory. Mice B cells expressing a humanized BG18 BCR were generated as before.^48^ Mice were housed in a 12-hr light-dark cycle with ad libitum access to water and standard laboratory chow. All animal procedures were conducted in accordance with protocols approved by the Institutional Animal Care and Use Committee (IACUC) and conducted in accordance with the regulations of the Association for Assessment and Accreditation of Laboratory Animal Care International (AALAC). B cells from donor BG18 IgH^+/WT^ were isolated using a Pan B cell isolation kit II (Miltenyi Biotec) and were adoptively transferred through tail vein injection into acceptor B6.SJL-Ptprca Pepcb/BoyJ (CD45.1 mice) at the indicated frequencies. Mice were immunized 24 hours post adoptive transfer. Groups of mice were injected bilaterally in the gastrocnemius muscles with LNP-encapsulated replicons at 2 μg total replicon dose (1 μg per injection site) or subcutaneously at the base of tail with 10 μg protein trimer antigen and 5 μg SMNP total, administered as two separate injections with the dose split equally between the two sites.

### ELISA measurement of antibody titers

Anti-trimer serum Ig titers were assessed by ELISA. Half-area high-binding 96-well plates (Corning Life Sciences) were coated overnight at 4°C with 1 μg/ml (50 μl/well) of streptavidin in PBS. Coated plates were blocked with 2% BSA in PBS, and incubated overnight at 4°C. Plates were then washed and incubated with 1 μg/ml biotinylated trimer in block buffer (2% BSA in PBS) at for 2 h at 25°C, following which serial dilutions of serum samples (1:20 to 1:160 followed by serial 4-fold dilution) were added to the plates. After 2 h incubation at 25°C, plates were washed with incubated with HRP-conjugated anti-mouse IgG, IgG1, IgG2a, IgG2b, and IgG3 (Bio-Rad) diluted 1:5,000 in block buffer. The reaction mixture was incubated at 25°C for 30 min. At the end of the incubation period, the plates were washed and treated with 50 μL TMB substrate. The reaction was stopped after 10 min with a 1M H_2_SO_4_ solution. The plates were read on a Tecan Infinite 200PRO plate reader at 450 nm with background correction at 540 nm. The serum titer was determined as the logarithm of the reciprocal of the serum dilution that resulted in absorbance readings exceeding 0.1 OD unit above background.

For measuring serum titer in BG18 adoptive transfer experiments, plates were coated overnight at 4°C with 1 μg/ml (50μl/well) of anti-His antibody in PBS. After overnight incubation plates were washed and coated overnight at 4°C with 1 μg/ml (50μl/well) of N332-GT2 or GT2-KO his-tagged protein. After incubation, plates were washed with PBS containing 0.01% Tween-20 (PBST) and blocked for an hour with blocking buffer PBST containing 3% BSA. Post wash, plates were incubated with serial dilutions of serum samples (1:100 and followed by serial 3-fold dilutions) overnight at 4°C. After incubation, plates were washed and incubated with alkaline phosphatase conjugated anti-mouse IgG (Jackson ImmunoResearch) diluted 1:1000 in PBST containing 0.5% BSA. The reaction mixture was incubated at 25°C for 1hour and washed. Post wash, the ELISA was developed with p-Nitrophenyl Phosphate (Sigma) and stopped after 30 min with 3N NaOH post color development. Absorbance was measured at 405nm using a Biotek plate reader (Synergy Neo2).

### Construction of antigen tetramers

To construct the tetramer of N332-GT2 trimers to identify antigen-specific B cells, an avi-tagged version was of the trimer was recombinantly constructed and purified by his-tag affinity chromatography as above. The Avi-tagged trimer was first biotinylated using a BirA biotinylation kit (Avidity, Denver, CO) following the manufacturer’s protocol. The biotinylated trimer solution was then centrifuge-filtered with a 10-kDa Amicon centrifugal filter unit to remove free biotin and exchange its buffer into PBS. On the day of staining, the biotinylated trimer was crosslinked with APC-, APC-Cy7-, BV421, and BV605-labelled streptavidin (Thermo Fisher) to form the antigen tetramer by mixing in a 5:1 trimer:streptavidin molar ratio. Tetramers were added to the cells at a concentration of 20 nM in flow cytometry buffer for staining.

### Cytokine analysis

Cytokine levels were measured in serum and dLNs at 6, 24, 48, and 96 h post immunization using a LEGENDplex antivirus response panel (BioLegend, 740622) following the manufacturer’s protocol. At each time point, blood samples were collected in serum gel tubes with clotting activator and spun down to isolate the serum. Following blood collection, mice were sacrificed, and draining lymph nodes (popliteal and iliac LNs for replicon immunizations and inguinal LNs for protein immunizations) were removed. Soluble content of LNs was extracted by smashing LNs in PBS containing 2% BSA and protease inhibitors (Thermo Fisher) followed by centrifugation to remove cells and debris. Sera and LN extracts were then flash-frozen and stored at -80 till analysis.

### T cell ELISpot

IFN-γ ELISPOT assay was used to quantify antigen-specific effector T cells. Spleens were excised and smashed through 70 μm cell strainer into single cell suspensions. Following red blood cell lysis with ACK buffer, splenocytes were resuspended in ELISpot complete media [RPMI 1640 containing 10% fetal bovine serum, penicillin-streptomycin (100 U/ml), and 1 mM sodium pyruvate] and seeded at 10^6^ cells per well in mouse IFN-γ ELISPOT plates (BD). Next, splenocytes were stimulated for a period of 20 hours in a 37 °C/5% CO_2_ incubator with a pool of overlapping peptides spanning the entire trimer protein sequence (15-mer peptides overlapping by 11 residues). Plates were then washed and incubated with biotinylated anti-IFN-γ detection antibody followed by streptavidin-HRP for 2 h and 1 h, respectively, with wash steps after each incubation period. Plates were developed with substrate BCIP/NBT chromogen (MABTECH) for 30 min following the manufacturer’s instructions, quenched with water, dried overnight, and scanned using a CTL-ImmunoSpot Plate Reader. Spot counting was performed by a blinded investigator using CTL-ImmunoSpot Software.

### Memory B cell analysis

Splenocytes were harvested and processed as described above. Splenocytes were resuspended in PBS, plated in a 96-well plate, and stained with Zombie Aqua Fixable Viability dye (BioLegend) for exclusion of dead cells. After washing and resuspension in flow cytometry buffer, cells were incubated with APC/Cy7- and BV421-labelled antigen tetramer followed by the following antibodies: B220-PE-Cy7, BioLegend, clone RA3–6B2), CD3-PerCP-Cy5 (BioLegend, clone 145–2C11), GL7-FITC (BioLegend, clone GL7), CD38-BV711 (BD Biosciences, 90 clone), IgD-APC (Invitrogen, 11–26c clone), CD138-PE (BioLegend, clone 281–2), CD73-BV605 (BioLegend, clone TY/11.8), and PDL2-BUV395 (BD Biosciences, clone TY25) . Next, cells were washed and fixed in 4 % (v/v) paraformaldehyde for 15 min at 25°C. Stained samples were analyzed on a Becton Dickinson symphony flow cytometer (BD Biosciences) and data processed using FlowJo software. After exclusion of doublets and dead cells, antigen-specific memory B cells were defined as CD3^−^ BB220^+^ GL7^−^ CD38^+^ IgD^−^ CD138^−^ PDL2^+^ CD73^+^ cells that were dually stained with BV421- and APC/Cy7-conjugated trimer.

### GC analysis

The inguinal, iliac and popliteal lymph nodes were harvested from mice at week 2, 4, 6 or 8 wks post intramuscular immunizations. To obtain single cell suspensions, the lymph nodes were filtered through a 70 μm cell strainer. The resulting cells were then subjected to staining for viability (Zombie Aqua Fixable Viability Kit, BioLegend) and against CD4 (BV711, BioLegend, clone RM4–5), B220 (PE-Cy7, BioLegend, clone RA3–6B2), CD38 (FITC, BioLegend, clone 90), CXCR5 (PE, BioLegend, clone L138D7), PD-1 (BV421, BioLegend, clone 29F.1A12), and GL7 (PerCP-Cy5.5, BioLegend, clone GL7). Antigen-specific staining was done using biotinylated trimer conjugated to either streptavidin-BV605 (BioLegend) or streptavidin-APC (BioLegend). Flow cytometry analysis was performed using a Becton Dickinson Symphony instrument, and the resulting data were processed using FlowJo software.

### Bone marrow ELISPOT

ELISpot assays were used to enumerate antibody-secreting cells (ASCs) and were run according to the manufacturer’s protocol (Mabtech). Bone marrow cells were harvested from the femur and tibia of mice by clipping the ends and flushing the bone cavity with harvest buffer. Bone marrow cells were then treated with ACK buffer to lyse red blood cells, and passed through a 70 μm strainer to obtain single-cell suspensions. Following counting, cells were resuspended at different dilutions in ELISpot complete media. Cell dilutions were then added to wells of 96-well polyvinylidene difluoride ELISpot plates (MilliporeSigma, catalog no. MSIPS4510) that had been preactivated with 35% ethanol, coated with anti-mouse IgG at 15 μg/ml in sterile PBS overnight at 4°C, and blocked with complete media for at least 30 min. Following 20 h incubation at 37°C/5% CO_2_, plates were washed and then incubated with biotinylated antigen (1 μg/ml) and biotinylated anti-mouse IgG antibody (1 μg/ml) in PBS with 0.5% BSA for total IgG and antigen-specific IgG responses, respectively, for 1 h at 25°C. Plates were washed again and incubated with 1:1000 streptavidin–alkaline phosphatase in PBS with 0.5% BSA for 1 hr at 25°C. After a final wash step, bromochloroindolyl phosphate–nitro blue tetrazolium substrate (MabTech, catalog no. 3650–10) was added to plates and developed for 30 min, quenched with water, air-dried, and quantified on an ImmunoSpot CTL analyzer.

### Glycopeptide analysis by mass spectrometry

To investigate the glycan composition of N332-GT2 trimers expressed from replicons, we electroporated replicon encoding *env* trimer into HEK-293F cells. The transfected cells were harvested after 3 days and were pelleted down, and the desired protein was purified from the supernatant using *Galanthus nivalis* lectin (GNL) beads. The purified protein was subsequently reduced and alkylated for glycan analysis using LC-MS. For determination of glycan analysis of recombinant protein, we transiently transfected plasmid DNA into mammalian cells using polyethyleneimine (PEI). After 7 days, the transfected cells were harvested, and the supernatant was purified in the same way as replicon expressed protein. To compare the glycan analysis of recombinant protein and protein expressed via replicon expression, we have used the same conditions and parameters for glycan analysis using LC-MS.

Env proteins were denatured for 1 h in 50 mM Tris/HCl, pH 8.0 containing 6 M of urea. Next, the sample was reduced and alkylated by adding 5 mM dithiothreitol (DTT) and 20 mM iodoacetamide (IAA) and incubated for 1 h in the dark, followed by a 1 h incubation with 20 mM DTT to eliminate residual IAA. The alkylated Env glycoproteins were buffer exchanged into 50 mM Tris/HCl, pH 8.0 using Vivaspin columns (3 kDa) and three aliquots were digested separately overnight using trypsin (Mass Spectrometry grade, Promega), chymotrypsin (Mass Spectrometry Grade, Promega) or alpha lytic protease (Sigma Aldrich) at a ratio of 1:30 (w/w) at 37 °C. The next day, the peptides were dried and extracted using C18 Zip-tip (MerckMilipore). Following the extraction, the peptides were dried again, re-suspended in 0.1% formic acid, and analyzed by nanoLC-ESI MS with an Ultimate 3000 HPLC (Thermo Fisher) system coupled to an Orbitrap Eclipse mass spectrometer (Thermo Fisher) using stepped higher energy collision-induced dissociation (HCD) fragmentation. Peptides were separated using an EasySpray PepMap RSLC C18 column (75 μm × 75 cm). A trapping column (PepMap 100 C18 3 μm particle size, 75 μm × 2 cm) was used in line with the LC prior to separation with the analytical column. The LC-MS gradient lasted for 280 minutes. For the first 260 minutes, a linear gradient ranging from 4% to 32% ACN in 0.1% formic acid was applied. This was followed by a 20-minute alternating gradient between 76% ACN in 0.1% formic acid and 4% ACN in 0.1% formic acid, used to ensure all the sample had eluted from the column. The flow rate was set to 300 nL/min. The spray voltage was set to 2.5 kV and the temperature of the heated capillary was set to 40 °C. The ion transfer tube temperature was set to 275 °C. The scan range was 375–1500 m/z. Stepped HCD collision energy was set to 15, 25 and 45% and the MS2 for each energy was combined. Precursor and fragment detection were performed using an Orbitrap at a resolution MS1 = 120,000. MS2 = 30,000. The AGC target for MS1 was set to standard and injection time set to auto which involves the system setting the two parameters to maximize sensitivity while maintaining cycle time. Full LC and MS methodology can be extracted from the appropriate Raw file using XCalibur FreeStyle software or upon request.

Glycopeptide fragmentation data were extracted from the raw file using Byos (Version 4.0; Protein Metrics Inc.). The glycopeptide fragmentation data were evaluated manually for each glycopeptide; the peptide was scored as true-positive when the correct b and y fragment ions were observed along with oxonium ions corresponding to the glycan identified. The MS data was searched using the Protein Metrics 305 N-glycan library with sulfated glycans added manually. The relative amounts of each glycan at each site as well as the unoccupied proportion were determined by comparing the extracted chromatographic areas for different glycotypes with an identical peptide sequence. All charge states for a single glycopeptide were summed. The precursor mass tolerance was set at 4 ppm and 10 ppm for fragments. A 1% false discovery rate (FDR) was applied. The relative amounts of each glycan at each site as well as the unoccupied proportion were determined by comparing the extracted ion chromatographic areas for different glycopeptides with an identical peptide sequence. Glycans were categorized according to the composition detected. HexNAc(2)Hex(9–3) was classified as M9 to M3. Any of these compositions that were detected with a fucose are classified as FM. HexNAc(3)Hex(5–6)Neu5Ac)(0–4) was classified as Hybrid with HexNAc(3)Hex(5–6)Fuc(1)NeuAc(0–1) classified as Fhybrid. Complex-type glycans were classified according to the number of processed antenna and fucosylation. Complex glycans are categorized as HexNAc(3)(X), HexNAc(3)(F)(X), HexNAc(4)(X), HexNAc(4)(F)(X), HexNAc(5)(X), HexNAc(5)(F)(X), HexNAc(6+)(X) and HexNAc(6+)(F)(X). Core glycans are any glycan smaller than HexNAc(2)Hex(3).

### Post-immunization analysis of BG18 B cell adoptively transferred mice

The inguinal, iliac and popliteal lymph nodes were harvested from mice at week 2, 4 or 6 wks post immunization via intramuscular and subcutaneous routes for RNA and protein vaccines, respectively. Cells were harvested by gently crushing them and passing them through a 70 μM strainer. The cells were then resuspended in PBS containing 500-fold diluted Live/Dead Blue (Thermo Scientific) and 200-fold diluted FcR Blocking reagent (Purified Rat anti-mouse CD16/CD32, BD Biosciences) for 20 mins at 4°C. After washing, antigen staining was done using biotinylated trimer conjugated to either streptavidin-Alexa488 (Biolgend) or strepatvidin-Alexa647 (BioLegend) for 30 mins at 4°C . After washing off excess antigen, cells were incubated with an antibody cocktail containing CD4, CD8, F4/80, GR-1 (APC-eFluor 780, eBioscience, clone RM4–5, 53–6.7, BM8, RB6–8C5, respectively), B220 (BUV395, BD Bioscience, clone RA3–6B2), CD38 (BUV563, BD Biosciences, clone 90), CD95 (PE-Cy7, BioLegend, clone L138D7), CD45.1 (Alexa 700, BioLegend, clone A20), CD45.2 (Brilliant Blue 700, BD Biosciences, clone 104), CD138 (BV650, BD Biosciences, clone 281–2), IgD (BV605, BD Biosciences, 11–26c.2a clone) and GL7 (PE, BioLegend, GL7 clone). For sorting, Live/Dead stain was replaced with DAPI (1000-fold dilution). The antibodies used for sorting were CD4, CD8, F4/80, GR-1 (APC-eFluor 780, eBioscience, clone RM4–5, 53–6.7, BM8, RB6–8C5, respectively), B220 (BV510, BioLegend, clone RA3–6B2), CD95 (PE-Cy7, BioLegend, clone L138D7), CD45.1 (Alexa 700, BioLegend, clone A20), CD45.2 (Brilliant Blue 700, BD Biosciences, clone 104), IgD (BV650, BioLegend, clone 11–26c.2a). Cells were washed 3 times to remove any excess antibodies.

### Cell sorting and sequencing

For sorting, cells from each individual mouse were barcoded with TotalSeq^™^-C anti-mouse Hashtag Antibodies. A total of 9 hashtags were used. Samples were sorted onto PCR tubes containing flow cytometry buffer. Sorted cells were then encapsulated and NGS libraries were prepared following the 10x Genomics Chromium Next GEM Single Cell 5’ Reagent Kits v2 protocol (10x Genomics). TapeStation Systems D5000 high sensitivity Screen Tape assay (Agilent, Santa 5 Clara, CA) was used to measure library size. After quantifying the libraries through Qubit dsDNA High Sensitivity (Invitrogen), they were pooled and were run on NextSeq 550 System (Illumina, San Diego, CA). Analysis was performed using Cell Ranger v.6 software pipeline (10x Genomics) with a customized reference database. For sorting on Week 6, single cells were sorted on 96 well plates as described before.^18^ BCR sequencing on single sorted 96 well plates were done as before.^18,31^ Following RT (reverse transcriptase) step and BCR amplification (PCR-1 and PCR-2 Nested PCR reaction), sanger sequencing was done on individual samples (Genewiz).^31^ Sequencing data was analyzed using Geneious Biologics software (Geneious) and IMGT/V-Quest.

## Supplementary Material

1

## Data Availability

All data are available upon request from authors.

## Box

Replicon RNA vaccines encoding HIV Env trimers in a transmembrane form elicit focused germinal center responses and enhance recruitment of broadly neutralizing antibody precursors compared to secreted formats. These findings highlight the immunological advantages of membrane-anchored immunogens in RNA vaccine design, with implications for HIV and other viral vaccine strategies.
