# Supplementary material for: Augmented humoral responses to HIV Env trimers delivered as transmembrane immunogens by self-replicating RNA
Source: Mol Ther. 2025 Jul 29;33(10):4858–73. doi: 10.1016/j.ymthe.2025.07.036 (PMC12425358; doi:10.1016/j.ymthe.2025.07.036)
Supplement: Document S2. Article plus supplemental information [file mmc2.pdf]

Molecular Therapy
Original Article

Augmented humoral responses to HIV Env trimers delivered as transmembrane immunogens by self-replicating RNA

Parisa Yousefpour,[1,2,3] Amrit Raj Ghosh,[2] Himanshi Chawla,[4] Rachel Yeung,[1] Justin Gregory,[1] Kristen Si,[1,12] Tanaka K. Remba,[1] Kristen A. Rodrigues,[1,2,3,5] Mariane B. Melo,[1,2,3,6] Jonathan Dye,[1] Jon M. Steichen,[3,6,7] Yuebao Zhang,[9] Yizhou Dong,[10] Max Crispin,[4] William R. Schief,[3,6,7,8] Facundo D. Batista,[2,11] and Darrell J. Irvine[1,3,6,12,13]

[1]Koch Institute for Integrative Cancer Research, Massachusetts Institute of Technology, Cambridge, MA, USA; [2]Ragon Institute of Massachusetts General Hospital, Massachusetts Institute of Technology and Harvard University, Cambridge, MA, USA; [3]Consortium for HIV/AIDS Vaccine Development, The Scripps Research Institute, La Jolla, CA 92037, USA; [4]School of Biological Sciences, University of Southampton, SO17 1BJ Southampton, UK; [5]Harvard-MIT Health Sciences and Technology Program, Institute for Medical Engineering and Science, Massachusetts Institute of Technology, Cambridge, MA 02139, USA; [6]Department of Immunology and Microbiology, The Scripps Research Institute, La Jolla, CA 92037, USA; [7]IAVI Neutralizing Antibody Center, The Scripps Research Institute, La Jolla, CA 92037, USA; [8]Moderna, Cambridge, MA 02139, USA; [9]Division of Pharmaceutics & Pharmacology, College of Pharmacy, The Ohio State University, Columbus, OH 43210, USA; [10]Icahn Genomics Institute, Precision Immunology Institute, Department of Immunology and Immunotherapy, Department of Oncological Sciences, Tisch Cancer Institute, Friedman Brain Institute, Biomedical Engineering and Imaging Institute, Icahn School of Medicine at Mount Sinai, New York, NY 10029, USA; [11]Department of Biology, Massachusetts Institute of Technology, Cambridge, MA 02139, USA; [12]Department of Biological Engineering, Massachusetts Institute of Technology, Cambridge, MA, USA; [13]Howard Hughes Medical Institute, Chevy Chase, MD, USA

mRNA vaccines have emerged as an important platform for vaccine development. Unlike protein subunit vaccines, mRNA-expressed antigens can be expressed in either secreted or transmembrane (TM) forms mimicking a viral envelope (Env) protein. Here, we investigated the impact of antigen expression format on the antigenicity profile, glycosylation, and immunogenicity of stabilized HIV Env trimer immunogens expressed from self-replicating RNA (replicon) vaccines. Replicon-encoded trimers in both forms exhibited proper folding, and replicon-expressed secreted trimers exhibited glycosylation patterns largely consistent with recombinant trimer protein, although with enrichment of complex glycans over high mannose at some sites. Both formats were highly immunogenic in mice, eliciting comparable serum antibody and T cell responses. Interestingly, the TM format initiated smaller germinal center (GC) responses, but these GCs were enriched for trimer-binding B cells compared to secreted trimer vaccines. In a B cell receptor knockin adoptive transfer model for assessing germline targeting, the replicon-encoded TM trimer elicited a greater frequency of epitope-targeting antibodies and recruited broadly neutralizing antibody precursor B cells to the GC response more efficiently compared to the replicon-encoded secreted trimer or protein trimer combined with adjuvant. These results indicate that the form of immunogen expression can impact key elements of immune responses to RNA vaccines.

INTRODUCTION

Vaccines comprising *in vitro* transcribed and capped antigen-encoding RNA encapsulated in lipid nanoparticles (LNPs) have emerged as an important platform for current and future vaccine development. The rapid development of effective vaccines for the COVID-19 pandemic demonstrated the potential and versatility of RNA, which is a platform technology enabling the design of new vaccines using genetic sequence information without requiring alterations to the production process or facilities.[1,2] Two types of RNA vaccines have established clinical efficacy against SARS-CoV-2—those based on mRNA employing modified bases to enhance RNA stability and reduce innate immune recognition[3,4] and those based on alphavirus-derived self-replicating RNAs.[5–8] Self-replicating (replicon) RNAs encode a set of non-structural proteins (nsPs) in addition to a payload gene of interest. The nsPs form an RNA-dependent RNA polymerase that can copy both the overall RNA sequence and an encoded subgenome containing the antigen payload.[6] Replicons are of great interest for RNA vaccination because they have the potential to enable much lower total doses of RNA to be used[9–12] and, concomitantly, lower doses of lipid delivery vehicle, which can increase tolerability and enable dose sparing. A recent clinical trial has also indicated potential benefits of heterologous immunization with replicons followed by non-replicating mRNA SARS-CoV-2 vaccines.[13]

Received 4 February 2025; accepted 24 July 2025;
https://doi.org/10.1016/j.ymthe.2025.07.036.

Correspondence: Darrell J. Irvine, Koch Institute for Integrative Cancer Research, Massachusetts Institute of Technology, Cambridge, MA, USA.
E-mail: djirvine@scripps.edu

 © 2025 The Author(s).
Published by Elsevier Inc. on behalf of The American Society of Gene and Cell Therapy.
This is an open access article under the CC BY license (http://creativecommons.org/licenses/by/4.0/).

In addition to different modalities of RNA, nucleic acid vaccines open up distinct antigen format possibilities in vaccine design. For example, viral envelope (Env) antigens can be expressed from RNA as secreted proteins or in a native transmembrane (TM) form, as in the approved Pfizer and Moderna COVID-19 vaccines. However, studies investigating the impact of the expression form of the immunogen (e.g., TM versus secreted antigens) on immune responses to vaccination are currently limited. In a humanized mouse model of HIV vaccination, we previously showed that immunization with mRNA encoding TM Env trimer antigen was more effective at activating low-affinity B cell precursors than immunization with the same trimer in the form of a soluble recombinant protein antigen combined with adjuvant.[14] Aldon et al. reported that a TM Env trimer immunogen elicited more rapid immunoglobulin G (IgG) binding titers and a skewing toward IgG2a class switching compared to an equivalent soluble Env trimer when delivered as replicon RNA in mice.[15] However, whether the immunogen format impacts other elements of the immune response primed by replicon vaccines such as germinal center (GC) responses and memory or plasma cell development, to our knowledge, has not been evaluated. In addition, we sought to investigate how antigen expression from rapidly self-replicating RNAs affects antigen folding and glycosylation of complex immunogens such as HIV Env trimers.

To address these questions, here, we studied the impact of the expression form on different facets of the immune response to LNP-delivered replicon RNA encoding a stabilized HIV Env germline-targeting immunogen. Using a Venezuelan equine encephalitis (VEE) virus-based replicon backbone we previously developed,[16,17] we designed replicons encoding secreted or TM forms of Env trimers. For these studies, we selected the N332-GT2 trimer. This is a germline-targeting immunogen engineered to engage B cell precursors capable of targeting a glycan supersite centered on N332 in the base of the V3 loop of Env, which is the most common target for broadly neutralizing antibodies (bnAbs) in HIV-1 infection, and is well conserved, occurring in 73% of 30,000 cross-clade Env isolates.[18,19] In addition to mutations enabling precursor B cells to bind the N332 supersite, the trimer sequence includes mutations designed to highly stabilize the overall trimer structure.[20] HIV trimers can be produced in a secreted form by expressing the Env lacking the native TM domain of Env gp160 or in a TM form by including the native Env TM domain truncated at the C terminus to optimize cell surface expression.[18,20] To compare the properties and immunogenicity of these two forms of trimer expressed by replicons, N332-GT2-secreted or TM sequences were cloned into a VEE-based replicon backbone we described previously.[16,17]

We found that both forms exhibited well-folded conformations, effectively displaying broadly neutralizing epitopes with limited exposure of non-neutralizing sites. Examining immune responses to these replicon-encoded immunogens, we found that both forms elicited strong and comparable T cell and antibody responses; however, the TM form triggered more selective GC responses, enriched with antigen-binding B cells, compared to the secreted form. Additionally, the TM format more efficiently recruited bnAb precursor B cells early in the GC response and elicited higher titers for the IgG3 antibody subclass. Thus, these studies provide further support for the use of TM immunogen designs for priming of productive immune responses against HIV.

RESULTS

Stabilized HIV Env trimer immunogens expressed from replicons have immunogenicity and glycan profiles similar to recombinant protein

We first compared the antigenicity profile of the secreted N332-GT2 trimer expressed by BHK-21 cells transfected with replicons via electroporation to that of trimers produced as traditional recombinant proteins from HEK Expi293 cells transfected with plasmid DNA encoding the same sequence via lipofection. Trimer in the supernatants collected from cells transfected with replicons or DNA was quantified by ELISA, yielding approximately 15 and 34 ng/1,000 cells for replicon- and plasmid DNA-transfected cells, respectively. Equivalent amounts of trimer from each platform were then captured on ELISA plates coated with the CD4 binding site-specific antibody VRC01 and probed for binding to serial dilutions of a panel of neutralizing and non-neutralizing monoclonal antibodies recognizing epitopes covering many different sites on the trimer. This characterization revealed comparable antibody binding profiles for secreted trimers expressed from replicons versus DNA-expressed trimer for the V1/V2 loops, interface/fusion peptide, and V3 loop epitopes (Figures 1A and 1B). The most notable differences were reduced binding of the gp120/gp41 interface bnAb 35O22, and slightly increased binding of the non-neutralizing antibody A32. Importantly, binding by the bnAb BG18, which N332-GT2 trimer is designed to bind to with high affinity, was high and equivalent for both recombinant protein and replicon-expressed trimer.

The glycan shield of Env trimers plays a major role in shaping the antibody response. Based on the promising evidence from antigenicity profiling that the replicon produces a well-folded secreted trimer, we next compared the glycan profile of secreted trimers produced by HEK cells transfected with DNA (traditional recombinant protein expression) versus HEK cells transfected with replicon. Combined liquid chromatography-mass spectrometry (LC-MS) analysis of trimer recovered from the supernatant of transfected cells[21–23] showed that the glycan profiles of replicon- and DNA-expressed N332-GT2 were overall very similar (Figure 1C). We observed a high abundance of oligomannose-type glycans at intrinsic mannose patch sites focused around the N332 supersite.[24,25] Furthermore, the trimer-associated mannose patch, focused around the N156 and N160 glycans, was conserved in replicon-expressed Env, which is an indicator of folding fidelity (Figure 1C).[26,27] These features represent native-like trimeric compositions. A few N-glycosylation sites were less occupied, including N185e, N197, and across gp41 sites. Interestingly, replicon-expressed Env presented a higher proportion

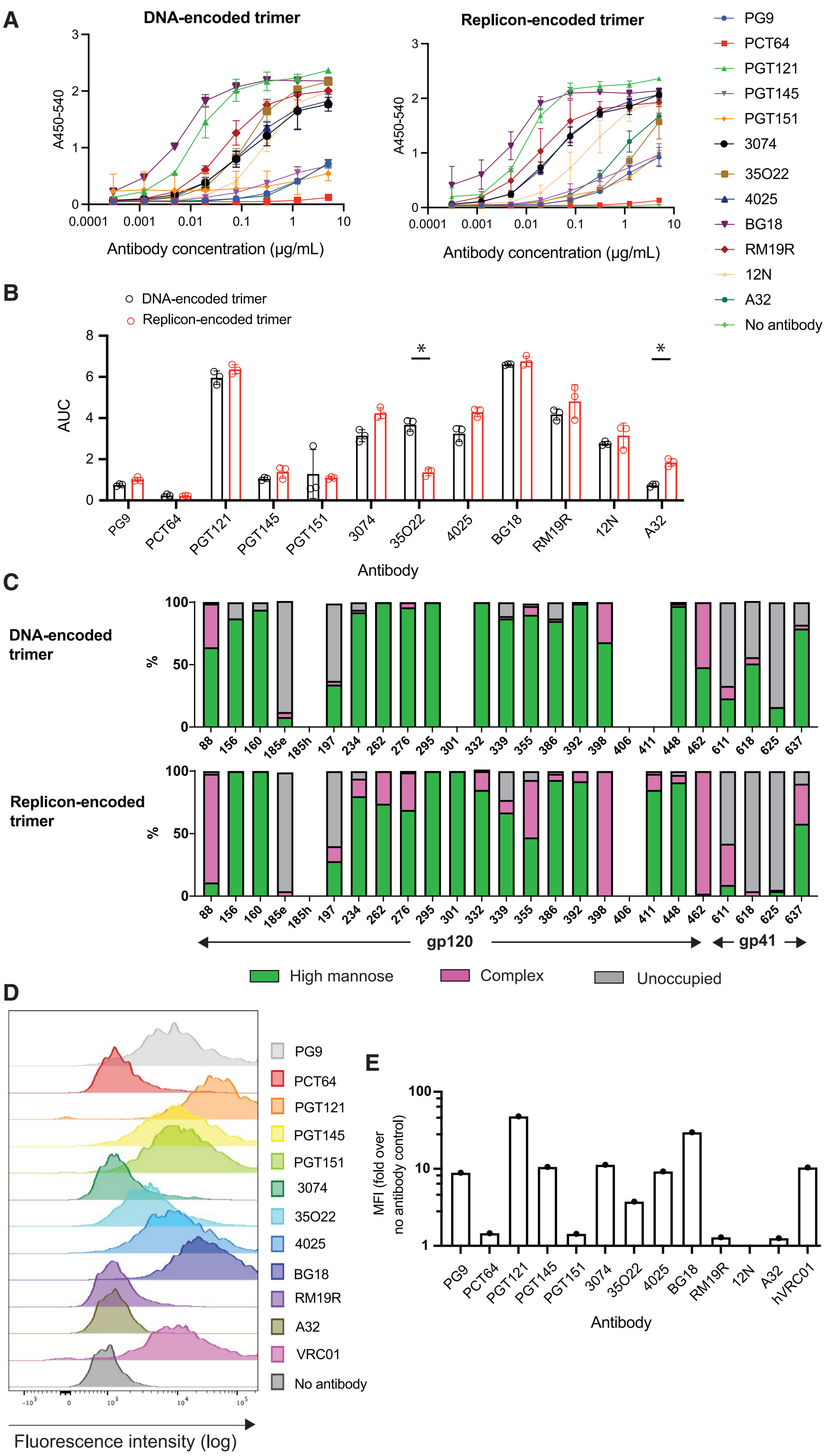

Figure 1. Characterization of replicon-encoded trimers

(A and B) Antigenicity profiling of secreted trimer. Secreted trimers were expressed from replicons (electroporated into BHK-21 cells) and from plasmid DNA (transfected into HEK Expi293 cells by lipofection). Trimers were collected from the culture supernatant, and their binding to various antibodies was assessed by ELISA. Results are presented as (A) absorbance at 450 nm with correction at 540 nm (A450-540) and (B) area under the curve (AUC). (C) Site-specific compositions of N-linked glycans located on the secreted trimer encoded from replicon or plasmid DNA in HEK Expi293 cells. Data could not be obtained for listed sites where no bar graph is displayed. (D and E) Antigenicity profiling of transmembrane (TM) trimers. Shown are (D) flow cytometry plots and (E) median fluorescence intensity (MFI) of C2C12 cells transfected with replicon by electroporation and expressing the TM trimer, following staining with antibodies targeting different epitopes on the trimer. Data are shown as means ± standard deviations. Statistical significance was determined by Student's t test.

of complex-type glycans than plasmid DNA-expressed Env at a few sites, including N88, N355, N398, and N462 (Figure 1C). Altogether, the antigenic profile and glycan composition of replicon-expressed and recombinant DNA-expressed N332-GT2 were largely similar, suggesting that replicon expression did not dramatically alter trimer folding or glycan processing.

We also carried out antigenic profiling of TM N332-GT2 by surface staining replicon-electroporated murine myoblast C2C12 cells (chosen to model intramuscular [i.m.] delivery, a preferred route for mRNA vaccines[1,28]) with the panel of neutralizing and non-neutralizing monoclonal antibodies for analysis by flow cytometry. Similar to secreted trimer, the membrane-bound trimer exhibited strong binding to antibodies targeting bnAb epitopes, including BG18, VRC01, and trimer-specific quarternary epitopes (PGT121 and PGT145; Figures 1D and 1E). By contrast, the TM trimer exhibited low binding to the non-neutralizing gp120 antibody A32 and the trimer base-specific antibody RM19R (Figures 1D and 1E). These data suggest the replicon-expressed TM trimer is also folded properly and presents expected neutralizing sites.

TM trimer replicons elicit a greater proportion of antigen-specific GC B cells but a smaller overall GC than secreted trimer immunization

To assess immune responses triggered by secreted versus TM trimer immunization, we encapsulated replicons encoding each construct form in LNPs, using a formulation we previously reported for effective replicon delivery in mice.[17] After encapsulation and buffer exchange into PBS, the LNPs exhibited an average median diameter of ∼70–100 nm across batches, based on the number distribution from dynamic light scattering and corroborated by cryo-transmission electron microscopy, with a mean zeta potential of approximately −7 mV and RNA encapsulation efficiency of above 95% (Figures S1A–S1E). These characteristics were reproducibly maintained across independent batches of replicons/LNPs, with no significant differences observed either across batches or between LNPs encapsulating TM trimer versus secreted trimer replicon constructs. We first characterized the development of antigen-specific B cell responses and GC responses by flow cytometry (Figure S2A). BALB/c mice were immunized once i.m. with 2 μg replicons encoding secreted or TM trimer, and draining lymph nodes (dLNs) were analyzed over time. Notably, the use of replicons encoding the secreted form of the immunogen led to substantially higher numbers of T follicular helper (Tfh) and total GC B cells at 2 weeks post-immunization (Figures 2A–2C and S3A–S3E). GCs contracted substantially by 4 weeks in all groups. Although the TM trimer-encoding replicon elicited much smaller total GC responses, when we stained GC B cells with fluorescent antigen tetramers, we found that TM trimer replicons elicited a substantially greater frequency of antigen-binding GC B cells, which persisted through week 4 post-immunization (Figures 2D–2F). The combination of these effects led the total antigen-binding GC B cell response over time to be equivalent for the two immunogen formats (Figure 2G). Outside the GCs, neither the secreted nor the TM trimer encoded by replicons led to a significant increase in the number of antigen-binding B cells compared to naive control groups, indicating that replicon immunization elicited little or no extrafollicular B cell response (Figure S4). We hypothesize that more antigen arrives at the dLN for replicon-generated secreted trimer, but that the TM trimer may be more stable and provide more intact antigen to drive the GC B cell response.

Replicons encoding TM trimer elicit elevated inflammatory cytokine and chemokine production in dLNs compared to secreted trimer replicons

To shed light on the differential GC responses observed for replicon immunizations with the two immunogen formats, we examined cytokine and chemokine (CK) expression in the dLNs and blood over time. This analysis revealed two waves of cytokine/CK responses in the dLN—short-lived responses that were elevated at 6 and 24 h but largely subsided by 48 h (e.g., tumor necrosis factor α, chemokine ligand 2 [CCL2], interferon- β [IFN-β]), and more sustained responses that continued to be elevated above baseline through 96 h (e.g., IFN-γ, CXCL10, CXCL1, interleukin-10 [IL-10]; Figure 3A). Quantitatively, immunization with the TM trimer replicon elicited statistically significantly higher levels of several cytokines/CKs, including IFN-γ, IL-12, IFN-β, IL-6, and CCL2 (Figure 3B). We also analyzed cytokine/CK levels in the blood and found that most inflammatory protein levels there tracked responses in the dLN, although the levels of most of the proteins measured were returning to baseline by 48 h (Figures 3C and 3D). Type I IFNs were notably upregulated early after immunization (Figures 3C and 3D). Strikingly, these data indicate that simply changing the format of the encoded immunogen can alter the inflammatory environment established in the dLN following replicon immunization.

Both secreted and TM trimer immunogen replicon immunizations prime strong T cell and antibody responses in mice

We next examined several outputs of the humoral and cellular responses to vaccination over time. At 8 weeks following a single immunization with LNP-replicons containing 2 μg replicon RNA, re-stimulation of T cells from the spleens of mice revealed comparable induction of trimer-specific memory T cell responses for both TM and secreted N332-GT2 trimer (Figure 4A). LNP-replicon immunization also elicited high titers of serum IgG against the trimer in both immunogen formats that plateaued at approximately 8 weeks post-immunization (Figure 4B). At lower doses of the replicon, a lower titer was observed, but it increased over time (Figure S5A). These robust responses are consistent with the properties of the glycan-unmasked germline-targeting immunogens, which differ substantially from native-like Env trimers that are heavily glycosylated with a "glycan shield" that limits antigen accessibility and immunogenicity.[18,29] We also tested a non-secreted intracellular form of the trimer, and consistent with

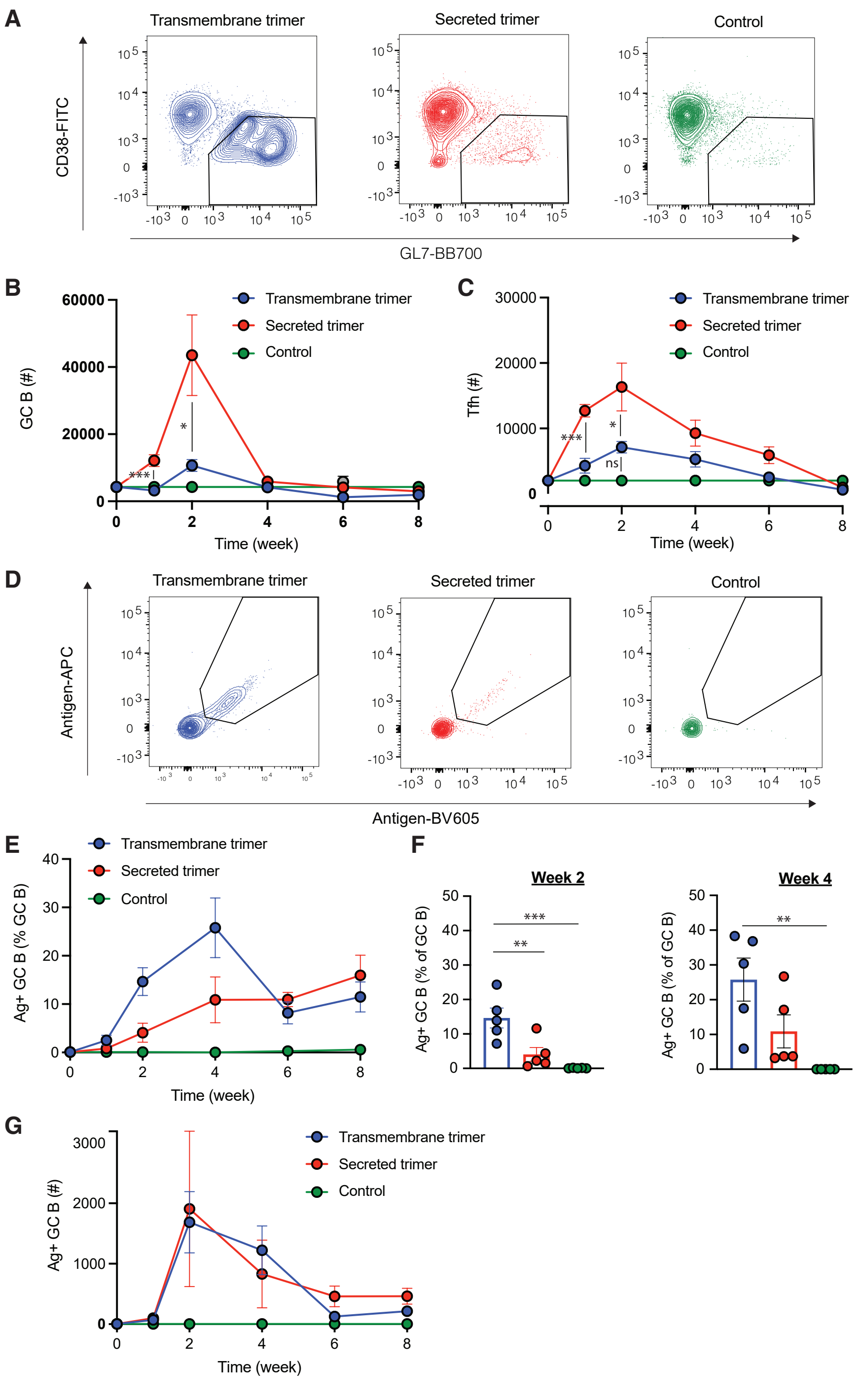

Figure 2. Secreted trimer replicons elicit larger total GC responses, but TM trimer replicons promote a greater frequency of trimer-binding GC B cells

Total GC B cell, antigen-specific GC B cell, and Tfh populations were assessed in draining lymph nodes (dLNs) at various time points after a single immunization of BALB/c mice with LNP-replicons encoding the TM or secreted trimer. (A) Representative flow cytometry contour plots showing GC B cell staining gated on B cells from mice with indicated treatments at week 2 post-immunization. (B and C) Graphs show the numbers of $GL7^{+}CD38^{+}$GC B cells (B) and $CXCR5^{+}PD\text{-}1^{+}$ Tfh cells (C) over time in mice treated with LNP-replicons. (D–G) Kinetics of antigen-specific GC B cell responses in dLNs. Shown are (D) the representative flow cytometry contour plots showing antigen staining gated on GC B cells from mice with indicated treatments at week 2 post-immunization, (E) the percentage of antigen-specific GC B cells out of the total B cells over time, (F) the percentage of antigen-specific GC B cells at weeks 2 and 4 post-immunization, and (G) the number of antigen-specific GC B cells over time. Statistical significance was assessed by two-way analysis of variance (ANOVA) followed by Tukey's post hoc test. Data are shown as mean ± SEM; **p* < 0.05; ***p* < 0.01; ****p* < 0.001.

expectations, this replicon resulted in negligible serum antibody responses, confirming that antigen exposure via secretion or membrane display is required for immunogenicity (Figure S5B). To evaluate the specificity of the antibody response toward the BG18 epitope—a bnAb site of interest—we performed ELISAs using an epitope knockout variant (N332-GT2KO) and calculated ΔAUC (change in area under the curve) values by subtracting the binding AUC of the KO trimer from that of the wild-type (WT) N332-GT2 trimer. This analysis revealed no significant difference in BG18 epitope-directed antibody responses between the TM and secreted trimer replicons (Figure 4C). Both TM and secreted trimer replicons induced class switching to all four subclasses of IgG, with TM trimer replicon eliciting a stronger IgG3 response and a trend toward stronger induction of IgG2a (Figure 4D).

We next assessed the development of long-lived plasma cells in the bone marrow. ELISpot analysis showed that both secreted and TM trimer immunogen formats led to the induction of significant trimer-specific antibody-secreting cells (ASCs) in the bone marrow at 10 weeks post-immunization, with a trend toward higher responses for the TM trimer immunogen (Figure 4E). By contrast, antigen tetramer staining revealed comparable antigen-specific memory B cell development at 4 weeks post-immunization, but memory cells further expanded by week 8 for the secreted trimer format (Figures 4F and S2B). Thus, both TM and secreted trimer replicons primed strong T cell and antibody responses, with some differences in the long-term output of plasma cellversus memory B cell responses.

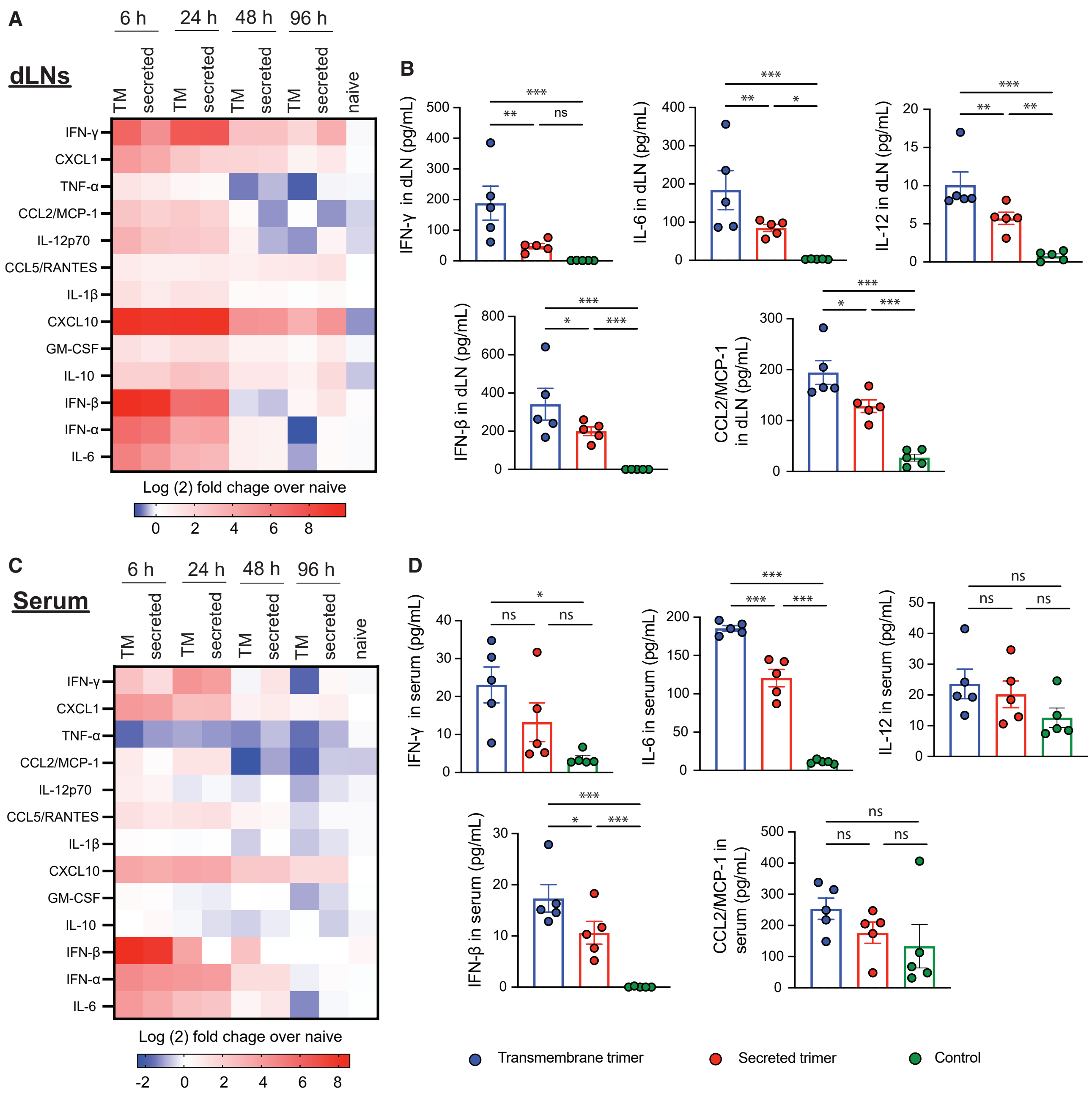

Figure 3. Replicons encoding TM trimer immunogen elicit amplified cytokines responses in dLNs and blood

BALB/c mice were immunized with LNP-replicon intramuscularly (i.m.). dLNs and serum samples were harvested at 6, 24, 48, and 96 h post-immunization, processed, and their cytokine content was measured by LEGENDplex antiviral response assay. (A) Heatmap showing the cytokine expression levels, normalized to the corresponding levels in untreated animals in dLNs, in response to vaccination with LNP-replicons encoding the TM and secreted form of the trimer. (B) Concentrations of selected cytokines in dLNs at 6 h post-vaccination. (C) Heatmap showing cytokine expression levels, normalized to the corresponding levels in untreated animals in serum, in response to vaccination. (D) Concentration of selected cytokines in serum at 6 h post-vaccination. Individual data points corresponding to each animal are plotted alongside means ± SEMs (n = 5 animals/group). Statistical significance was assessed by two-way ANOVA, followed by Tukey's post hoc test; *$p < 0.05$; **$p < 0.01$; ***$p < 0.001$.

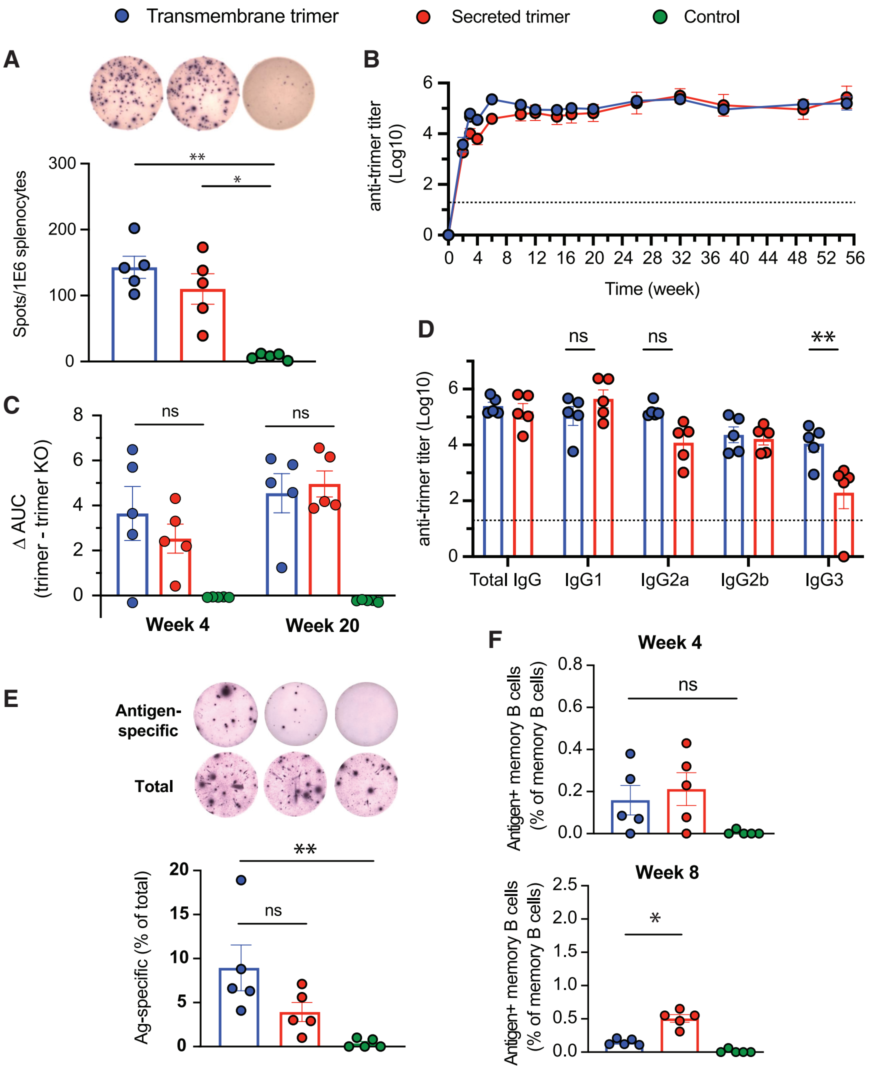

Figure 4. Cellular and humoral immune responses to vaccination

BALB/c mice were immunized with a single dose of LNP-replicon.

(A) Antigen-specific T cell responses. Splenocytes were collected at week 8 post-immunization, and T cell responses to TM and secreted N332-GT2 antigen encoded from replicons were evaluated by IFN-γ ELISpot assay with overlapping peptides spanning the entire trimer. (B) Anti-trimer antibody titer after vaccination. Serum samples were collected longitudinally and analyzed for trimer-binding antibodies by ELISA. The dashed line indicates the background response in naive mice. (C) Epitope specificity of antibody responses, Serum samples at weeks 4 and 20 post-immunization were assessed for epitope specificity using ELISA by calculating the differences in area under the curves (AUCs) for binding to the N332-GT2 trimer compared to the N332-GT2-KO (an epitope knockout) trimer. (D) Subclasses of IgG antibodies elicited. Data are from blood samples collected at week 15 post-immunization. The dashed line indicates the background response in naive mice. (E) Induction of antibody-secreting cells in bone marrow. Cells were collected from the marrow of both femurs and tibias at week 10 post-immunization, and the frequency of antibody-secreting cells was determined by total as well as antigen-specific IgG ELISpot assays. (F) Quantification of the antigen-specific memory B cells was conducted at weeks 4 and 8 post-immunization by harvesting and processing spleen; staining and gating on relevant markers by flow cytometry followed. Data are from independent experiments. Statistical significance was assessed by two-way ANOVA, followed by Tukey's post hoc test. Data are shown as mean ± SEM; *$p < 0.05$; **$p < 0.01$.

TM trimer immunogens trigger enhanced early recruitment of bnAb precursor B cells to GCs compared to secreted trimer replicons

The N332-GT2 trimer is designed to engage precursor B cells capable of evolving their antigen receptor to produce bnAbs targeting the N332 supersite of HIV Env similar to the bnAb BG18.[18] To test how secreted versus TM forms of this germline-targeting immunogen compared for priming BG18 precursor B cells, we employed an adoptive transfer B cell receptor (BCR) knockin model we recently developed for this system: BG18^{gH} mice have the inferred germline Ig heavy chain (HC) of BG18 knocked in to the BCR locus,[23] such that ∼30%–32% of B cells from these mice express the germline BG18 HC variable regions paired with native murine light chains (LCs), producing functional BCRs capable of recapitulating BG18 bnAb maturation.[18] To establish low, physiologically relevant BG18 precursor frequencies, we adoptively transferred 1×10^5 total CD45.2$^+$ BG18 IgH$^{+/WT}$ B cells into CD45.1$^+$ WT acceptor mice and then immunized animals with secreted N332-GT2 replicon, TM N332-GT2 replicon, or recombinant N332-GT2 trimer protein combined with a saponin adjuvant (Figure 5A). We then recovered B cells from dLNs at 2, 4, or 6 weeks post-immunization for analysis. Both immunogens recruited CD45.2$^+$ B cells to GCs, and these cells remained over all examined time points (Figures 5B–5F). The response to the replicon encoding secreted immunogen was not statistically distinguishable from the protein immunogen at any time point. However, mice immunized with the TM immunogen showed a significantly higher fraction of CD45.2$^+$ B cells in GCs at the early, 14-day post-injection time point, after which the response trended back toward those to the other immunogens (Figure 5F). Thus, replicons encoding both soluble and TM forms of N332-GT2 could stimulate B cells bearing partially humanized BCRs, and the TM form enhanced early GC participation by BG18 precursors. In addition to analyzing the GC compartment, we evaluated plasma cell responses (Figure S6A) and found a significantly higher percentage

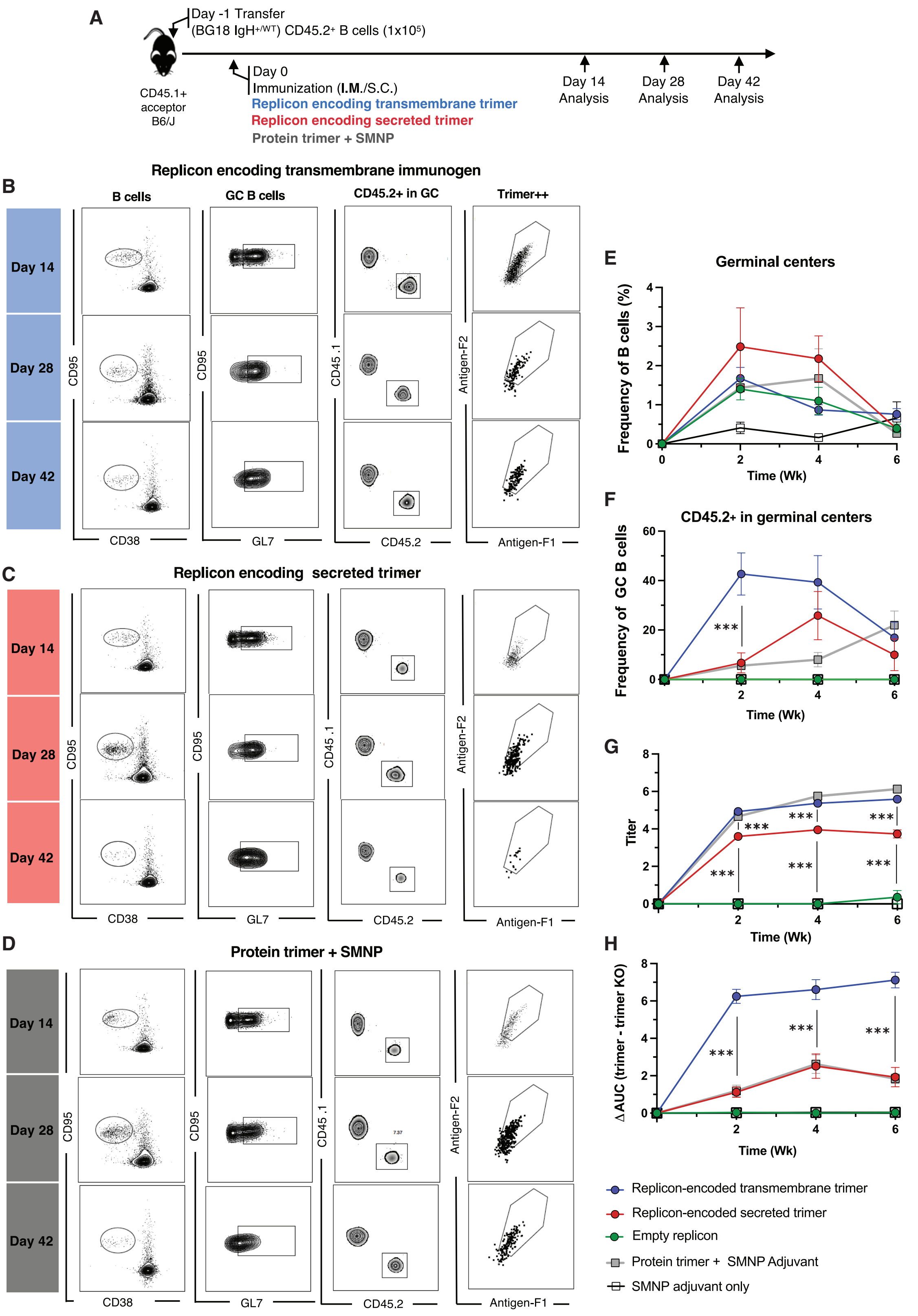

(legend on next page)

of adoptively transferred BG18 CD45.2$^+$ B cells within the plasma cell compartment at week 2 post-immunization in mice that received the TM N332-GT2 replicon, compared to those immunized with the secreted N332-GT2 replicon or the saponin/monophosphoryl lipid A nanoparticle (SMNP)-adjuvanted protein trimer (Figure S6B). Among the CD45.2$^+$ plasma cells, the proportion of epitope-specific (N332-GT2^{2+}) cells was also significantly higher in animals immunized with the TM replicon-encoded trimer compared to those receiving the soluble replicon-encoded trimer (Figure S6C).

For further investigation into whether these immunization strategies can drive the affinity maturation of the adoptively transferred BG18 B cells differently, we sorted CD45.2$^+$ N332-GT2$^+$ B cells at weeks 4 and 6 post-immunization and sequenced them. Analysis of paired BCR sequences showed a preference of two murine LCs in responding BG18 B cells, as observed previously for this immunogen[14] (Figure S7). The selection of these LCs remains stable over time in week 6 post-immunization (Figure S8). Analysis of HC mutations revealed that all three forms of immunization with N332-GT2 resulted in a similar pattern of mutations in the HC. At week 6 post-immunization, complementary determining regions from the knockin BCRs showed only a few qualitative differences. Together, these findings indicate that N332-GT2 delivered in TM and soluble forms have similar immunogenicity.

To gain further insights into the output immune response, we examined serum IgG responses elicited in the adoptive transfer model. In contrast to the responses elicited in WT mice, animals adoptively transferred with BG18 precursor B cells and vaccinated with TM N332-GT2 replicon had substantially higher trimer-specific IgG titers in serum compared to animals vaccinated with the secreted N332-GT2 replicon at all time points; high trimer-specific IgG titers were also measured for animals receiving the trimer protein/saponin immunization (Figures 5G and S9A). To assess the proportion of this antibody response that reflected an "on target" response to the BG18 epitope at the trimer apex (versus competitor endogenous B cell responses against other epitopes on the trimer), we also carried out ELISA analysis of responses to an epitope KO form of the trimer (GT2-KO); this antigen bears mutations in the BG18 binding site that prevent recognition by BG18 IgH$^{+/WT}$ B cells. This analysis showed that the recombinant protein immunization elicited a much greater response to non-BG18 epitopes compared to the TM trimer replicon (Figure S9B). To quantify the proportion of the antibody response directed "on target" to the BG18 epitope, we calculated a ΔAUC, subtracting the ELISA optical density (OD) versus serum dilution AUC for the N332-GT2KO response from the total N332-GT2 ELISA AUC.[14,18,30,31] TM N332-GT2 replicon immunization resulted in a much higher epitope-directed response (ΔAUC) compared with both secreted N332-GT2 replicon and adjuvanted N332-GT2 trimer protein. Notably, the low levels of on-target responses were comparable between the recombinant protein and the replicon-expressed secreted trimer (Figures 5H and S9C). Thus, the TM encoding replicon elicited a much greater epitope-specific serum IgG response compared to both forms of soluble immunogen delivery in the BG18 adoptive transfer model.

DISCUSSION

Unlike traditional protein subunit vaccines, mRNA vaccines enable the expression of antigens not only in a soluble/secreted form but also in a TM form, resembling native Env spikes expressed in a viral membrane. TM immunogens are of particular interest in the setting of HIV vaccine design, as the use of a TM form is thought to minimize or eliminate access to immunodominant non-protective epitopes at the base of the Env trimer.[14,20,32,33] Here, we studied the impact of immunogen format on immune responses to self-replicating RNA, which allows for the amplification of antigen-encoding transcripts within cells and sustained antigen expression over time. Specifically, we used a germline-targeting HIV Env trimer (N332-GT2) engineered to engage precursors of bnAbs targeting the glycan supersite at the base of the V3 loop. Our earlier study[17] showed that germline-targeting immunogens encoded from replicons elicited strong humoral responses; however, that work did not include a comparison of secreted and TM immunogens and did not employ a defined model system for tracking precursor B cell recruitment. We found that both secreted and TM forms of replicon-encoded trimers were highly immunogenic, generating comparable antibody and T cell responses. Interestingly, the TM form induced smaller GC responses but with a higher concentration of trimer-binding B cells. In a BCR knockin model, the TM form also recruited more epitope-specific antibodies and broadly neutralizing precursor B cells compared to secreted trimer or protein trimer with a saponin adjuvant and elicited higher epitope-specific antibody responses in the blood.

Immunization with RNA encoding non-stabilized HIV Env trimers has been shown to elicit high levels of antibodies against irrelevant

Figure 5. BG18 responses can be elicited by replicons in a precursor knockin mouse model
We adoptively transferred 1 × 10^5 B cells from BG18 IgH$^{+/WT}$ CD45.2$^+$ mice into CD45.1$^+$ recipient mice, and they were used in downstream immunization experiments. (A) Schematic of adoptive transfer (day −1) followed by immunization i.m. (day 0) and analysis (days 14, 28, and 42). (B–D) Fluorescence-activated cell sorting plots indicating ActB (activated B cells) cells gated on lymphocytes/single cells/live cells (SSL$^+$/live/dump$^-$/B220$^+$/CD38$^-$/CD95$^+$), GC B cells (CD95$^+$/GL7$^+$ ActB cells), CD45.2$^+$ cells in GCs, and antigen specificity of CD45.2$^+$ GC B cells for 14, 28, and 42 days post-immunization by the replicon encoding secreted immunogen (B), replicon encoding membrane-bound immunogen (C), and protein immunization with SMNP adjuvant (D). (E and F) Quantification of B cells in GCs (E) and CD45.2$^+$ B cells in GCs (F); each condition had 6 mice per group. (G and H) Evaluation of antibody responses: serum samples were longitudinally collected from CD45.1$^+$ recipient mice. Antibody levels were measured by ELISA and reported as titers (G), and epitope specificity of antibodies was assessed using ELISA by calculating the differences in area under the curves (AUCs) for binding to the N332-GT2 trimer compared to the N332-GT2-KO (an epitope knockout) trimer (H). Statistical significance was assessed by two-way ANOVA followed by Tukey's post hoc test. Data are shown as mean ± SEM; ***$p < 0.001$.

epitopes, which may reflect responses to poorly folded Env structures.[32] We thus examined the antigenicity profile and glycosylation of replicon-expressed trimers to determine whether the replication of replicon RNA and antigen-expressing subgenomes of the replicon impact antigen expression fidelity, folding, or glycan processing. Probing of both secreted and TM N332-GT2 trimer with panels of structure-sensitive antibodies suggested that both immunogen formats were expressed as well-folded trimers, with appropriate displays of broadly neutralizing epitopes and limited exposure of non-neutralizing sites. Furthermore, for the secreted trimer that could be isolated in sufficient amounts for glycan analysis, we found that replicon-expressed trimer was produced with glycosylation patterns largely mirroring recombinant protein produced from transfected DNA, with the exception that replicon expression led to an increased prevalence of complex glycans at some sites on the Env.

Examining innate immune activation and the immunogenicity of these two immunogen formats in WT mice, we found that TM Env trimers expressed by replicons triggered higher early levels of inflammatory cytokines and CKs in the dLN. This early augmented cytokine response correlated with a higher frequency of antigen-binding B cells in GCs primed by the TM immunogen compared to the secreted Env trimer format, although further work will be needed to determine whether these outcomes are causally linked. In other measures, TM and secreted Env replicons elicited comparably strong T cell and serum antibody responses. Interestingly, previous work by Komori et al. examined membrane-bound and -secreted forms of the receptor-binding domain (RBD) of SARS-CoV-2 spike protein using replicon RNA.[34] In their studies, they observed higher antibody titers with the TM form compared to the secreted form. This discrepancy may reflect the inherently low immunogenicity of SARS-CoV-2 RBD and its small size (less than 50 kDa), which will lead to inefficient trafficking of the secreted antigen to dLNs.[35] N332-GT2 replicon immunization elicited all four antibody isotypes regardless of the antigen form, but titers of IgG3 antibody were greater in mice immunized with TM-trimer replicon. Different subclasses of antibodies have varying affinities for Fc receptors and therefore possess distinct functional attributes. In particular, IgG3 antibodies have the ability to activate complement, demonstrating a high affinity for FcγRI, FcγRII, FcγRIIa, and FcγRIII receptors. They also possess the longest and most flexible hinge region among the IgG subclasses.[36–38] Previous studies have reported the role of IgG3 antibodies in the immune-mediated control of certain pathogens, for example, in the induction of long-term protection against malaria[39] and Chikungunya virus.[40] In the setting of HIV, HIV-1-specific IgG3 has been shown to be correlated with a decreased risk of HIV-1 infection in human clinical trials,[41] and IgG3 bnAbs have been found to exhibit improved binding to FcγRIIa, which correlated with enhanced phagocytosis and increased antibody-dependent cellular cytotoxicity.[42]

Our findings using the BG18 BCR knockin adoptive transfer model highlight the potential advantages of using TM immunogens encoded by replicon RNA in the context of germline-targeting strategies for HIV vaccines. We recently reported that mRNA vaccines encoding a TM stabilized Env trimer enabled neutralizing-precursor B cells with a lower affinity to be recruited to GCs, compared to immunization with a soluble trimer protein and adjuvant.[14] In the BCR knockin adoptive transfer model of germline-targeting immunization, the TM trimer format recruited a higher frequency of bnAb precursor B cells in the early stages of the GC response and greatly increased the serum antibody response recognizing the target epitope on Env. The serum response also coincided with generation of higher $CD45.2^+$ BG18 plasma cells in response to TM Env immunogen. We hypothesize that these findings may be a result of the TM form of the antigen lowering competitor B cell responses against irrelevant base epitopes of the trimer and/or greater stability of the TM immunogen leading to more effective presentation of the intact BG18 epitope *in vivo*. Another possible mechanism is that membrane anchoring enhances B cell activation via mechanosensitive calcium channels such as Piezo1, which are engaged during antigen extraction.[43]

Similar patterns of mutations were observed in the HC across immunization groups, however, additional boosting with immunogens bearing an increasing number of native-Env features will be necessary to evaluate whether the TM format better supports the maturation of bnAb precursors toward broad neutralization.[31] Notably, serum antibody responses targeting the BG18 epitope did not differ between the TM and soluble trimer replicons in WT mice, underscoring the critical role of precursor frequency in shaping the early antibody response. Our study builds upon and extends prior work by Aldon et al., who demonstrated that TM replicon immunogens enhanced early IgG responses and IgG2a class switching compared to soluble trimers, but used a non-germline-targeting Env and did not assess precursor B cell recruitment.[15] In contrast, our immunogen was specifically designed for germline targeting, and we directly tracked rare bnAb precursor B cells using the BG18 BCR knockin adoptive transfer model.

In summary, these studies have identified several elements of the immune response that are altered by changing the physical form of an encoded HIV Env immunogen when delivered as a self-amplifying RNA. Notably, antigen-specific GC responses and LN cytokine/CK induction were altered simply by changing the form of the Env immunogen encoded by the replicon. Further optimization of replicon-encoded immunogen design may enable these differences to be exploited to further enhance priming of bnAb precursor B cells for HIV and could be useful for promoting protective immune responses against other viral pathogens.

MATERIALS AND METHODS

Replicon synthesis

Replicon RNA was produced by *in vitro* transcription as described previously with slight modifications.[44] In brief, DNA plasmids containing the replicon, flanked by a T7 promoter on the 5′ end and encoding a poly(A) sequence followed immediately by MluI restriction sites on the 3′ end, was amplified in *Escherichia coli* and purified

using the Plasmid QIAprep spin midi kit (QIAGEN). The amplified DNA was linearized with MluI restriction enzyme (New England Biolabs) and was then transcribed using HiScribe T7 High Yield RNA Synthesis Kit (New England Biolabs) and purified using PureLink RNA Mini columns (Thermo Fisher). The transcript was then post-transcriptionally capped with methylation (ScriptCap Cap 1 Capping System, CellScript). After capping, the replicon RNA was purified a final time using PureLink RNA Mini columns (Thermo Fisher). The quality of the resulting replicons was assessed using ultraviolet-visible light spectrophotometry and gel electrophoresis.

Formulation and characterization of replicon-encapsulating LNPs

To encapsulate replicons in LNPs, lipids including *N*1,*N*3,*N*5-Tris (3-(didodecylamino)propyl)benzene-1,3,5-tricarboxamide (TT3, synthesized as described previously[45]), 1,2-dioleoyl-*sn*-glycero-3-phosphoethanolamine (DOPE, Avanti Polar Lipids), cholesterol (Chol, Avanti Polar Lipids), and 1,2-dimyristoyl-*sn*-glycero-3-phosphoethanolamine-*N*-[methoxy(polyethylene glycol)-2000 (DMPE-PEG, Avanti Polar Lipids) were dissolved in ethanol at a 22:33:44:1 TT3/DOPE/Chol/DMPE-PEG molar ratio and at a total concentration of 5.8 μg/mL. RNA was dissolved in 100 mM citrate buffer, pH 3, at 60 μg/mL, and then combined with the lipid solution at an N:P ratio of 2:1 (N, number of nitrogens on TT3 ionizable lipid; P, number of phosphate groups on RNA) using an Ignite microfluidic mixing system (Precision Nanosystems) at a flow rate of 12 mL/min and an ethanol:aqueous volume ratio of 1:2. The resulting replicon-loaded LNPs were then dialyzed against PBS using a 20K molecular weight cutoff Slide-A-Lyzer mini dialysis device (Thermo Fisher). Particles were characterized for size and zeta potential using Zetasizer Nano ZSP equipment (Malvern) and imaged by cryoelectron microscopy using a JEOL 2100F transmission electron microscope operating at an acceleration voltage of 200 kV. The replicon encapsulation efficiency was quantified using a RiboGreen RNA assay kit (Thermo Fisher) following the manufacturer's instructions. Briefly, replicon-loaded LNPs were resuspended in Tris-EDTA without or with Triton X-100 (0.5% v/v) to disrupt the particles. RNA encapsulation efficiency was defined as the percentage of replicon encapsulated in LNPs relative to total replicon.

Synthesis of N332-GT2 protein immunogen and saponin adjuvant

N332-GT2 trimer protein bearing a C-terminal 6× His-tag was produced in 293F cells using the Expi293 expression system (Thermo Fisher, catalog no. A14524) following the manufacturer's instructions for culturing and transfection. Following expression, the His-tagged protein was purified from the supernatant of 293F cells by Ni-NTA affinity chromatography using a 5-mL HisTrap HP column on an ÄKTA Pure 25-L fast protein liquid chromatography system (Cytiva Life Sciences, catalog no. 29018224). The column was equilibrated in PBS running buffer, and 293F supernatant was applied at 5 mL/min. After washing the column with PBS to remove unbound protein, the His-tagged trimer was eluted with 0.5 M imidazole in PBS buffer (pH 7.4) and exchanged into PBS using Amicon Ultra-4 centrifugal filtration systems (Millipore). SMNP saponin adjuvant was produced as previously described.[46]

Antigenicity characterization of replicon-encoded TM and secreted trimer

Antigenicity profiles of replicon-encoded TM and secreted trimers were evaluated *in vitro* by flow cytometry and ELISA, respectively. The following neutralizing and non-neutralizing human monoclonal antibodies were used: PG9, PGT121, 3074, 35O22, A32, PCT64, PGT145, PGT151, 4025, BG18, and RM19R. The RM19R, PCT64, PGT145, PGT151, 4025, and BG18 antibodies were produced in HEK293F cells (Thermo Fisher) and purified using Protein A affinity chromatography (Cytiva) following established protocols.[47] All other antibodies were sourced from BEI Resources (NIH, National Institute of Allergy and Infectious Diseases).

For TM trimer, C2C12 mouse skeletal muscle cells (American Type Culture Collection) were transfected with replicons by electroporation using a Neon transfection system (1,400 V, 20 ms, 1 pulse) following the manufacturer's instructions. At 24 h post-transfection, cells were harvested by trypsinization, washed by PBS, and incubated with Zombie Aqua fixable viability dye for 15 min at 25°C. Then, they were washed with flow cytometry buffer (2% fetal bovine serum [FBS], 3 mM EDTA in PBS). Cells were then incubated with flow cytometry buffer containing 2 μg/mL of the primary monoclonal antibodies for 20 min at 25°C, washed, and stained with phycoerythrin (PE)-Cy5 conjugated anti-human IgG secondary antibody (BD Biosciences) at a 1:5 dilution in flow cytometry buffer for 20 min at 25°C. Stained cells were analyzed on a Becton Dickinson Symphony flow cytometer, and data were processed using FlowJo version 10 software.

A sandwich ELISA assay was used for antigenicity characterization of the replicon-encoded secreted trimer. To obtain enough protein for the assay, BHK-21 cells were chosen for transfection as they are known to be defective in the IFN pathway, allowing for high protein yield. BHK-21 cells were electroporated with secreted trimer-encoding replicon, and the cell supernatant was collected 24 h later. The concentration of trimer in the supernatants was measured by ELISA using human and mouse VRC01 antibodies as coating and detection antibodies, respectively. High-binding half-area 96-well Costar plates (Corning) were coated with supernatant diluted in PBS to a 2-μg/mL trimer concentration, and then plates were blocked with diluent buffer (2% BSA in PBS). After a wash step, serial dilutions of the indicated monoclonal antibodies were added to the plate and incubated for 2 h at 25°C. Plates were then washed and incubated with goat HRP (horseradish peroxidase)-conjugated anti-human IgG (Jackson ImmunoResearch) in diluent buffer for 1 h at 25°C. After a final wash, plates were developed with 3,3′,5,5′-tetramethylbenzidine (TMB) substrate (1-step Ultra TMB-ELISA Substrate Solution, Thermo Fisher) for 10 min, following which the reaction was stopped with 2 N H_2SO_4, and absorbance

readings at 450 nm and a reference wavelength of 540 nm were read using a Tecan Infinite 200PRO plate reader.

Animals and immunizations

Female BALB/c mice (strain: 000651, 6–8 weeks old) or male (8–10 weeks old) CD45.1$^{+/+}$ mice (B6.SJL-Ptprc^{a} Pepc^{b}/BoyJ) were purchased from The Jackson Laboratory. Mice B cells expressing a humanized BG18 BCR were generated as before.[48] Mice were housed in a 12-h light-dark cycle with *ad libitum* access to water and standard laboratory chow. All animal procedures were conducted in accordance with protocols approved by the Institutional Animal Care and Use Committee and conducted in accordance with the regulations of the Association for Assessment and Accreditation of Laboratory Animal Care International. B cells from donor BG18 IgH$^{+/WT}$ were isolated using a Pan B cell isolation kit II (Miltenyi Biotec) and were adoptively transferred through tail vein injection into acceptor B6.SJL-Ptprc^{a} Pepc^{b}/BoyJ (CD45.1 mice) at the indicated frequencies. Mice were immunized 24 h post-adoptive transfer. Groups of mice were injected bilaterally in the gastrocnemius muscles with LNP-encapsulated replicons at 2 μg total replicon dose (1 μg per injection site) or subcutaneously at the base of the tail with 10 μg protein trimer antigen and 5 μg SMNP total, administered as two separate injections with the dose split equally between the two sites.

ELISA measurement of antibody titers

Anti-trimer serum Ig titers were assessed by ELISA. Half-area high-binding 96-well plates (Corning Life Sciences) were coated overnight at 4°C with 1 μg/mL (50 μL/well) streptavidin in PBS. Coated plates were blocked with 2% BSA in PBS and incubated overnight at 4°C. Plates were then washed and incubated with 1 μg/mL biotinylated trimer in block buffer (2% BSA in PBS) at for 2 h at 25°C, following which serial dilutions of serum samples (1:20–1:160 followed by serial 4-fold dilution) were added to the plates. After 2 h of incubation at 25°C, plates were washed with incubated with HRP-conjugated anti-mouse IgG, IgG1, IgG2a, IgG2b, and IgG3 (Bio-Rad), diluted 1:5,000 in block buffer. The reaction mixture was incubated at 25°C for 30 min. At the end of the incubation period, the plates were washed and treated with 50 μL TMB substrate. The reaction was stopped after 10 min with a 1-M H_2SO_4 solution. The plates were read on a Tecan Infinite 200PRO plate reader at 450 nm with background correction at 540 nm. The serum titer was determined as the logarithm of the reciprocal of the serum dilution that resulted in absorbance readings exceeding 0.1 OD unit above background.

For measuring serum titer in BG18 adoptive transfer experiments, plates were coated overnight at 4°C with 1 μg/mL (50 μL/well) of anti-His antibody in PBS. After overnight incubation, plates were washed and coated overnight at 4°C with 1 μg/mL (50 μL/well) of N332-GT2 or GT2-KO His-tagged protein. After incubation, plates were washed with PBS containing 0.01% Tween 20 (PBST) and blocked for 1 h with blocking buffer PBST containing 3% BSA. Post-wash, plates were incubated with serial dilutions of serum samples (1:100 and followed by serial 3-fold dilutions) overnight at 4°C. After incubation, plates were washed and incubated with alkaline phosphatase conjugated anti-mouse IgG (Jackson Immuno Research) diluted 1:1,000 in PBST containing 0.5% BSA. The reaction mixture was incubated at 25°C for 1 h and washed. Post-wash, the ELISA was developed with p-nitrophenyl phosphate (Sigma) and stopped after 30 min with 3 N NaOH post-color development. Absorbance was measured at 405 nm using a BioTek plate reader (Synergy Neo2).

Construction of antigen tetramers

To construct tetramers of N332-GT2 trimers for identification of antigen-specific B cells, an Avi-tagged version was of the trimer was recombinantly synthesized and purified by His-tag affinity chromatography as above. The Avi-tagged trimer was first biotinylated using a BirA biotinylation kit (Avidity) following the manufacturer's protocol. The biotinylated trimer solution was then centrifuge filtered with a 10-kDa Amicon centrifugal filter unit to remove free biotin and exchange its buffer into PBS. On the day of staining, the biotinylated trimer was crosslinked with APC-, APC-Cy7-, BV421-, and BV605-labeled streptavidin (Thermo Fisher) to form the antigen tetramer by mixing in a 5:1 trimer:streptavidin molar ratio. Tetramers were added to the cells at a concentration of 20 nM in flow cytometry buffer for staining.

Cytokine analysis

Cytokine levels were measured in serum and dLNs at 6, 24, 48, and 96 h post-immunization using a LEGENDplex antivirus response panel (BioLegend, catalog no. 740622) following the manufacturer's protocol. At each time point, blood samples were collected in serum gel tubes with clotting activator and spun down to isolate the serum. Following blood collection, mice were sacrificed, and dLNs (popliteal and iliac LNs for replicon immunizations and inguinal LNs for protein immunizations) were removed. The soluble content of LNs was extracted by smashing LNs in PBS containing 2% BSA and protease inhibitors (Thermo Fisher), followed by centrifugation to remove cells and debris. Sera and LN extracts were then flash-frozen and stored at −80 until analysis.

T cell ELISpot

IFN-γ ELISpot assay was used to quantify antigen-specific effector T cells. Spleens were excised and smashed through a 70-μm cell strainer into single-cell suspensions. Following red blood cell lysis with ACK buffer, splenocytes were resuspended in ELISpot complete media (RPMI 1640 containing 10% FBS, penicillin-streptomycin [100 U/mL], and 1 mM sodium pyruvate) and seeded at 10^6 cells/well in mouse IFN-γ ELISpot plates (BD). Next, splenocytes were stimulated for a period of 20 h in a 37°C/5% CO_2 incubator with a pool of overlapping peptides spanning the entire trimer protein sequence (15-mer peptides overlapping by 11 residues). Plates were then washed and incubated with biotinylated anti-IFN-γ detection antibody followed by streptavidin-HRP for 2 h and 1 h, respectively, with wash steps after each incubation period. Plates were developed with substrate BCIP/NBT chromogen (MABTECH) for 30 min following the manufacturer's instructions, quenched with

water, dried overnight, and scanned using a CTL-ImmunoSpot Plate Reader. Spot counting was performed by a blinded investigator using CTL-ImmunoSpot software.

Memory B cell analysis

Splenocytes were harvested and processed as described above. Splenocytes were resuspended in PBS, plated in a 96-well plate, and stained with Zombie Aqua Fixable Viability dye (BioLegend) for exclusion of dead cells. After washing and resuspension in flow cytometry buffer, cells were incubated with APC/Cy7- and BV421-labeled antigen tetramer followed by the following antibodies: B220-PE-Cy7 (BioLegend, clone RA3-6B2), CD3-PerCP-Cy5 (BioLegend, clone 145-2C11), GL7-FITC ([fluorescein isothiocyanate] BioLegend, clone GL7), CD38-BV711 (BD Biosciences, clone 90), IgD-APC (Invitrogen, clone 11-26c), CD138-PE (BioLegend, clone 281-2), CD73-BV605 (BioLegend, clone TY/11.8), and PDL2-BUV395 (BD Biosciences, clone TY25). Next, cells were washed and fixed in 4% (v/v) paraformaldehyde for 15 min at 25°C. Stained samples were analyzed on a Becton Dickinson symphony flow cytometer (BD Biosciences) and data processed using FlowJo software. After exclusion of doublets and dead cells, antigen-specific memory B cells were defined as $CD3^{-}$ $BB220^{+}$ $GL7^{-}$ $CD38^{+}$ IgD^{-} $CD138^{-}$ $PDL2^{+}$ $CD73^{+}$ cells that were dually stained with BV421- and APC/Cy7-conjugated trimer.

GC analysis

The inguinal, iliac, and popliteal LNs were harvested from mice at weeks 2, 4, 6, or 8 post-i.m. immunizations. To obtain single-cell suspensions, the LNs were filtered through a 70-μm cell strainer. The resulting cells were then subjected to staining for viability (Zombie Aqua Fixable Viability Kit, BioLegend) and against CD4 (BV711, BioLegend, clone RM4-5), B220 (PE-Cy7, BioLegend, clone RA3-6B2), CD38 (FITC, BioLegend, clone 90), CXCR5 (PE, BioLegend, clone L138D7), PD-1 (BV421, BioLegend, clone 29F.1A12), and GL7 (PerCP-Cy5.5, BioLegend, clone GL7). Antigen-specific staining was done using biotinylated trimer conjugated to either streptavidin-BV605 (BioLegend) or streptavidin-APC (BioLegend). Flow cytometry analysis was performed using a Becton Dickinson Symphony instrument, and the resulting data were processed using FlowJo software.

Bone marrow ELISpot

ELISpot assays were used to enumerate ASCs and were run according to the manufacturer's protocol (Mabtech). Bone marrow cells were harvested from the femur and tibia of mice by clipping the ends and flushing the bone cavity with harvest buffer. Bone marrow cells were then treated with ACK buffer to lyse red blood cells and passed through a 70-μm strainer to obtain single-cell suspensions. Following counting, cells were resuspended at different dilutions in ELISpot complete media. Cell dilutions were then added to wells of 96-well polyvinylidene difluoride ELISpot plates (MilliporeSigma, catalog no. MSIPS4510) that had been preactivated with 35% ethanol, coated with anti-mouse IgG at 15 μg/mL in sterile PBS overnight at 4°C, and blocked with complete media for at least 30 min. Following 20 h of incubation at 37°C/5% CO_2, plates were washed and then incubated with biotinylated antigen (1 μg/mL) and biotinylated anti-mouse IgG antibody (1 μg/mL) in PBS with 0.5% BSA for total IgG and antigen-specific IgG responses, respectively, for 1 h at 25°C. Plates were washed again and incubated with 1:1,000 streptavidin-alkaline phosphatase in PBS with 0.5% BSA for 1 h at 25°C. After a final wash step, bromochloroindolyl phosphate–nitro blue tetrazolium substrate (Mabtech, catalog no. 3650-10) was added to plates and developed for 30 min, quenched with water, air-dried, and quantified on a CTL-ImmunoSpot analyzer.

Glycopeptide analysis by MS

To investigate the glycan composition of N332-GT2 trimers expressed from replicons, we electroporated replicon encoding *env* trimer into HEK293F cells. The transfected cells were harvested after 3 days and were pelleted down, and the desired protein was purified from the supernatant using *Galanthus nivalis* lectin beads. The purified protein was subsequently reduced and alkylated for glycan analysis using LC-MS. For the determination of glycan analysis of recombinant protein, we transiently transfected plasmid DNA into mammalian cells using polyethyleneimine. After 7 days, the transfected cells were harvested, and the supernatant was purified in the same way as replicon expressed protein. To compare the glycan analysis of recombinant protein and protein expressed via replicon expression, we have used the same conditions and parameters for glycan analysis using LC-MS.

Env proteins were denatured for 1 h in 50 mM Tris/HCl, pH 8.0, containing 6 M urea. Next, the sample was reduced and alkylated by adding 5 mM dithiothreitol (DTT) and 20 mM iodoacetamide (IAA) and incubated for 1 h in the dark, followed by a 1-h incubation with 20 mM DTT to eliminate residual IAA. The alkylated Env glycoproteins were buffer exchanged into 50 mM Tris/HCl, pH 8.0, using Vivaspin columns (3 kDa), and three aliquots were digested separately overnight using trypsin (MS grade, Promega), chymotrypsin (MS grade, Promega), or alpha lytic protease (Sigma-Aldrich) at a ratio of 1:30 (w/w) at 37°C. The next day, the peptides were dried and extracted using C18 Zip-tip (MerckMilipore). Following the extraction, the peptides were dried again, re-suspended in 0.1% formic acid, and analyzed by nano-LC-electrospray ionization-MS with an Ultimate 3000 high-performance LC (Thermo Fisher) system coupled to an Orbitrap Eclipse mass spectrometer (Thermo Fisher) using stepped higher energy collision-induced dissociation (HCD) fragmentation. Peptides were separated using an EasySpray PepMap rapid separation LC C18 column (75 μm × 75 cm). A trapping column (PepMap 100 C18, 3 μm particle size, 75 μm × 2 cm) was used in line with the LC prior to separation with the analytical column. The LC-MS gradient lasted for 280 min. For the first 260 min, a linear gradient ranging from 4% to 32% acetonitrile (ACN) in 0.1% formic acid was applied. This was followed by a 20-min alternating gradient between 76% ACN in 0.1% formic acid and 4% ACN in 0.1% formic acid, used to ensure all of the sample had eluted from the column. The flow rate was set to 300 nL/min. The spray voltage was set to

2.5 kV, and the temperature of the heated capillary was set to 40°C. The ion transfer tube temperature was set to 275°C. The scan range was 375–1,500 *m/z*. Stepped HCD collision energy was set to 15%, 25%, and 45% and the MS2 for each energy was combined. Precursor and fragment detection were performed using an Orbitrap at a resolution MS1 = 120,000 and MS2 = 30,000. The AGC target for MS1 was set to standard and injection time was set to auto, which involves the system setting the two parameters to maximize sensitivity while maintaining cycle time. Full LC and MS methodology can be extracted from the appropriate Raw file using XCalibur FreeStyle software or upon request.

Glycopeptide fragmentation data were extracted from the raw file using Byos (version 4.0; Protein Metrics). The glycopeptide fragmentation data were evaluated manually for each glycopeptide; the peptide was scored as true positive when the correct b and y fragment ions were observed along with oxonium ions corresponding to the glycan identified. The MS data were searched using the Protein Metrics 305 N-glycan library, with sulfated glycans added manually. The relative amounts of each glycan at each site as well as the unoccupied proportion were determined by comparing the extracted chromatographic areas for different glycotypes with an identical peptide sequence. All charge states for a single glycopeptide were summed. The precursor mass tolerance was set at 4 and 10 ppm for fragments. A 1% false discovery rate was applied. The relative amounts of each glycan at each site as well as the unoccupied proportion were determined by comparing the extracted ion chromatographic areas for different glycopeptides with an identical peptide sequence. Glycans were categorized according to the composition detected. HexNAc(2)Hex(9–3) was classified as M9–M3. Any of these compositions that were detected with a fucose are classified as fucose-modified. HexNAc(3)Hex(5–6)Neu5Ac)(0–4) was classified as Hybrid with HexNAc(3)Hex(5–6)Fuc(1)NeuAc(0–1) classified as Fhybrid. Complex-type glycans were classified according to the number of processed antenna and fucosylation. Complex glycans are categorized as HexNAc(3)(X), HexNAc(3)(F)(X), HexNAc(4)(X), HexNAc(4)(F)(X), HexNAc(5)(X), HexNAc(5)(F)(X), HexNAc(6+)(X), and HexNAc(6+)(F)(X). Core glycans are any glycan smaller than HexNAc(2)Hex(3).

Post-immunization analysis of BG18 B cell adoptively transferred mice

The inguinal, iliac, and popliteal LNs were harvested from mice at 2, 4, or 6 weeks post-immunization via i.m. and subcutaneous routes for RNA and protein vaccines, respectively. Cells were harvested by gently crushing them and passing them through a 70-μM strainer. The cells were then resuspended in PBS containing 500-fold diluted Live/Dead Blue (Thermo Scientific) and 200-fold diluted FcR Blocking reagent (purified rat anti-mouse CD16/CD32, BD Biosciences) for 20 min at 4°C. After washing, antigen staining was done using biotinylated trimer conjugated to either streptavidin-Alexa 488 (BioLegend) or streptavidin-Alexa 647 (BioLegend) for 30 min at 4°C. After washing off excess antigen, cells were incubated with an antibody cocktail containing CD4, CD8, F4/80, GR-1 (APC-eFluor 780, eBioscience, clone RM4-5, 53-6.7, BM8, RB6-8C5, respectively), B220 (BUV395, BD Biosciences, clone RA3-6B2), CD38 (BUV563, BD Biosciences, clone 90), CD95 (PE-Cy7, BioLegend, clone L138D7), CD45.1 (Alexa 700, BioLegend, clone A20), CD45.2 (Brilliant Blue 700, BD Biosciences, clone 104), CD138 (BV650, BD Biosciences, clone 281-2), IgD (BV605, BD Biosciences, 11-26c.2a clone), and GL7 (PE, BioLegend, GL7 clone). For sorting, Live/Dead stain was replaced with DAPI (1,000-fold dilution). The antibodies used for sorting were CD4, CD8, F4/80, GR-1 (APC-eFluor 780, eBioscience, clone RM4-5, 53–6.7, BM8, RB6-8C5, respectively), B220 (BV510, BioLegend, clone RA3-6B2), CD95 (PE-Cy7, BioLegend, clone L138D7), CD45.1 (Alexa 700, BioLegend, clone A20), CD45.2 (Brilliant Blue 700, BD Biosciences, clone 104), and IgD (BV650, BioLegend, clone 11-26c.2a). Cells were washed three times to remove any excess antibodies.

Cell sorting and sequencing

For sorting, cells from each individual mouse were barcoded with TotalSeq-C anti-mouse hashtag antibodies. A total of nine hashtags were used. Samples were sorted onto PCR tubes containing flow cytometry buffer. Sorted cells were then encapsulated and next-generation sequencing libraries were prepared following the 10x Genomics Chromium Next GEM Single Cell 5′ Reagent Kits version 2 protocol. TapeStation Systems D5000 high-sensitivity ScreenTape assay (Agilent) was used to measure library size. After quantifying the libraries through Qubit dsDNA High Sensitivity (Invitrogen), they were pooled and were run on the NextSeq 550 System (Illumina). Analysis was performed using the Cell Ranger version 6 software pipeline (10x Genomics) with a customized reference database. For sorting on week 6, single cells were sorted on 96-well plates as described previously.[18] BCR sequencing on single-sorted 96-well plates were done as before.[18,31] Following the reverse transcriptase step and BCR amplification (PCR-1 and PCR-2 nested PCR reaction), Sanger sequencing was done on individual samples (GENEWIZ).[31] Sequencing data were analyzed using Geneious Biologics software and IMGT/V-Quest.

DATA AVAILABILITY

All data are available upon request from the authors.

ACKNOWLEDGMENTS

We thank the Koch Institute Swanson Biotechnology Center's Flow Cytometry core facilities for their technical support. This work was supported in part by the Koch Institute Support (core) Grant (5P30-CA014051) from the National Cancer Institute, the NIH (award no. AI164829 to P.Y., award no. AI176533 to D.J.I., and award no. UM1AI144462 to F.D.B., M.C., W.R.S., and D.J.I.), and the Ragon Institute of Massachusetts General Hospital, Massachusetts Institute of Technology, and Harvard University. D.J.I. is an investigator of the Howard Hughes Medical Institute. This work was also supported by the International AIDS Vaccine Initiative Neutralizing Antibody Center through the Collaboration for AIDS Vaccine Discovery grant no. OPP1196345/INV-008813 funded by the Gates Foundation (to M.C.).

AUTHOR CONTRIBUTIONS

P.Y. and D.J.I. conceptualized the study, interpreted the data, and wrote the manuscript. P.Y. completed the *in vitro* antigen characterization and *in vivo* mouse studies. T.K.R. carried out the *in vitro* transcription for replicon RNA synthesis. A.R.G. carried out the BCR knockin adoptive transfer studies. H.C. completed the site-specific glycan analysis. R.Y., J.G., K.S., K.A.R., and M.B.M. assisted with executing the experiments. J.M.S.

designed the N332-GT2 immunogens. Y.Z. synthesized the TT3 ionizable lipid. All authors reviewed and edited the manuscript. Y.D., M.C., W.R.S., F.D.B., and D.J.I. supervised the study.

DECLARATION OF INTERESTS

Y.D. is an inventor on a patent filed by The Ohio State University related to the TT3 lipid used in this study. J.M.S. and W.R.S. are inventors on patent applications concerning N332-GT2 immunogens. W.R.S. is an employee of Moderna; however, his contributions to this study were made before his employment at Moderna. D.J.I. is a co-founder and consultant for Strand Therapeutics. F.D.B. has consultancy relationships with Adimab, Third Rock Ventures, and the EMBO Journal, and founded BliNK Therapeutics.

SUPPLEMENTAL INFORMATION

Supplemental information can be found online at https://doi.org/10.1016/j.ymthe.2025.07.036.

REFERENCES

1. Chaudhary, N., Weissman, D., and Whitehead, K.A. (2021). mRNA vaccines for infectious diseases: principles, delivery and clinical translation. Nat. Rev. Drug Discov. *20*, 817–838. https://doi.org/10.1038/s41573-021-00283-5.

2. Barbier, A.J., Jiang, A.Y., Zhang, P., Wooster, R., and Anderson, D.G. (2022). The clinical progress of mRNA vaccines and immunotherapies. Nat. Biotechnol. *40*, 840–854. https://doi.org/10.1038/s41587-022-01294-2.

3. Tong, J., Zhang, W., Chen, Y., Yuan, Q., Qin, N.-N., and Qu, G. (2022). The emerging role of RNA modifications in the regulation of antiviral innate immunity. Front. Microbiol. *13*, 845625. https://doi.org/10.3389/fmicb.2022.845625.

4. Boo, S.H., and Kim, Y.K. (2020). The emerging role of RNA modifications in the regulation of mRNA stability. Exp. Mol. Med. *52*, 400–408. https://doi.org/10.1038/s12276-020-0407-z.

5. Comes, J.D.G., Pijlman, G.P., and Hick, T.A.H. (2023). Rise of the RNA machines – self-amplification in mRNA vaccine design. Trends Biotechnol. *41*, 1417–1429. https://doi.org/10.1016/j.tibtech.2023.05.007.

6. Bloom, K., van den Berg, F., and Arbuthnot, P. (2021). Self-amplifying RNA vaccines for infectious diseases. Gene Ther. *28*, 117–129. https://doi.org/10.1038/s41434-020-00204-y.

7. Blakney, A.K., Ip, S., and Geall, A.J. (2021). An update on self-amplifying mRNA vaccine development. Vaccines *9*, 97. https://doi.org/10.3390/vaccines9020097.

8. Saraf, A., Gurjar, R., Kaviraj, S., Kulkarni, A., Kumar, D., Kulkarni, R., Virkar, R., Krishnan, J., Yadav, A., Baranwal, E., et al. (2024). An Omicron-specific, self-amplifying mRNA booster vaccine for COVID-19: a phase 2/3 randomized trial. Nat. Med. *30*, 1363–1372. https://doi.org/10.1038/s41591-024-02955-2.

9. Geall, A.J., Verma, A., Otten, G.R., Shaw, C.A., Hekele, A., Banerjee, K., Cu, Y., Beard, C.W., Brito, L.A., Krucker, T., et al. (2012). Nonviral delivery of self-amplifying RNA vaccines. Proc. Natl. Acad. Sci. *109*, 14604–14609. https://doi.org/10.1073/pnas.1209367109.

10. Vogel, A.B., Lambert, L., Kinnear, E., Busse, D., Erbar, S., Reuter, K.C., Wicke, L., Perkovic, M., Beissert, T., Haas, H., et al. (2018). Self-amplifying RNA vaccines give equivalent protection against influenza to mRNA vaccines but at much lower doses. Mol. Ther. *26*, 446–455. https://doi.org/10.1016/j.ymthe.2017.11.017.

11. Maine, C.J., Miyake-Stoner, S.J., Spasova, D.S., Picarda, G., Chou, A.C., Brand, E.D., Olesiuk, M.D., Domingo, C.C., Little, H.J., Goodman, T.T., et al. (2025). Safety and immunogenicity of an optimized self-replicating RNA platform for low dose or single dose vaccine applications: a randomized, open label Phase I study in healthy volunteers. Nat. Commun. *16*, 456. https://doi.org/10.1038/s41467-025-55843-9.

12. Oda, Y., Kumagai, Y., Kanai, M., Iwama, Y., Okura, I., Minamida, T., Yagi, Y., Kurosawa, T., Chivukula, P., Zhang, Y., and Walson, J.L. (2024). Persistence of immune responses of a self-amplifying RNA COVID-19 vaccine (ARCT-154) versus BNT162b2. Lancet Infect. Dis. *24*, 341–343. https://doi.org/10.1016/s1473-3099(24)00060-4.

13. Elliott, T., Cheeseman, H.M., Evans, A.B., Day, S., McFarlane, L.R., O'Hara, J., Kalyan, M., Amini, F., Cole, T., Winston, A., et al. (2022). Enhanced immune responses following heterologous vaccination with self-amplifying RNA and mRNA COVID-19 vaccines. Plos Pathog. *18*, e1010885. https://doi.org/10.1371/journal.ppat.1010885.

14. Melzi, E., Willis, J.R., Ma, K.M., Lin, Y.-C., Kratochvil, S., Berndsen, Z.T., Landais, E. A., Kalyuzhniy, O., Nair, U., Warner, J., et al. (2022). Membrane-bound mRNA immunogens lower the threshold to activate HIV Env V2 apex-directed broadly neutralizing B cell precursors in humanized mice. Immunity *55*, 2168–2186.e6. https://doi.org/10.1016/j.immuni.2022.09.003.

15. Aldon, Y., McKay, P.F., Moreno Herrero, J., Vogel, A.B., Lévai, R., Maisonnasse, P., Dereuddre-Bosquet, N., Haas, H., Fábián, K., Le Grand, R., et al. (2021). Immunogenicity of stabilized HIV-1 Env trimers delivered by self-amplifying mRNA. Mol. Ther. Nucleic Acids *25*, 483–493. https://doi.org/10.1016/j.omtn.2021.06.008.

16. Li, Y., Su, Z., Zhao, W., Zhang, X., Momin, N., Zhang, C., Wittrup, K.D., Dong, Y., Irvine, D.J., and Weiss, R. (2020). Multifunctional oncolytic nanoparticles deliver self-replicating IL-12 RNA to eliminate established tumors and prime systemic immunity. Nat. Cancer *1*, 882–893. https://doi.org/10.1038/s43018-020-0095-6.

17. Melo, M., Porter, E., Zhang, Y., Silva, M., Li, N., Dobosh, B., Liguori, A., Skog, P., Landais, E., Menis, S., et al. (2019). Immunogenicity of RNA replicons encoding HIV Env immunogens designed for self-assembly into nanoparticles. Mol. Ther. *27*, 2080–2090. https://doi.org/10.1016/j.ymthe.2019.08.007.

18. Steichen, J.M., Lin, Y.-C., Havenar-Daughton, C., Pecetta, S., Ozorowski, G., Willis, J.R., Toy, L., Sok, D., Liguori, A., Kratochvil, S., et al. (2019). A generalized HIV vaccine design strategy for priming of broadly neutralizing antibody responses. Science *366*, eaax4380. https://doi.org/10.1126/science.aax4380.

19. Sok, D., Doores, K.J., Briney, B., Le, K.M., Saye-Francisco, K.L., Ramos, A., Kulp, D. W., Julien, J.-P., Menis, S., Wickramasinghe, L., et al. (2014). Promiscuous glycan site recognition by antibodies to the high-mannose patch of gp120 broadens neutralization of HIV. Sci. Transl. Med. *6*, 236ra63. https://doi.org/10.1126/scitranslmed.3008104.

20. Willis, J.R., Berndsen, Z.T., Ma, K.M., Steichen, J.M., Schiffner, T., Landais, E., Liguori, A., Kalyuzhniy, O., Allen, J.D., Baboo, S., et al. (2022). Human immunoglobulin repertoire analysis guides design of vaccine priming immunogens targeting HIV V2-apex broadly neutralizing antibody precursors. Immunity *55*, 2149–2167.e9. https://doi.org/10.1016/j.immuni.2022.09.001.

21. Behrens, A.-J., Vasiljevic, S., Pritchard, L.K., Harvey, D.J., Andev, R.S., Krumm, S.A., Struwe, W.B., Cupo, A., Kumar, A., Zitzmann, N., et al. (2016). Composition and antigenic effects of individual glycan sites of a trimeric HIV-1 envelope glycoprotein. Cell Rep. *14*, 2695–2706. https://doi.org/10.1016/j.celrep.2016.02.058.

22. Dey, A.K., Cupo, A., Ozorowski, G., Sharma, V.K., Behrens, A.J., Go, E.P., Ketas, T.J., Yasmeen, A., Klasse, P.J., Sayeed, E., et al. (2018). cGMP production and analysis of BG505 SOSIP.664, an extensively glycosylated, trimeric HIV-1 envelope glycoprotein vaccine candidate. Biotechnol. Bioeng. *115*, 885–899. https://doi.org/10.1002/bit.26498.

23. Struwe, W.B., Chertova, E., Allen, J.D., Seabright, G.E., Watanabe, Y., Harvey, D.J., Medina-Ramirez, M., Roser, J.D., Smith, R., Westcott, D., et al. (2018). Site-specific glycosylation of virion-derived HIV-1 Env is mimicked by a soluble trimeric immunogen. Cell Rep. *24*, 1958–1966.e5. https://doi.org/10.1016/j.celrep.2018.07.080.

24. Behrens, A.-J., Harvey, D.J., Milne, E., Cupo, A., Kumar, A., Zitzmann, N., Struwe, W.B., Moore, J.P., and Crispin, M. (2017). Molecular architecture of the cleavage-dependent mannose patch on a soluble HIV-1 envelope glycoprotein trimer. J. Virol. *91*, e01894-16. https://doi.org/10.1128/jvi.01894-16.

25. Walker, L.M., Huber, M., Doores, K.J., Falkowska, E., Pejchal, R., Julien, J.-P., Wang, S.-K., Ramos, A., Chan-Hui, P.-Y., Moyle, M., et al. (2011). Broad neutralization coverage of HIV by multiple highly potent antibodies. Nature *477*, 466–470. https://doi.org/10.1038/nature10373.

26. Behrens, A.-J., and Crispin, M. (2017). Structural principles controlling HIV envelope glycosylation. Curr. Opin. Struct. Biol. *44*, 125–133. https://doi.org/10.1016/j.sbi.2017.03.008.

27. McLellan, J.S., Pancera, M., Carrico, C., Gorman, J., Julien, J.-P., Khayat, R., Louder, R., Pejchal, R., Sastry, M., Dai, K., et al. (2011). Structure of HIV-1 gp120 V1/V2 domain with broadly neutralizing antibody PG9. Nature *480*, 336–343. https://doi.org/10.1038/nature10696.

28. Pardi, N., Hogan, M.J., Porter, F.W., and Weissman, D. (2018). mRNA vaccines — a new era in vaccinology. Nat. Rev. Drug Discov. *17*, 261–279. https://doi.org/10.1038/nrd.2017.243.
29. Burton, D.R., and Hangartner, L. (2016). Broadly neutralizing antibodies to HIV and their role in vaccine design. Annu. Rev. Immunol. *34*, 635–659. https://doi.org/10.1146/annurev-immunol-041015-055515.
30. Galkin, A., Chen, Y., Guenaga, J., O'Dell, S., Acevedo, R., Steinhardt, J.J., Wang, Y., Wilson, R., Chiang, C.-I., Doria-Rose, N., et al. (2020). HIV-1 gp120–CD4-induced antibody complex elicits CD4 binding site–specific antibody response in mice. J. Immunol. *204*, 1543–1561. https://doi.org/10.4049/jimmunol.1901051.
31. Xie, Z., Lin, Y.-C., Steichen, J.M., Ozorowski, G., Kratochvil, S., Ray, R., Torres, J.L., Liguori, A., Kalyuzhniy, O., Wang, X., et al. (2024). mRNA-LNP HIV-1 trimer boosters elicit precursors to broad neutralizing antibodies. Science *384*, eadk0582. https://doi.org/10.1126/science.adk0582.
32. Saunders, K.O., Pardi, N., Parks, R., Santra, S., Mu, Z., Sutherland, L., Scearce, R., Barr, M., Eaton, A., Hernandez, G., et al. (2021). Lipid nanoparticle encapsulated nucleoside-modified mRNA vaccines elicit polyfunctional HIV-1 antibodies comparable to proteins in nonhuman primates. npj Vaccin. *6*, 50. https://doi.org/10.1038/s41541-021-00307-6.
33. Mu, Z., Wiehe, K., Saunders, K.O., Henderson, R., Cain, D.W., Parks, R., Martik, D., Mansouri, K., Edwards, R.J., Newman, A., et al. (2022). mRNA-encoded HIV-1 Env trimer ferritin nanoparticles induce monoclonal antibodies that neutralize heterologous HIV-1 isolates in mice. Cell Rep. *38*, 110514. https://doi.org/10.1016/j.celrep.2022.110514.
34. Komori, M., Nogimori, T., Morey, A.L., Sekida, T., Ishimoto, K., Hassett, M.R., Masuta, Y., Ode, H., Tamura, T., Suzuki, R., et al. (2023). saRNA vaccine expressing membrane-anchored RBD elicits broad and durable immunity against SARS-CoV-2 variants of concern. Nat. Commun. *14*, 2810. https://doi.org/10.1038/s41467-023-38457-x.
35. Sun, S., Cai, Y., Song, T.-Z., Pu, Y., Cheng, L., Xu, H., Sun, J., Meng, C., Lin, Y., Huang, H., et al. (2021). Interferon-armed RBD dimer enhances the immunogenicity of RBD for sterilizing immunity against SARS-CoV-2. Cell Res. *31*, 1011–1023. https://doi.org/10.1038/s41422-021-00531-8.
36. Dekkers, G., Bentlage, A.E.H., Stegmann, T.C., Howie, H.L., Lissenberg-Thunnissen, S., Zimring, J., Rispens, T., and Vidarsson, G. (2017). Affinity of human IgG subclasses to mouse Fc gamma receptors. mAbs *9*, 767–773. https://doi.org/10.1080/19420862.2017.1323159.
37. Chu, T.H., Patz, E.F., and Ackerman, M.E. (2021). Coming together at the hinges: Therapeutic prospects of IgG3. mAbs *13*, 1882028. https://doi.org/10.1080/19420862.2021.1882028.
38. Roux, K.H., Strelets, L., and Michaelsen, T.E. (1997). Flexibility of human IgG subclasses. J. Immunol. *159*, 3372–3382.
39. Roussilhon, C., Oeuvray, C., Müller-Graf, C., Tall, A., Rogier, C., Trape, J.-F., Theisen, M., Balde, A., Pérignon, J.-L., and Druilhe, P. (2007). Long-term clinical protection from Falciparum Malaria is strongly associated with IgG3 antibodies to Merozoite surface protein 3. Plos Med. *4*, e320. https://doi.org/10.1371/journal.pmed.0040320.
40. Kam, Y.-W., Simarmata, D., Chow, A., Her, Z., Teng, T.-S., Ong, E.K.S., Rénia, L., Leo, Y.-S., and Ng, L.F.P. (2012). Early appearance of neutralizing immunoglobulin G3 antibodies is associated with Chikungunya virus clearance and long-term clinical protection. J. Infect. Dis. *205*, 1147–1154. https://doi.org/10.1093/infdis/jis033.
41. Yates, N.L., Liao, H.-X., Fong, Y., deCamp, A., Vandergrift, N.A., Williams, W.T., Alam, S.M., Ferrari, G., Yang, Z.y., Seaton, K.E., et al. (2014). Vaccine-induced Env V1-V2 IgG3 correlates with lower HIV-1 infection risk and declines soon after vaccination. Sci. Transl. Med. *6*, 228ra39. https://doi.org/10.1126/scitranslmed.3007730.
42. Richardson, S.I., Ayres, F., Manamela, N.P., Oosthuysen, B., Makhado, Z., Lambson, B.E., Morris, L., and Moore, P.L. (2021). HIV broadly neutralizing antibodies expressed as IgG3 preserve neutralization potency and show improved Fc effector function. Front. Immunol. *12*, 733958. https://doi.org/10.3389/fimmu.2021.733958.
43. Kwak, K., Sohn, H., George, R., Torgbor, C., Manzella-Lapeira, J., Brzostowski, J., and Pierce, S.K. (2023). B cell responses to membrane-presented antigens require the function of the mechanosensitive cation channel Piezo1. Sci. Signal. *16*, eabq5096. https://doi.org/10.1126/scisignal.abq5096.
44. Wroblewska, L., Kitada, T., Endo, K., Siciliano, V., Stillo, B., Saito, H., and Weiss, R. (2015). Mammalian synthetic circuits with RNA binding proteins for RNA-only delivery. Nat. Biotechnol. *33*, 839–841. https://doi.org/10.1038/nbt.3301.
45. Li, B., Luo, X., Deng, B., Wang, J., McComb, D.W., Shi, Y., Gaensler, K.M.L., Tan, X., Dunn, A.L., Kerlin, B.A., and Dong, Y. (2015). An orthogonal array optimization of lipid-like nanoparticles for mRNA delivery in vivo. Nano Lett. *15*, 8099–8107. https://doi.org/10.1021/acs.nanolett.5b03528.
46. Silva, M., Kato, Y., Melo, M.B., Phung, I., Freeman, B.L., Li, Z., Roh, K., Van Wijnbergen, J.W., Watkins, H., Enemuo, C.A., et al. (2021). A particulate saponin/TLR agonist vaccine adjuvant alters lymph flow and modulates adaptive immunity. Sci. Immunol. *6*, eabf1152. https://doi.org/10.1126/sciimmunol.abf1152.
47. Wu, X., Yang, Z.-Y., Li, Y., Hogerkorp, C.-M., Schief, W.R., Seaman, M.S., Zhou, T., Schmidt, S.D., Wu, L., Xu, L., et al. (2010). Rational design of envelope identifies broadly neutralizing human monoclonal antibodies to HIV-1. Science *329*, 856–861. https://doi.org/10.1126/science.1187659.
48. Lin, Y.C., Pecetta, S., Steichen, J.M., Kratochvil, S., Melzi, E., Arnold, J., Dougan, S. K., Wu, L., Kirsch, K.H., Nair, U., et al. (2018). One-step CRISPR/Cas9 method for the rapid generation of human antibody heavy chain knock-in mice. EMBO J. *37*, e99243. https://doi.org/10.15252/embj.201899243.

YMTHE, Volume *33*

Supplemental Information

Augmented humoral responses to HIV Env trimers delivered as transmembrane immunogens by self-replicating RNA

Parisa Yousefpour, Amrit Raj Ghosh, Himanshi Chawla, Rachel Yeung, Justin Gregory, Kristen Si, Tanaka K. Remba, Kristen A. Rodrigues, Mariane B. Melo, Jonathan Dye, Jon M. Steichen, Yuebao Zhang, Yizhou Dong, Max Crispin, William R. Schief, Facundo D. Batista, and Darrell J. Irvine

SUPPLEMENTAL INFORMATION

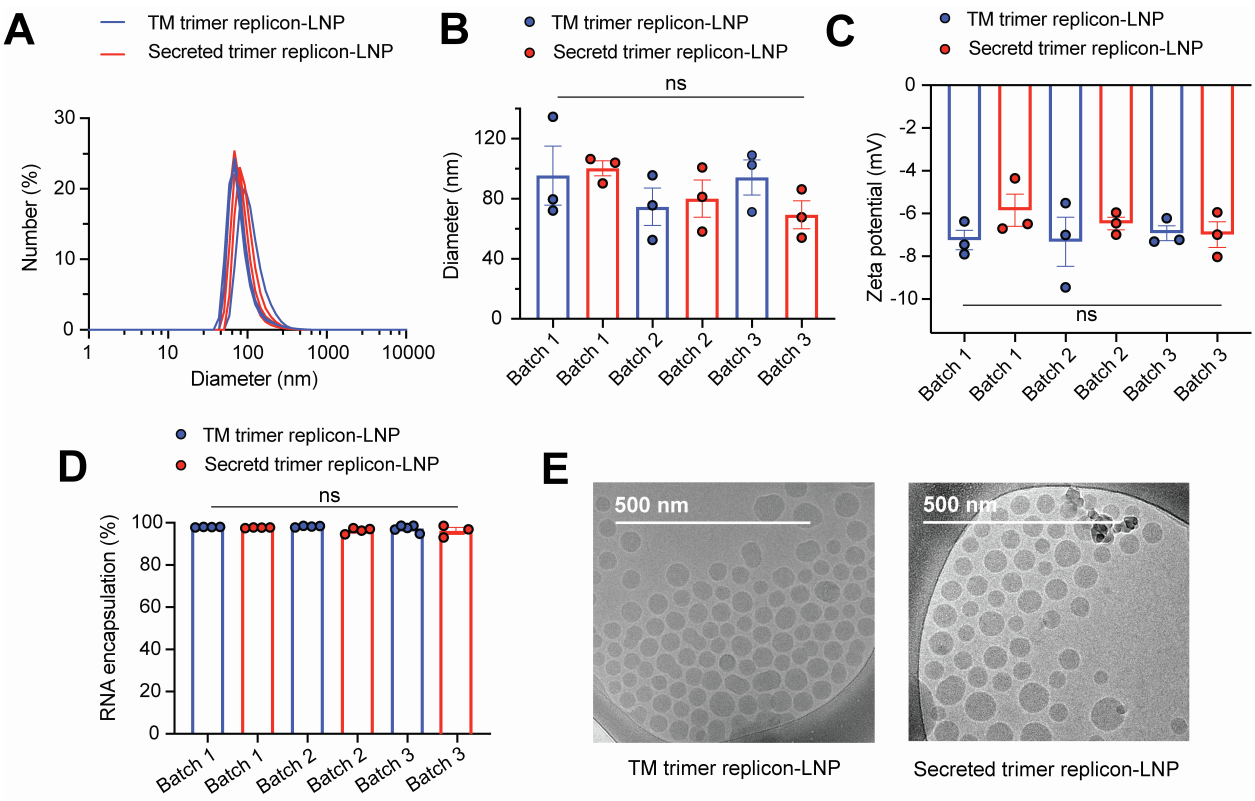

Figure S1. Characterization of LNPs encapsulating replicons encoding TM or secreted HIV Env trimers. (A) Representative dynamic light scattering (DLS) histogram showing number-based particle size distribution from three independently prepared batches. (B–D) Quantification of LNP size, reported as median diameter from the number distribution shown in S1A. (B), zeta potential (C), and RNA encapsulation efficiency (D) across three independent batches for each formulation. (E) Cryo-transmission electron microscopy (cryo-TEM) images of LNPs encapsulating TM or secreted trimer replicons. Statistical comparisons were performed by two-way analysis of variance (ANOVA) followed by Tukey's post hoc test. Data are shown as mean ± SEM.

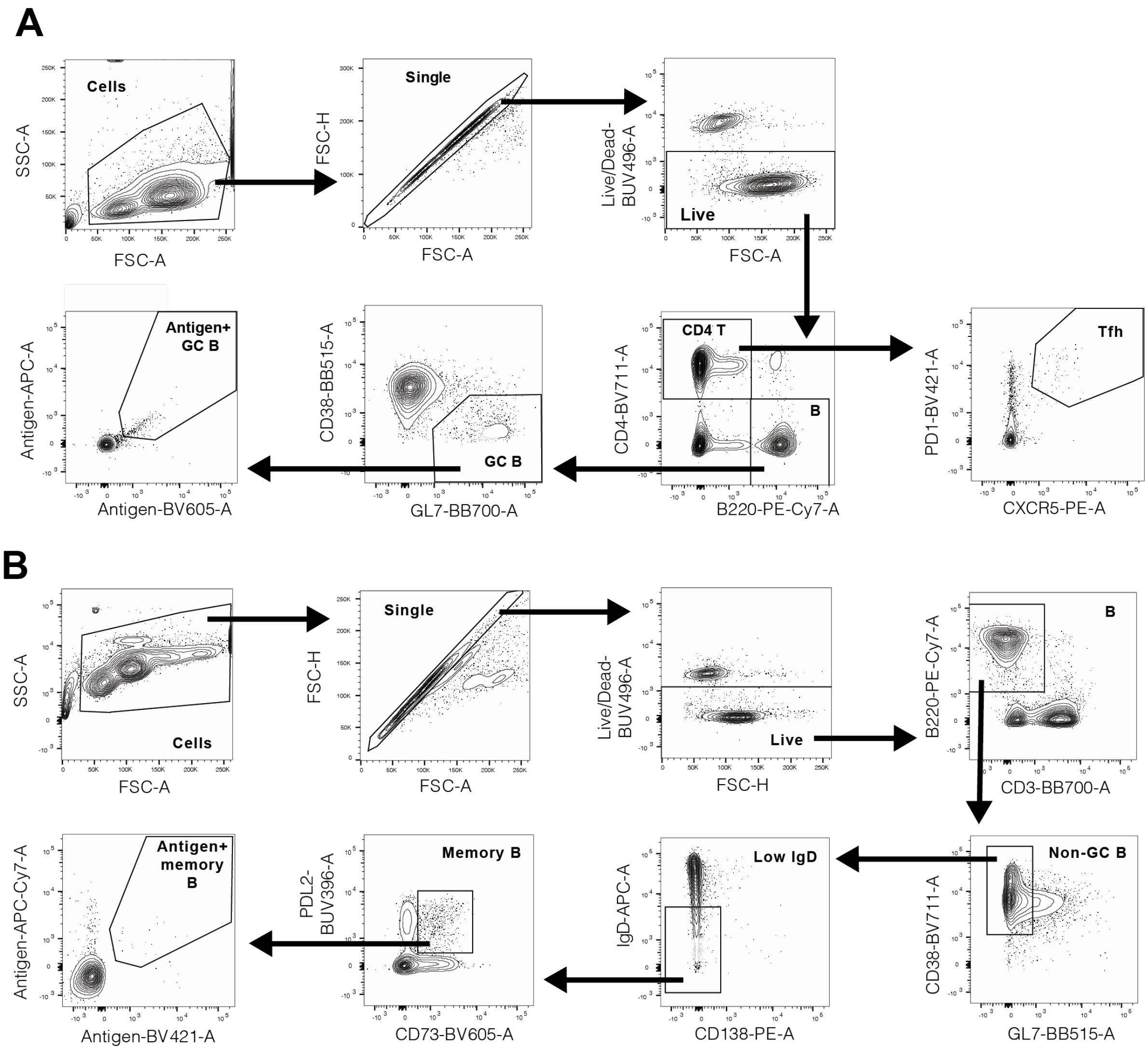

Figure S2. Gating for identification of Tfh, GC B cells, and memory B cells. (A) Flow cytometry plots showing the gating strategy used to identify T follicular helper cells and antigen-specific germinal center B cells in lymph nodes. (B) Flow cytometry plots showing the gating strategy used to identify memory B cells in spleens.

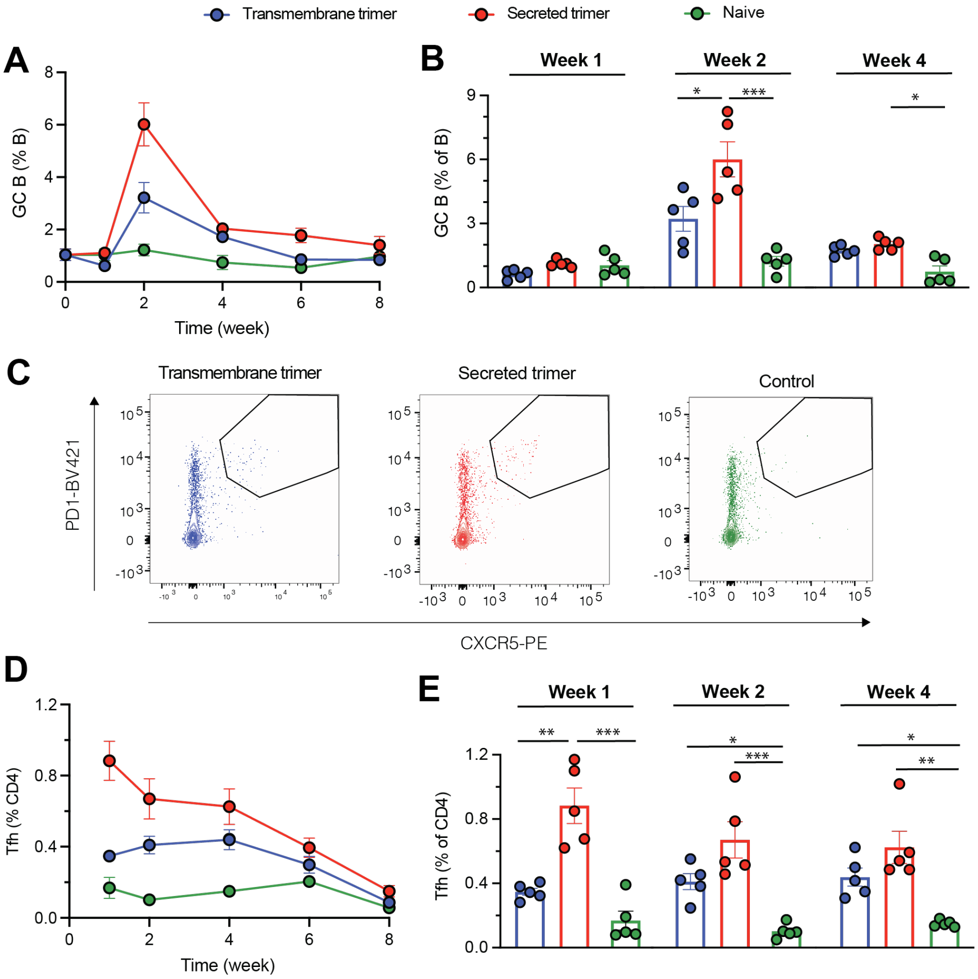

Figure S3. Time course analysis of germinal center responses to vaccination. GC B cell and Tfh populations were assessed in draining lymph nodes at various time points after a single immunization of BALB/c mice with LNP-replicons encoding the transmembrane or secreted trimer. **(A-B)** Percentage of GC B cell shown over time (A) and at individual time points (B). **(C)** Representative flow cytometry contour plots showing Tfh staining gated on CD4+ T cells from mice with indicated treatments at week 2 post immunization. **(D-E)** Percentage of Tfh cell shown over time (D) and at the specified individual time points (E). Statistical significance was assessed by two-way analysis of variance (ANOVA) followed by Tukey's post hoc test. Data are shown as mean ± SEM; *$p < 0.05$, **$p < 0.01$, and ***$p < 0.001$.

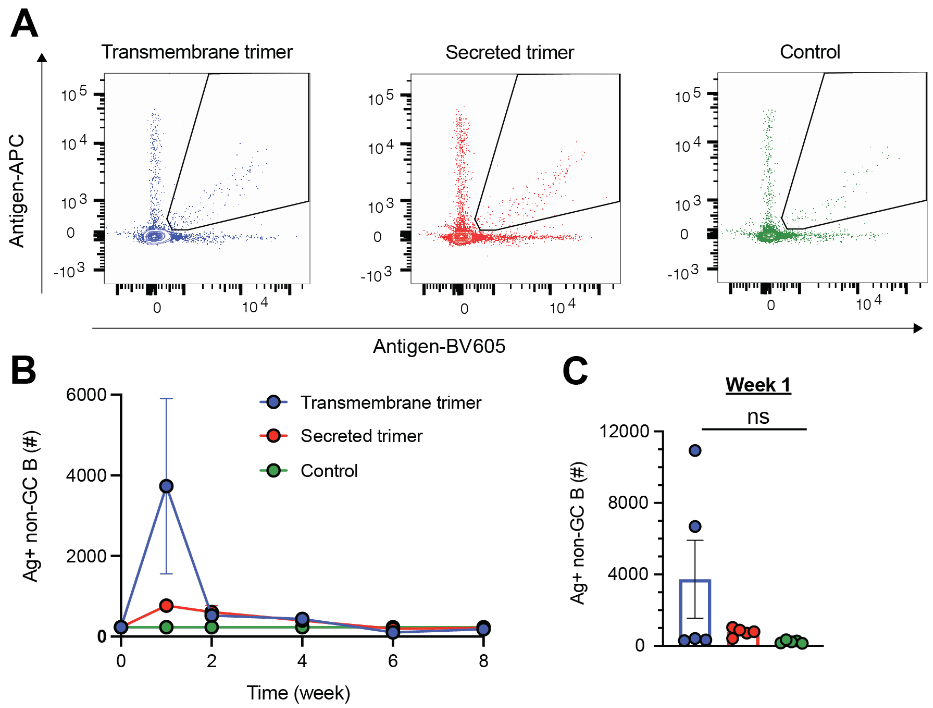

Figure S4. Trimer replicons elicited little to no extrafollicular/non-GC B cell response. Non-GC B cell populations were assessed in draining lymph nodes at various time points after a single immunization of BALB/c mice with LNP-replicons encoding the transmembrane or secreted trimer. **(A)** Representative flow cytometry contour plots showing antigen staining gated on non-GC B cells. **(B-C)** Number of antigen-specific non-GC B cells over time (B) and at individual time points (C). Statistical significance was assessed by two-way analysis of variance (ANOVA) followed by Tukey's post hoc test. Data are shown as mean ± SEM; *p < 0.05, **p < 0.01, and ***p < 0.001.

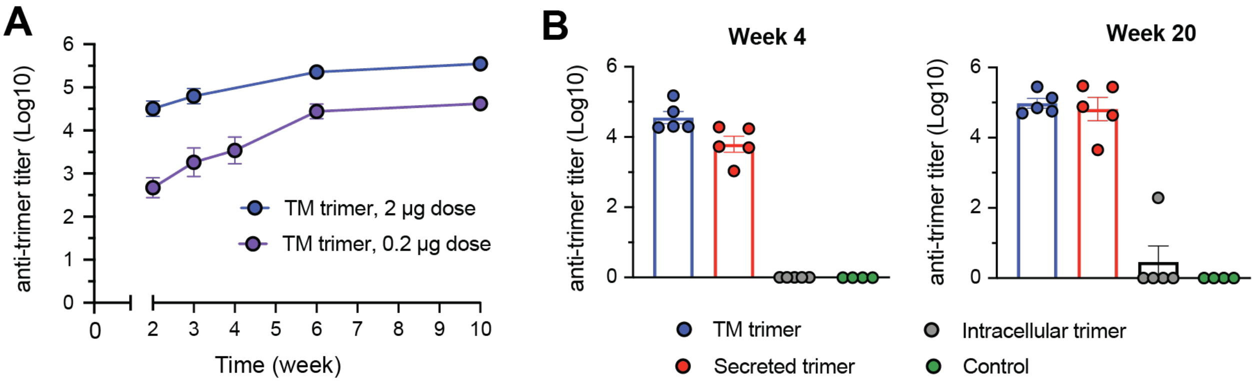

Figure S5. Humoral immune responses to vaccination. (A) Impact of the replicon dosage on the antibody titers elicited by Env trimer expressed from replicons. **(B)** Antibody responses to the intracellular form of the trimer compared to TM and secreted forms.

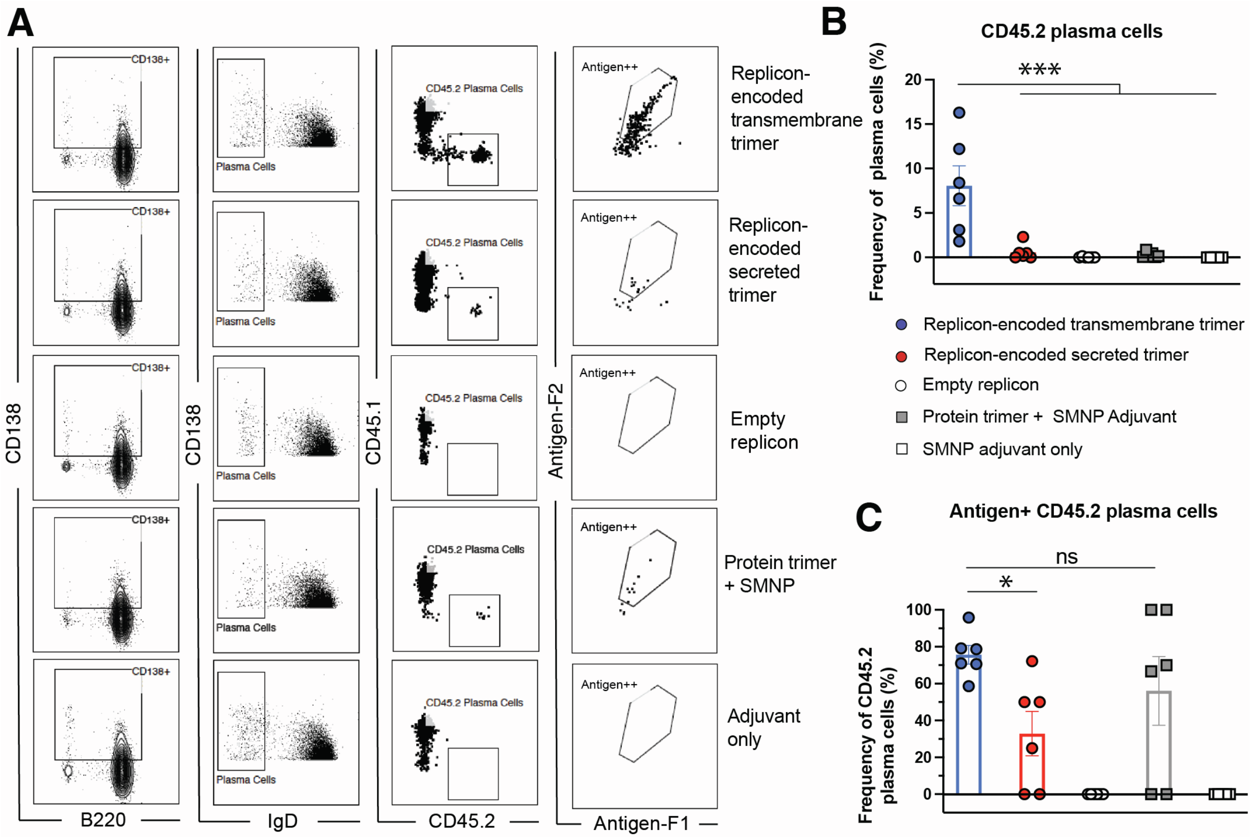

Figure S6. Plasma cell responses elicited by replicons in a precursor knockin mouse model. 1 x 10^5 B cells from BG18 $IgH^{+/WT}$ $CD45.2^+$ mice were adoptively transferred into $CD45.1^+$ recipient mice and were used in downstream immunization experiments. (A) Representative FACS plots showing plasma cells ($CD138^+$ IgD^-), $CD45.2^+$ plasma cells, and antigen-specific $CD45.2^+$ plasma cells at day 14 post immunization with empty replicon, transmembrane trimer-encoding replicon, secreted trimer-encoding replicon, protein trimer with SMNP adjuvant, or adjuvant alone. (B) Quantification of CD45.2 cells among toral plasma cells. (C) Trimer-specific plasma cell responses. Statistical significance was assessed by two-way analysis of variance (ANOVA) followed by Tukey's post hoc test. Data are shown as mean ± SEM; ***$p < 0.001$.

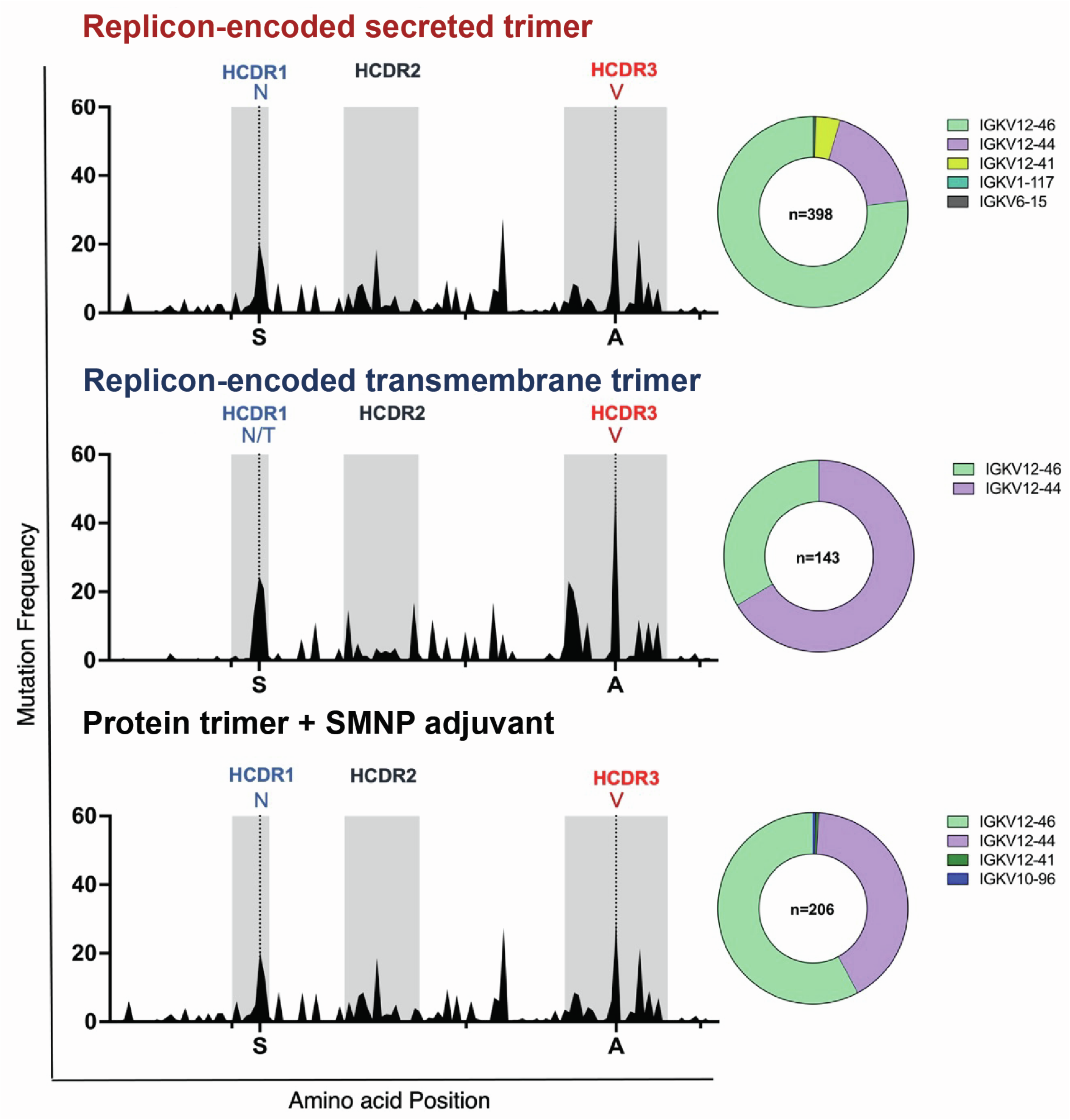

Figure S7. Sequence analysis from sorted BG18 IgH$^{+/WT}$ CD45.2^{+} Antigen^{+} cells at week 4 post immunization. Mutation distribution in heavy chain is represented in Hotspot Analysis (left) and associated murine light chain usage is represented (right panel).

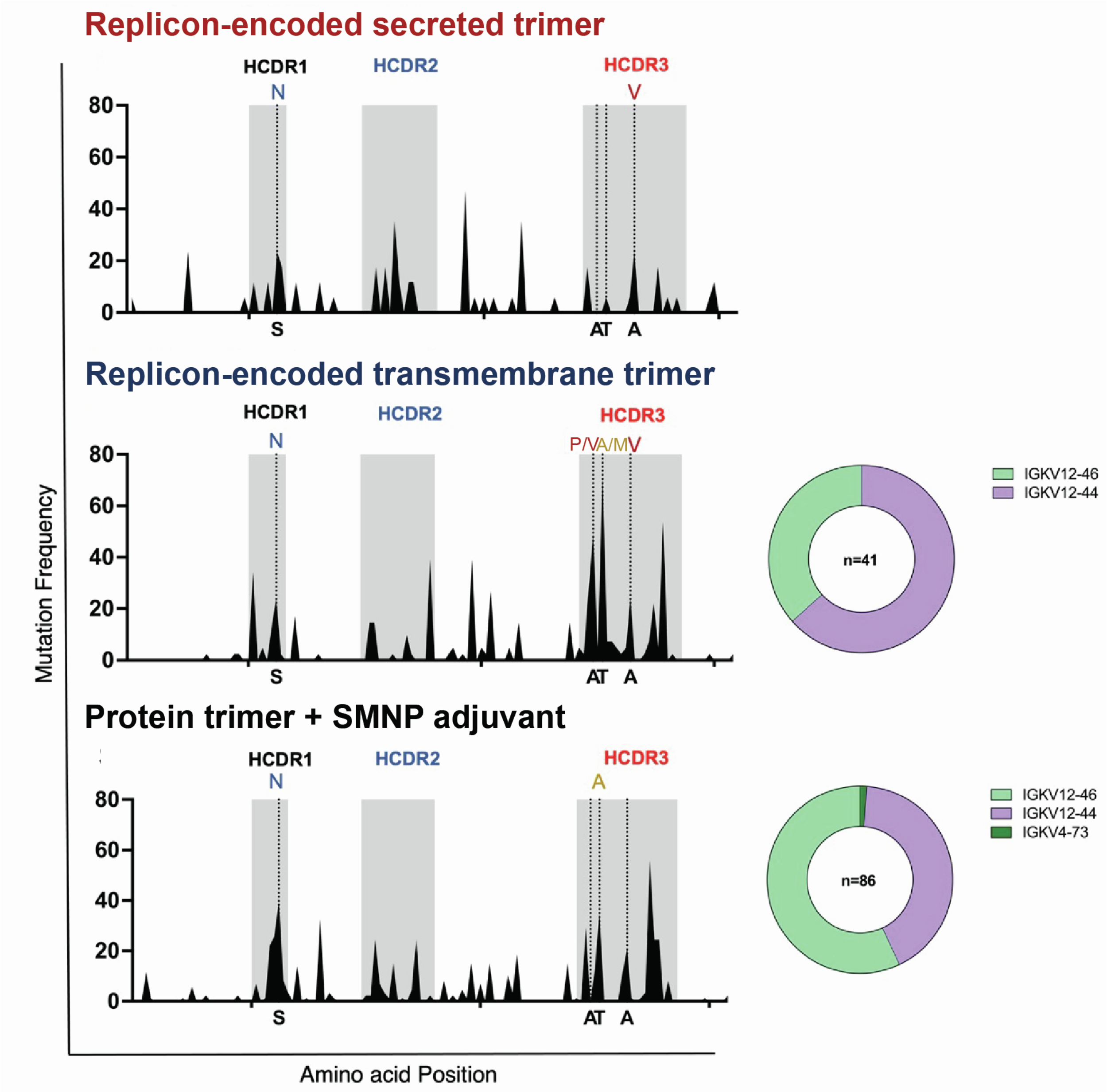

Figure S8. Sequence analysis from sorted BG18 IgH$^{+/WT}$ CD45.2^{+} Antigen^{+} cells at week 6 post immunization. Mutation distribution in heavy chain is represented in Hotspot Analysis (left) and associated murine light chain usage is represented (right panel).

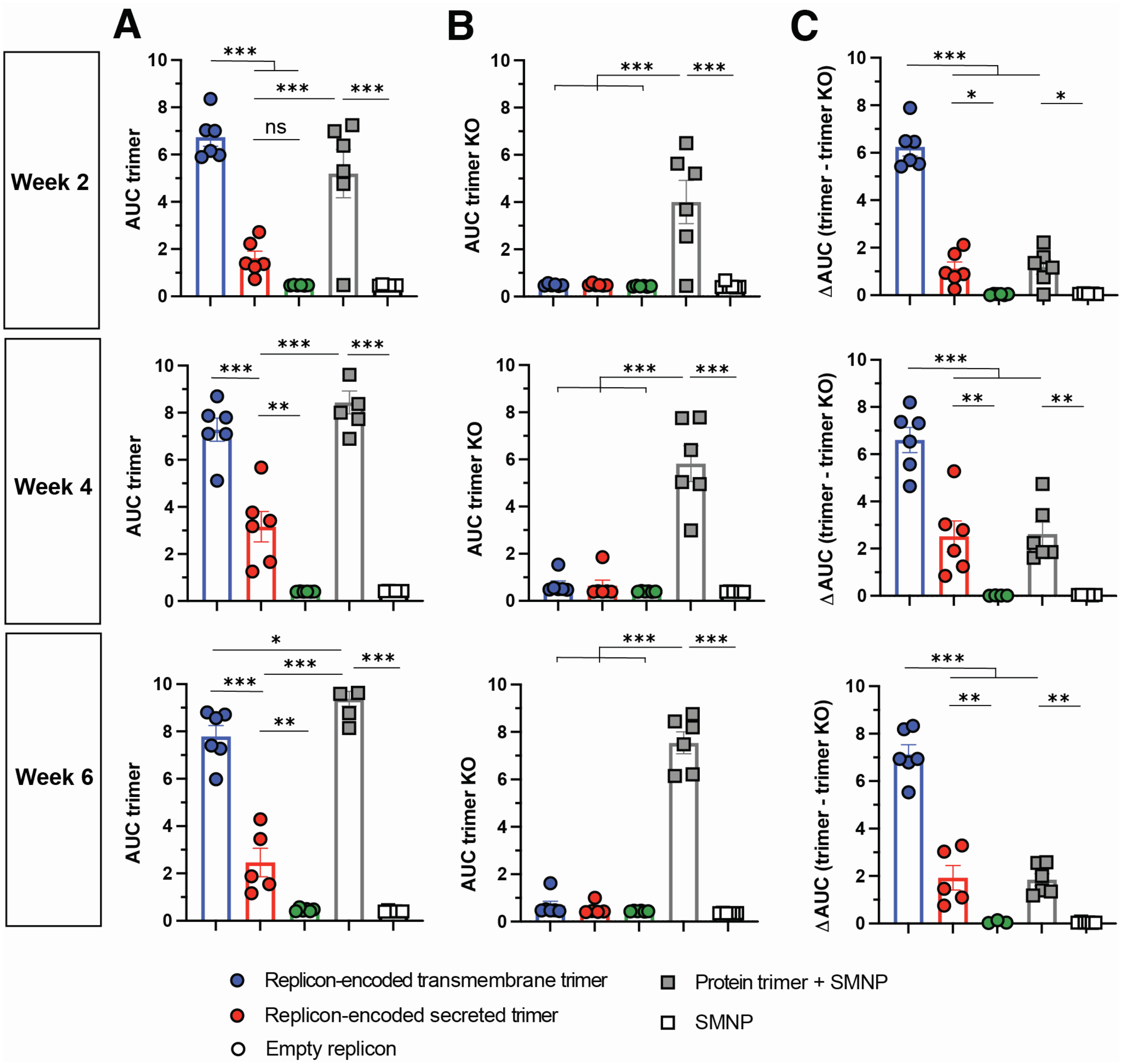

Figure S9. ELISA titer. ELISA reactivity to N332-GT2 and N332-GT2-KO trimers. Sera were collected from CD45.1^{+} mice adoptively transferred with B cells from BG18 IgH$^{+/WT}$ CD45.2^{+} mice at week 2, 4, and 6 post immunization and serum antibodies binding to N332-GT2 and N332-GT2-KO trimers were quantified by ELISA. Epitope targeting antibody responses were calculated by subtracting the AUC for the N332-GT2 KO trimer from the AUC for the N332-GT2 trimer. Statistical significance was assessed by two-way analysis of variance (ANOVA) followed by Tukey's post hoc test. Data are shown as mean ± SEM; *$p < 0.05$, **$p < 0.01$, and ***$p < 0.001$.
