# Supplementary material for: Augmented germinal center and antibody responses to HIV Env trimers delivered as transmembrane immunogens by self-replicating RNA
Source: Mol Ther. Author manuscript; Available in PMC 2025 Sep 12. (PMC12425358; doi:10.1016/j.ymthe.2025.07.036)
Supplement: Supplementary file 1 [file NIHMS2105229-supplement-1.pdf]

SUPPLEMENTAL INFORMATION

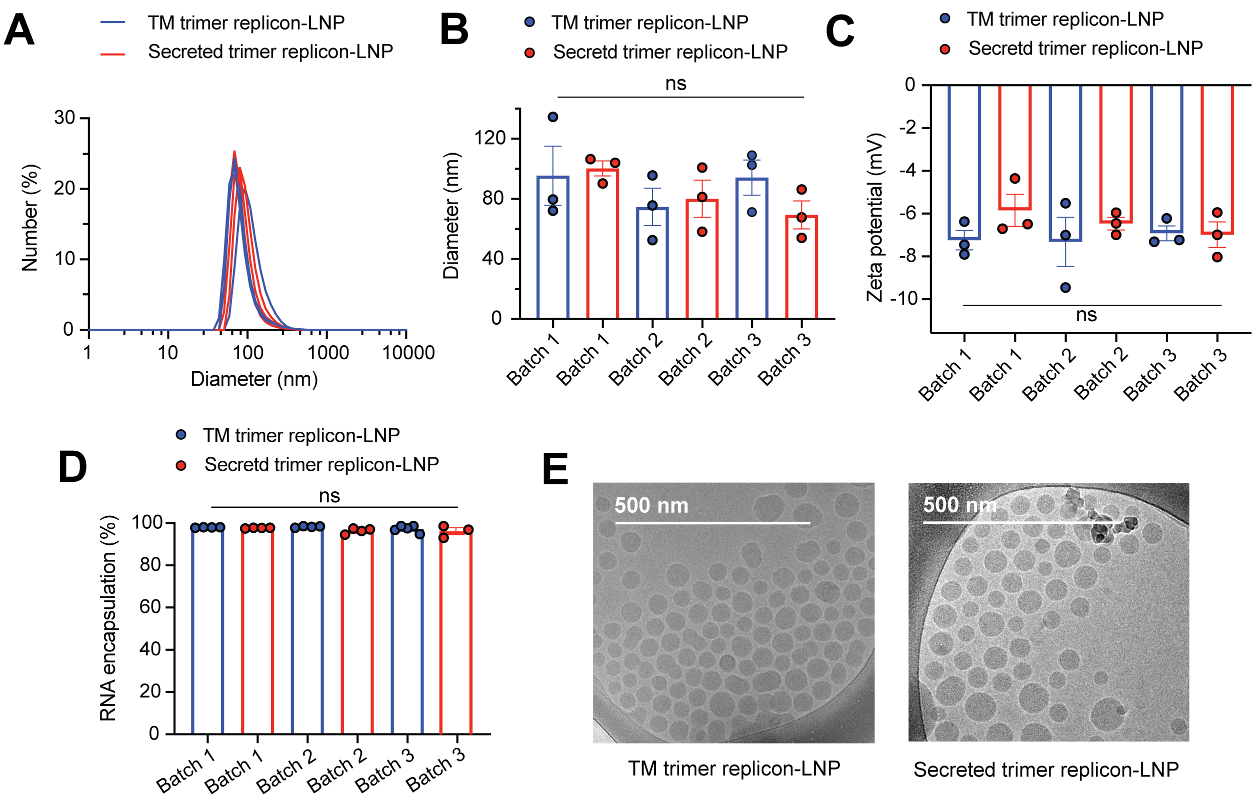

Figure S1. Characterization of LNPs encapsulating replicons encoding TM or secreted HIV Env trimers. (A) Representative dynamic light scattering (DLS) histogram showing number-based particle size distribution from three independently prepared batches. (B–D) Quantification of LNP size, reported as median diameter from the number distribution shown in S1A. (B), zeta potential (C), and RNA encapsulation efficiency (D) across three independent batches for each formulation. (E) Cryo-transmission electron microscopy (cryo-TEM) images of LNPs encapsulating TM or secreted trimer replicons. Statistical comparisons were performed by two-way analysis of variance (ANOVA) followed by Tukey's post hoc test. Data are shown as mean ± SEM.

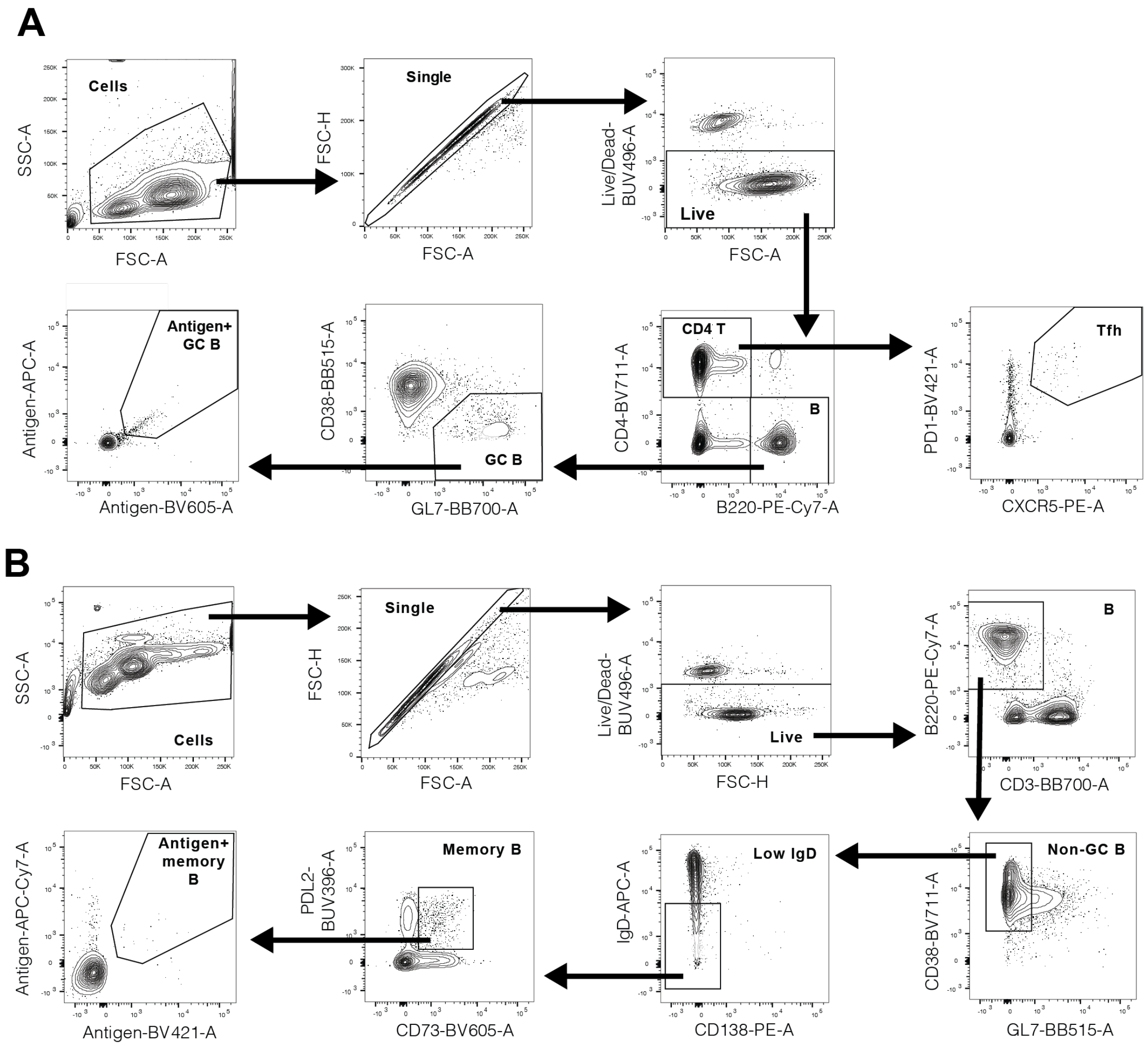

Figure S2. Gating for identification of Tfh, GC B cells, and memory B cells. (A) Flow cytometry plots showing the gating strategy used to identify T follicular helper cells and antigen-specific germinal center B cells in lymph nodes. (B) Flow cytometry plots showing the gating strategy used to identify memory B cells in spleens.

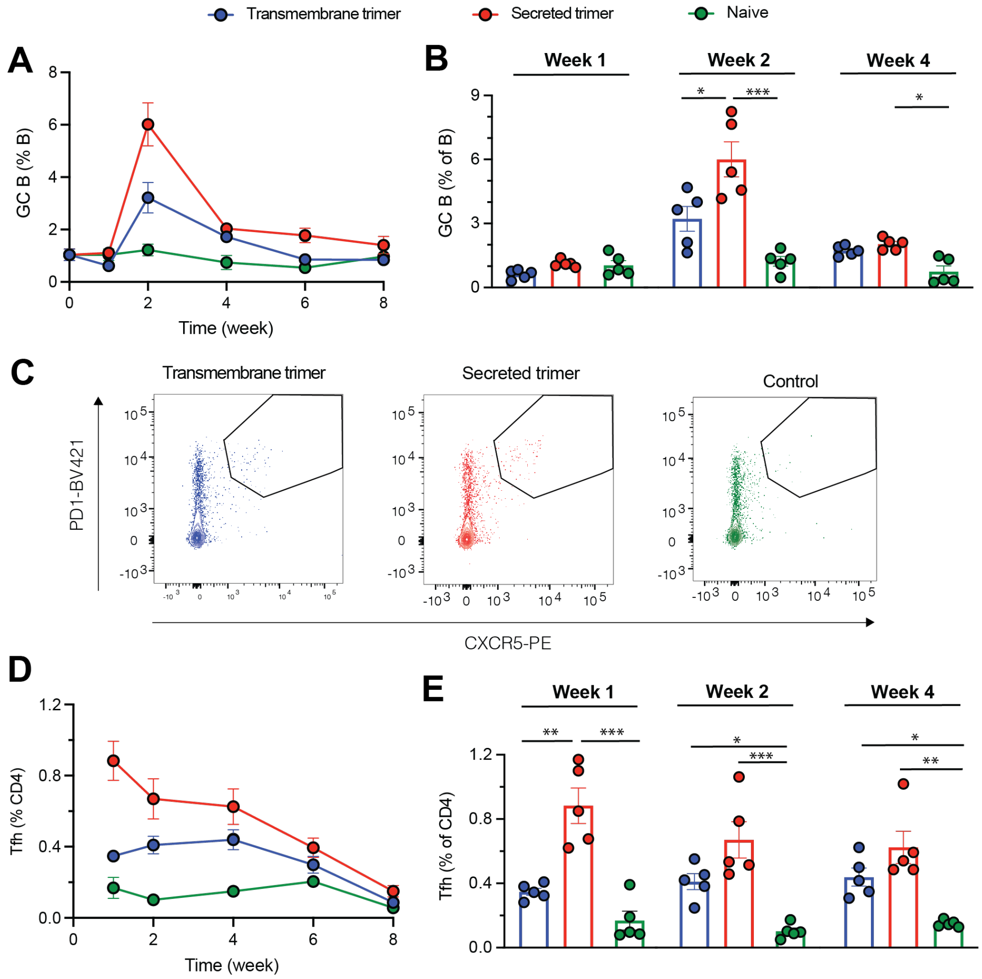

Figure S3. Time course analysis of germinal center responses to vaccination. GC B cell and Tfh populations were assessed in draining lymph nodes at various time points after a single immunization of BALB/c mice with LNP-replicons encoding the transmembrane or secreted trimer. **(A-B)** Percentage of GC B cell shown over time (A) and at individual time points (B). **(C)** Representative flow cytometry contour plots showing Tfh staining gated on CD4+ T cells from mice with indicated treatments at week 2 post immunization. **(D-E)** Percentage of Tfh cell shown over time (D) and at the specified individual time points (E). Statistical significance was assessed by two-way analysis of variance (ANOVA) followed by Tukey's post hoc test. Data are shown as mean ± SEM; *$p < 0.05$, **$p < 0.01$, and ***$p < 0.001$.

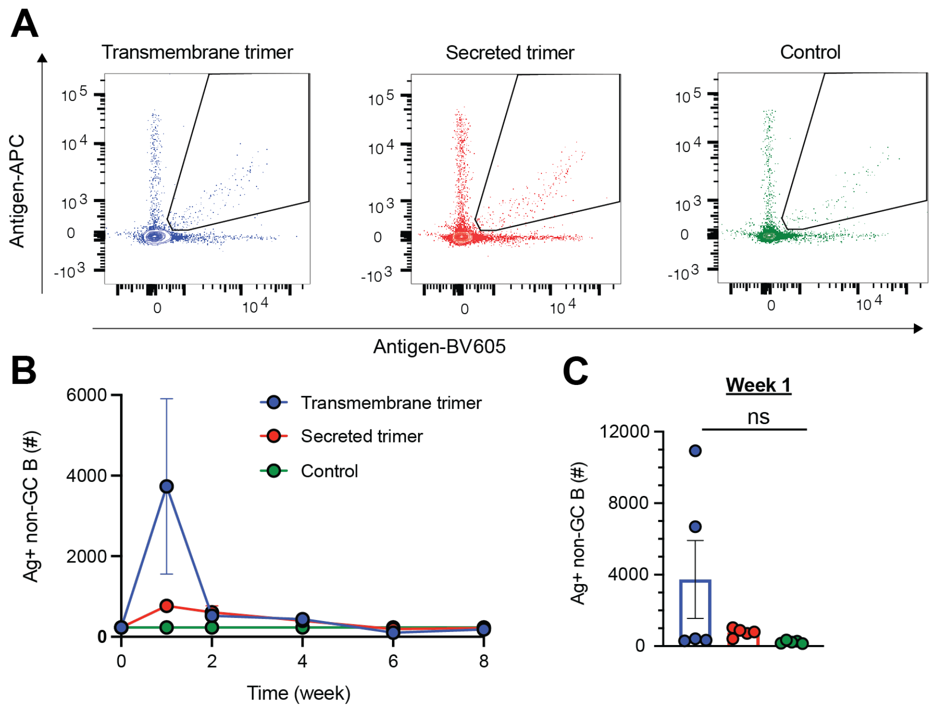

Figure S4. Trimer replicons elicited little to no extrafollicular/non-GC B cell response. Non-GC B cell populations were assessed in draining lymph nodes at various time points after a single immunization of BALB/c mice with LNP-replicons encoding the transmembrane or secreted trimer. **(A)** Representative flow cytometry contour plots showing antigen staining gated on non-GC B cells. **(B-C)** Number of antigen-specific non-GC B cells over time (B) and at individual time points (C). Statistical significance was assessed by two-way analysis of variance (ANOVA) followed by Tukey's post hoc test. Data are shown as mean ± SEM; *$p < 0.05$, **$p < 0.01$, and ***$p < 0.001$.

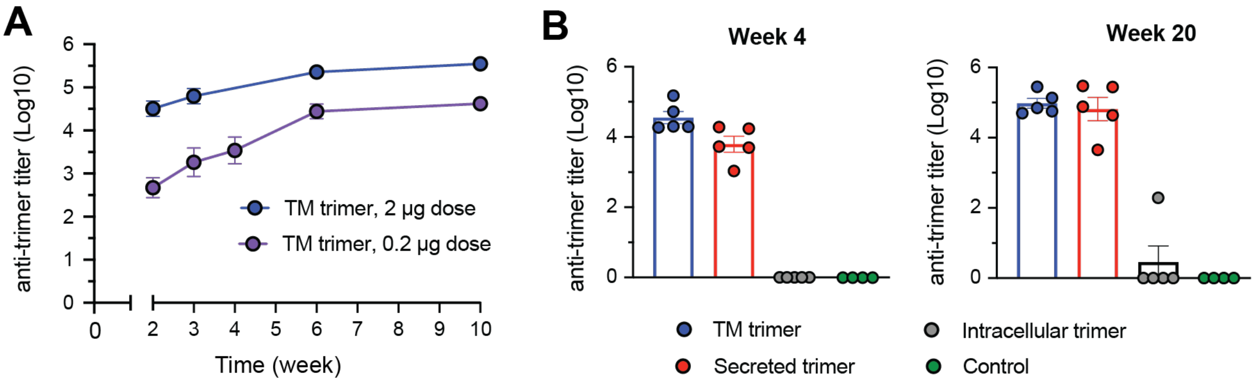

Figure S5. Humoral immune responses to vaccination. (A) Impact of the replicon dosage on the antibody titers elicited by Env trimer expressed from replicons. **(B)** Antibody responses to the intracellular form of the trimer compared to TM and secreted forms.

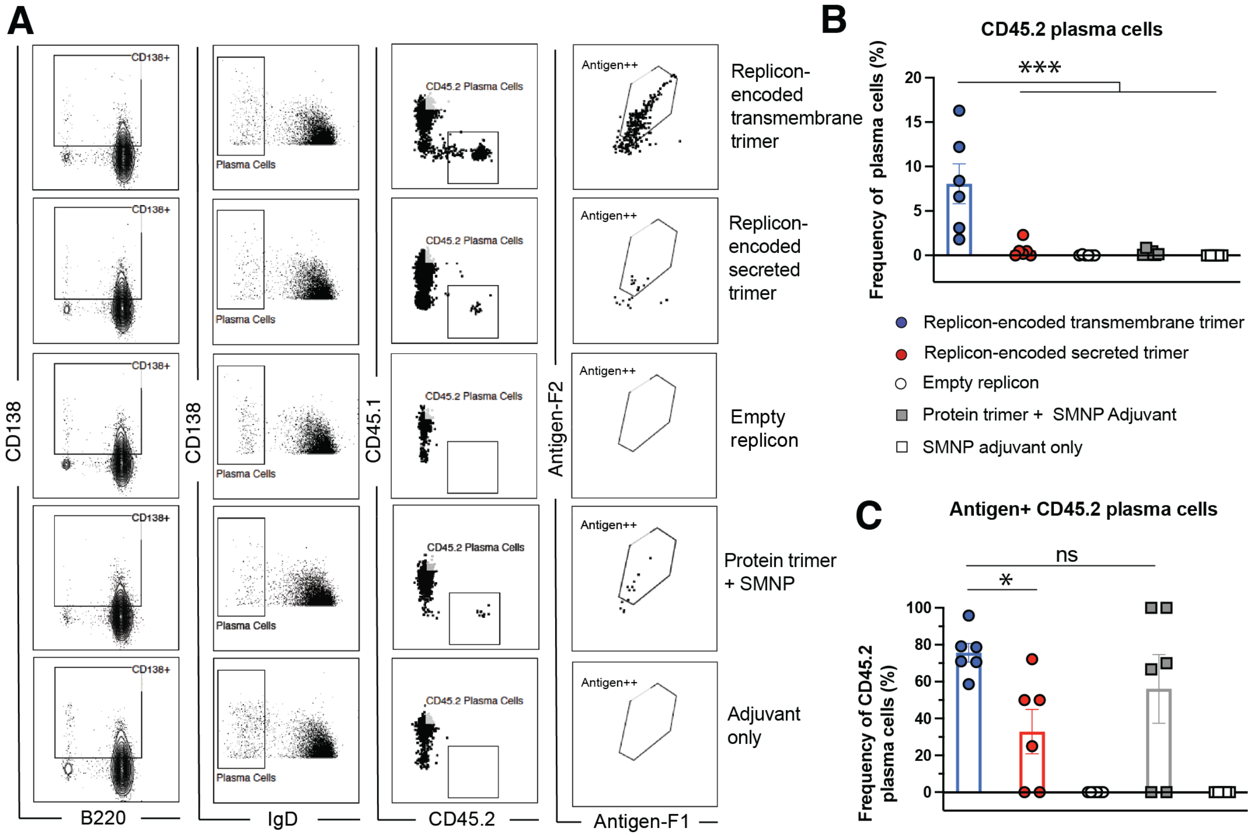

Figure S6. Plasma cell responses elicited by replicons in a precursor knockin mouse model. 1 x 10^5 B cells from BG18 $IgH^{+/WT}$ $CD45.2^{+}$ mice were adoptively transferred into $CD45.1^{+}$ recipient mice and were used in downstream immunization experiments. (A) Representative FACS plots showing plasma cells ($CD138^{+}$ IgD^{-}), $CD45.2^{+}$ plasma cells, and antigen-specific $CD45.2^{+}$ plasma cells at day 14 post immunization with empty replicon, transmembrane trimer-encoding replicon, secreted trimer-encoding replicon, protein trimer with SMNP adjuvant, or adjuvant alone. (B) Quantification of CD45.2 cells among toral plasma cells. (C) Trimer-specific plasma cell responses. Statistical significance was assessed by two-way analysis of variance (ANOVA) followed by Tukey's post hoc test. Data are shown as mean ± SEM; ***$p < 0.001$.

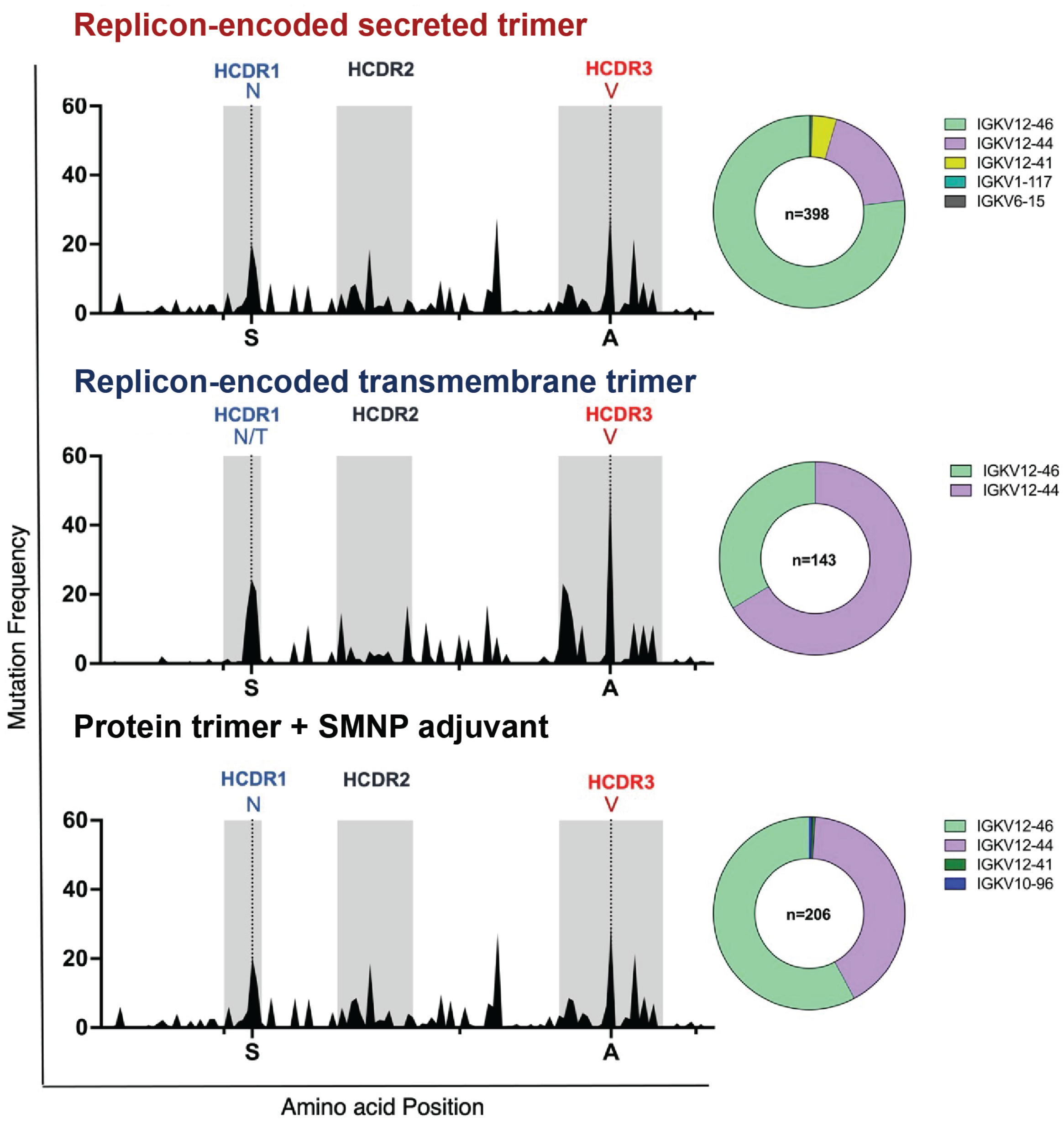

Figure S7. Sequence analysis from sorted BG18 IgH$^{+/WT}$ CD45.2^{+} Antigen^{+} cells at week 4 post immunization. Mutation distribution in heavy chain is represented in Hotspot Analysis (left) and associated murine light chain usage is represented (right panel).

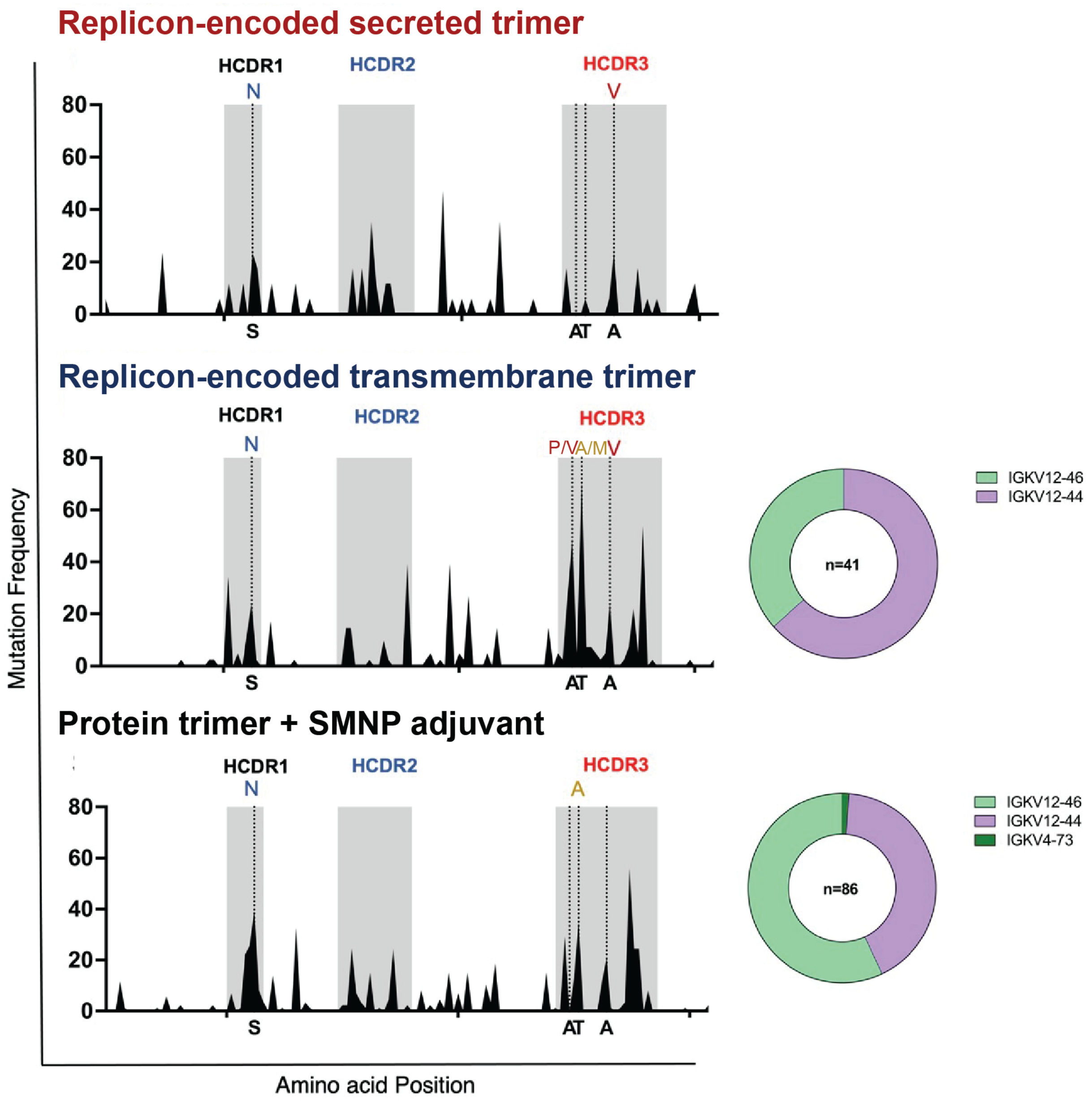

Figure S8. Sequence analysis from sorted BG18 IgH$^{+/WT}$ CD45.2^{+} Antigen^{+} cells at week 6 post immunization. Mutation distribution in heavy chain is represented in Hotspot Analysis (left) and associated murine light chain usage is represented (right panel).

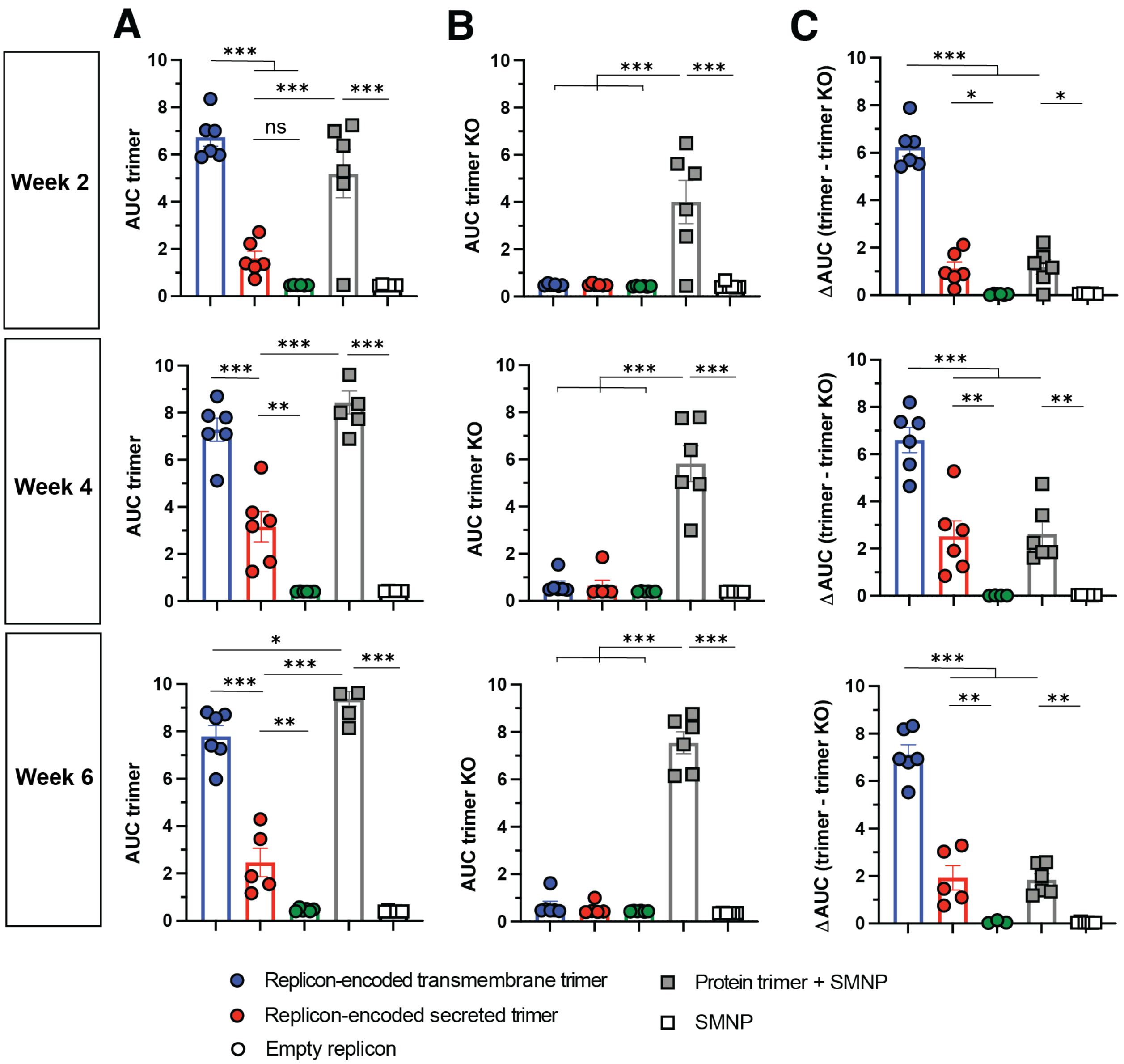

Figure S9. ELISA titer. ELISA reactivity to N332-GT2 and N332-GT2-KO trimers. Sera were collected from CD45.1^{+} mice adoptively transferred with B cells from BG18 IgH$^{+/WT}$ CD45.2^{+} mice at week 2, 4, and 6 post immunization and serum antibodies binding to N332-GT2 and N332-GT2-KO trimers were quantified by ELISA. Epitope targeting antibody responses were calculated by subtracting the AUC for the N332-GT2 KO trimer from the AUC for the N332-GT2 trimer. Statistical significance was assessed by two-way analysis of variance (ANOVA) followed by Tukey's post hoc test. Data are shown as mean ± SEM; *$p < 0.05$, **$p < 0.01$, and ***$p < 0.001$.
